# Solvent and additive-controlled supramolecular isomerism in zinc coordination polymers

**DOI:** 10.1038/s41598-024-77298-6

**Published:** 2024-11-11

**Authors:** Ghazale Khorshidi, Behrouz Notash

**Affiliations:** Department of Inorganic Chemistry, Shahid Beheshti University, Tehran, 1983969411 Iran

**Keywords:** Inorganic chemistry, Solid-state chemistry, Coordination chemistry, Chemistry, Metal-organic frameworks

## Abstract

A new series of Zn(II) supramolecular isomers containing ditopic 1,3-di(pyridin-4-yl)urea (**4bpu**) ligand were synthesized and characterized by infrared analysis, elemental analysis and TGA as well as single-crystal X-ray diffraction analysis. Four solvent-induced pseudopolymorphic zinc (II) coordination polymers (CPs), namely, {[Zn(4bpu)(OAc)_2_](CH_3_OH)}_n_ (**1**), {[Zn(4bpu)(OAc)_2_](C_2_H_5_OH)}_n_ (**2**), {[Zn(4bpu)(OAc)_2_](HOCH_2_CH_2_OH)}_n_ (**3**), and {[Zn(4bpu)(OAc)_2_](0.5H_2_O)}_n_ (**4**), were prepared by the reaction of Zn(OAc)_2_.2H_2_O and **4bpu** via self-assembly under varying solvent systems. Also, a pair of polymorphic coordination polymers namely, {[Zn(4bpu)(OAc)_2_](CH_3_OH)}_n_ (**1α**) and {[Zn_3_(4bpu)_3_(OAc)_6_](CH_3_OH)_2_}_n_**(1β)**, was prepared in the presence of different organic additives. Single-crystal X-ray diffraction confirmed that **1−4** and **1α** display 1D polymeric zig-zag chains and **1β** exhibits 1D triple-stranded ladders that were self-assembled through various supramolecular interactions. In addition, a series of dissolution-recrystallization structural transformations (DRST) were performed on these supramolecular isomers.

**Supplementary Information:**

The online version contains supplementary material available at 10.1038/s41598-024-77298-6.

## Introduction

Over the past few decades, the design and synthesis of organic − inorganic hybrid systems (OIHSs), such as metal-organic frameworks (MOFs) and coordination polymers (CPs), have been motivated by their structural diversities, intriguing topologies, and great potential applications^1,2^, including gas storage and separation^3,4^, catalysis^5–7^, magnetism^8,9^, sensing^10–12^, as well as bioimaging and drug delivery^13,14^.

Enhancement of desired properties of materials is often achieved through functionalization^15–17^. Recently significant attention has been dedicated to developing functionalized OIHSs with urea groups, due to the conformational flexibility, strong hydrogen bonding capabilities, and polarizability^18–21^. However, the intermolecular self-association process among urea moieties (urea⋯urea hydrogen bonding α-tape motif), attributed to the robust hydrogen-bond donating and accepting capability of the urea functional group, complicates the design and synthesis of urea systems^22,23^. So, synthetic route and crystallization conditions could be of great importance for the controlling and interruption of the urea α-tape hydrogen bonding motif. Also, the formation of new and various supramolecular synthons provide new insights into the design and synthesis of new compounds and supramolecular isomers^24–26^. In the crystalline state, supramolecular isomerism is defined as two or more supramolecular systems with identical stoichiometry, composition, and building blocks (such as metal ions and organic linkers) that generate different architectures^27,28^. Supramolecular isomerism can be divided into four main classes, which include structural, conformational, optical, and catenane isomerism. Additionally, there are two subsets: polymorphic and pseudopolymorphic supramolecular isomers^28–31^. Moreover, in the case of pseudopolymorphs or solvatomorphs that can differ in the number or type of guest or coordinated solvent(s) in the structure, the role of solvent in the construction of supramolecular isomers is highlighted^28,32,33^. This phenomenon has become one of the main subjects of study in recent years due to its ability to provide valuable information on the factors influencing the self-assembly process and the structure-property relationships^34,35^. Also, polymorphism and pseudopolymorphism are common phenomena in many areas of solid-state chemistry and they are especially important in the pharmaceutical industries because polymorphism of the active pharmaceutical ingredients (APIs) are frequently found to have a significant impact on their qualities. Consequently, controlling and tuning on the polymorphism and pseudopolymorphism holds great significance^36,37^. In this context, the resulting supramolecular isomers are determined by several factors, such as temperature^38,39^, solvent^40–44^, pH^45,46^, molar ratio^47,48^, concentration^49,50^, additive or template^51–56^. In a few cases, additive or template molecules can play a critical role in the formation of supramolecular isomers^57,58^. Du and co-workers investigated additive-induced supramolecular isomerism by properly selecting the organic acids such as adipic acid and d, l-mandelic acid as additive agents^58^. Among the mentioned factors, the solvent system, in addition to playing an important role as a reaction medium, can also act as a structure guiding factor and lead to the formation of different supramolecular isomers^59^.

Non-covalent interactions as a design element have crucial role in controlling the self-assembly and construction of supramolecular systems and coordination polymers^60–62^. Recently, the interesting attraction between group 12 elements and electron-rich atoms such as Lewis bases or anions has been termed as the spodium bond^63^. The Zn(II) complexes have the tendency to self-assemble in the solid-state through spodium bonds to form supramolecular architectures^64–67^.

Although solid-state SC-SC structural transformations between supramolecular isomers are highly regarded^68,69^, there are few reports on the solvent-induced structural transformation through a dissolution–recrystallization structural transformation (DRST)^30,32,70,71^.

Herein, a hydrogen-bond-functionalized ligand 1,3-di(pyridin-4-yl)urea (**4bpu**) and Zn(OAc)_2_.2H_2_O has been applied in constructing four Zn(II) pseudopolymorphic CPs with the formulas {[Zn(4bpu)(OAc)_2_](CH_3_OH)}_n_ (**1**), {[Zn(4bpu)(OAc)_2_](C_2_H_5_OH)}_n_ (**2**), {[Zn(4bpu)(OAc)_2_](HOCH_2_CH_2_OH)}_n_ (**3**) and {[Zn(4bpu)(OAc)_2_](0.5H_2_O)}_n_ (**4**) by layering method with various solvents. Moreover, in this study, we report controllable synthesis of two additive-induced supramolecular isomers of compound **1**, with the formulas {[Zn(4bpu)(OAc)_2_](CH_3_OH)}_n_ (**1α**) and {[Zn_3_(4bpu)_3_(OAc)_6_](CH_3_OH)_2_}_n_**(1β)**. In addition, solvent-mediated DRST structural transformations between supramolecular isomers are performed.

## Experimental section

### Materials and instruments

All reagents and solvents used for synthesis were obtained from commercial sources and were used directly without further purification. The ligand 1,3-di(pyridin-4-yl)urea (**4bpu**) was synthesized following the reported literature^72^. The detailed synthetic procedure and spectral analysis are described in the Supplementary Information (Figures S1**−**S3). ^1^HNMR and ^13^CNMR spectra were recorded using a 300 and 75 MHz spectrometer, respectively (Bruker DRX-300 Avance). Infrared spectra were recorded on a MB102 Bomem spectrometer with KBr pellets in the 400 to 4000 cm^−1^ region. A Thermo Nicolet Nexus 470 was utilized for taking attenuated total reflectance Fourier transform infrared (ATR-FTIR) spectra in the region of 650 to 4000 cm^−1^. Melting points were determined on an Electrothermal 9100 melting point apparatus and were uncorrected. The X-ray powder diffraction patterns (PXRD) were collected on a STADIP STOE apparatus. The scanning electron microscopy (SEM) images were collected on a HITACHI SU3500 Scanning Electron Microscope using a 15 kV energy source under vacuum. Samples were transferred to conductive carbon tape on a sample holder disk and coated with a 10 nm thick Au layer using sputtering. Elemental analyses (C, H, N) were determined on a Vario EL III elemental analyzer. TGA analysis was carried out using a Mettler Toledo Star SW 10.00 instrument under a flowing N_2_ atmosphere at a heating rate of 10 °C/min.

### Synthesis of {[Zn(4bpu)(OAc)_2_](CH_3_OH)}_n_ (1)

**Scheme 1:**
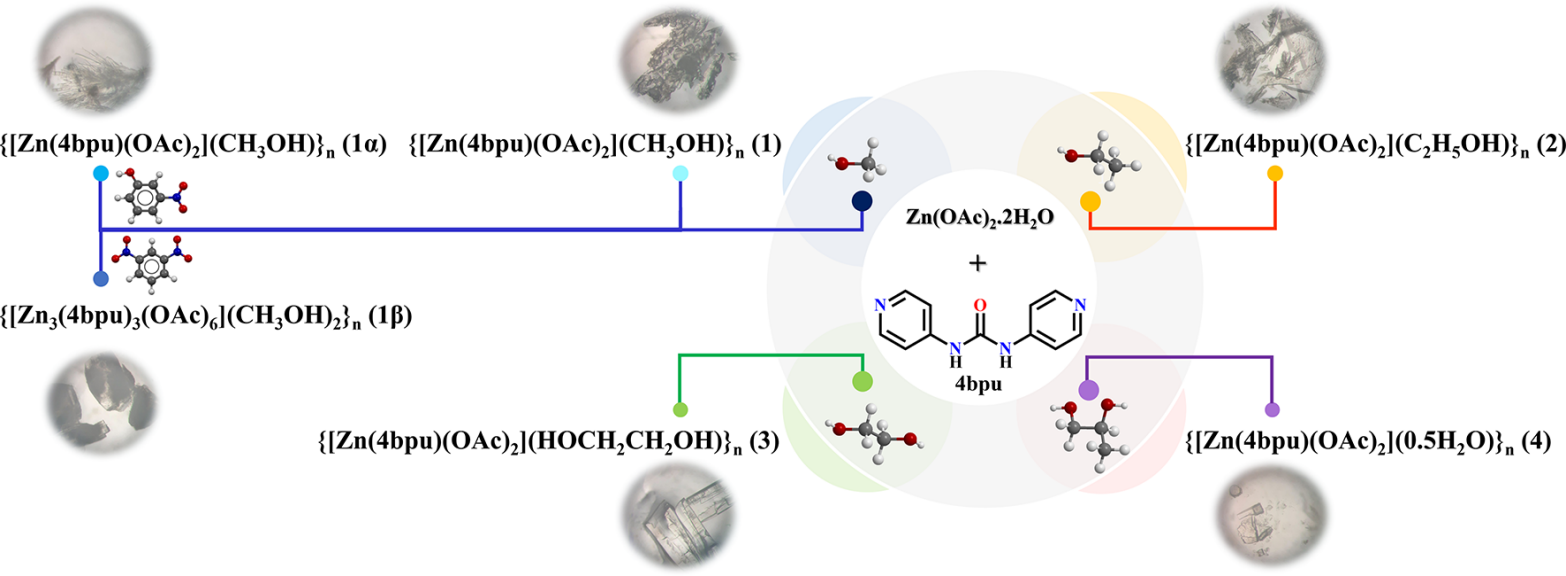
Synthesis route and optical microscopic images of CPs **1-4**, **1α** and **1β**.

A total of 0.0107 g (0.05 mmol) of **4bpu** was completely dissolved in 1 mL of methanol and transferred to a glass test tube, suitable for a layering method. Then 1 mL of methanol as a buffer layer was added to the tube. A total of 0.0109 g (0.05 mmol) of Zn(OAc)_2_.2H_2_O, completely dissolved in 1 mL of methanol, was slowly added to the tube as the third layer. After one day, colorless plate-shaped single crystals of compound **1** were obtained (Scheme 1). Yield: 65%. mp > 260 °C. Elemental analysis for compound **1** (C_16_H_20_N_4_O_6_Zn): Calcd: C, 44.72; H, 4.69; N, 13.04%; Found: C, 44.22; H, 3.97; N, 13.85%. IR data (KBr pellet, cm^−1^): 3499(m), 3448(m), 3290(m), 3201(m), 3181(m), 3085(m), 3028(m), 2505(w), 2358(w), 1946(w), 1746(s), 1594(s), 1521(s), 1413(s), 1331(s), 1292(s), 1255(m), 1182(s), 1059(m), 1029(s), 935(w), 906(w), 852(s), 834(s), 798(w), 768(w), 726(m), 687(m), 680(m), 664(m), 622(m), 553(m), 545(m), 533(s), 497(w) (Figure S4).

### Synthesis of {[Zn(4bpu)(OAc)_2_](C_2_H_5_OH)}_n_ (2)

Compound **2** was prepared in a similar way as described for **1** except that ethanol was used instead of methanol as solvent. After one day, colorless plate-shaped single crystals of compound **2** were obtained (Scheme 1). Yield: 60%. mp > 260 °C. Elemental analysis for compound **2** (C_17_H_22_N_4_O_6_Zn): Calcd: C, 46.01; H, 5.00; N, 12.63%; Found: C, 43.91; H, 4.17; N, 13.71%. IR data (KBr pellet, cm^−1^): 3449(w), 3303(m), 3176(m), 3084(m), 3029(m), 2359(w), 1746(m), 1595(s), 1521(s), 1505(s), 1434(s), 1413(s), 1337(m), 1292(m), 1261(w), 1188(s), 1063(w), 1026(s), 935(w), 906(w), 852(m), 834(s), 798(w), 768(w), 734(m), 725(m), 684(m), 677(m), 664(w), 622(w), 553(m), 545(m), 535(s), 496(w) (Figure S5).

### Synthesis of {[Zn(4bpu)(OAc)_2_](HOCH_2_CH_2_OH)}_n_ (3)

Compound **3** was prepared in a similar way as described for **1** except that ethylene glycol was used instead of methanol. After two days, colorless needle-shaped single crystals of compound **3** were obtained. (Scheme 1). Yield: 65%. mp > 260 °C. Elemental analysis for compound **3** (C_17_H_22_N_4_O_7_Zn): Calcd: C, 44.41; H, 4.82; N, 12.19%; Found: C, 44.36; H, 4.88; N, 12.20%. IR data (KBr pellet, cm^−1^): 3198(m), 3080(m), 2950(m), 2928(m), 2508(w), 1749(s), 1725(m), 1595(s), 1519(s), 1438(s), 1412(s), 1337(s), 1285(s), 1256(m), 1194(s), 1066(m), 1052(m), 1027(s), 934(w), 891(m), 834(s), 801(w), 735(m), 673(m), 620(m), 552(m), 529(s), 511(w), 502(w) (Figure S6).

### Synthesis of {[Zn(4bpu)(OAc)_2_](0.5H_2_O)}_n_ (4)

Compound **4** was synthesized by a different method as described before^73^ and was prepared in a similar way as mentioned for compound **1** except that propylene glycol was used as solvent. After one day, colorless plate-shaped single crystals of compound **4** were obtained. (Scheme 1). Yield: 63%. mp > 260 °C. Elemental analysis for compound **4** (C_30_H_34_N_8_O_11_Zn_2_): Calcd: C, 44.30; H, 4.21; N, 13.78%; Found: C, 43.97; H, 3.49; N, 13.67%. IR data (KBr pellet, cm^−1^): 3449(w), 3292(w), 3085(m), 3026(m), 1746(s), 1590(s), 1521(s), 1498(s), 1413(s), 1331(m), 1291(s), 1255(m), 1181(s), 1059(m), 1024(s), 935(w), 906(w), 834(s), 798(w), 768(w), 734(m), 725(m), 684(m), 677(m), 663(m), 623(m), 555(m), 543(m), 533(s), 496(w) (Figure S7).

### Synthesis of {[Zn(4bpu)(OAc)_2_](CH_3_OH)}_n_ (1α)

A total of 0.0107 g (0.05 mmol) of **4bpu** and 0.0278 g (0.2 mmol) of 3-nitrophenol was completely dissolved in 1 mL of methanol and transferred to a glass test tube, suitable for a layering method. Then 1 mL of methanol as a buffer layer was added to the tube. A total of 0.0109 g (0.05 mmol) of Zn(OAc)_2_.2H_2_O, completely dissolved in 1 mL of methanol, was slowly added to the tube as the third layer. After one day, light yellow needle-shaped single crystals of compound **1α** were obtained (Scheme 1). Yield: 60%. mp > 260 °C. Elemental analysis for compound **1α** (C_16_H_20_N_4_O_6_Zn): Calcd: C, 44.72; H, 4.69; N, 13.04%; Found: C, 43.29; H, 4.25; N, 13.52%. IR data (KBr pellet, cm^−1^): 3362(w), 3013(w), 1739(m), 1621(s), 1598(s), 1558(s), 1523(s), 1509(s), 1431(s), 1393(m), 1337(m), 1293(m), 1248(w), 1188(s), 1064(w), 1028(s), 928(w), 866(w), 837(s), 746(m), 684(m), 668(m), 619(w), 555(m), 535(m), 500(w), 420(w) (Figure S8).

### Synthesis of {[Zn_3_(4bpu)_3_(OAc)_6_](CH_3_OH)_2_]}_n_ (1β)

Compound **1β** was prepared in a similar way as described for **1α** except that 0.0336 g (0.2 mmol) 1,3-dinitrobenzene was used instead of 3-nitrophenol. After one day, colorless plate-shaped single crystals of compound **1β** were obtained (Scheme 1). Yield: 61%. mp > 260 °C. Elemental analysis for compound **1β** (C_47_H_56_N_12_O_17_Zn_3_): Calcd: C, 44.90; H, 4.49; N, 13.37%; Found: C, 44,09; H, 4.39; N, 13.04%. IR data (KBr pellet, cm^−1^): 3521(m), 3362(m), 3084(w), 3014(w), 2508(w), 2363(w), 2205(w), 2072(w), 1945(w) 1730(s), 1628(s), 1564(w), 1534(w), 1420(m), 1337(m), 1293(m), 1247(w), 1178(m), 1064(m), 1028(s), 964(w), 928(w), 910(w), 866(m), 837(s), 746(m), 685(m), 668(m), 619(m), 555(m), 534(s), 501(w) (Figure S9).

### Single-crystal X-ray diffraction studies

Details of the crystal structure determination for **1−4**, **1α** and **1β** are presented in the Supplementary Information. The crystal data and refinement details for **1−4**, **1α** and **1β** are summarized in Table 1. The selected bond distances and angles for **1–4**, **1α** and **1β** are given in Tables S1–S6, respectively. The bond valence sums (BVS) were calculated using the VaList program (version 4.0.7)^74^. The calculated BVS values (Table S7) are in good agreement with the expected oxidation states.

**Table 1:** Crystallographic and structure refinement data for compounds **1−4**, **1α** and **1β**.

	1	2	3	4	1α	1β
Formula	C_16_H_20_N_4_O_6_Zn	C_17_H_22_N_4_O_6_Zn	C_17_H_22_N_4_O_7_Zn	C_30_H_34_N_8_O_11_Zn_2_	C_16_H_20_N_4_O_6_Zn	C_47_H_56_N_12_O_17_Zn_3_
Formula weight	429.75	443.78	459.78	813.43	429.73	1257.14
Crystal color, habit	colorless, plate	colorless, plate	colorless, needle	colorless, plate	light yellow, needle	colorless, plate
*T* (K)	298(2)	298(2)	298(2)	298(2)	298(2)	298(2)
*λ* (Å)	0.71073	0.71073	0.71073	0.71073	0.71073	0.71073
Crystal system	monoclinic	monoclinic	orthorhombic	monoclinic	orthorhombic	monoclinic
Space group	*P*2_1_/*n*	*P*2_1_/*n*	*Pcca*	*C*2/*c*	*Pbca*	*C*2/*c*
Crystal size (mm)	0.35 × 0.30 × 0.15	0.30 × 0.25 × 0.15	0.35 × 0.35 × 0.20	0.50 × 0.30 × 0.25	0.45 × 0.10 × 0.10	0.45 × 0.45 × 0.20
*a* (Å)	9.0484(18)	9.0962(3)	19.373(4)	10.884(2)	20.117(4)	22.189(4)
*b* (Å)	22.651(5)	22.830(5)	5.5292(11)	16.879(3)	7.9052(16)	9.3155(19)
*c* (Å)	10.454(2)	10.648(2)	18.208(4)	19.096(4)	23.956(5)	27.691(5)
*β* (°)	112.60(3)	112.13(3)	90	94.84(3)	90	111.25(3)
*V* (Å^3^)	1978.1(8)	2048.3(8)	1950.4(7)	3495.5(12)	3809.7(13)	5335(2)
Z	4	4	4	4	8	4
*D*_calc_(g cm^−1^)	1.443	1.439	1.566	1.546	1.498	1.565
*θ*_min_, *θ*_max_(°)	1.798-25	2.249–24.999	2.102–24.995	2.141–24.996	1.70-24.993	1.969-25
*F*_*000*_	888	920	952	1672	1776	2592
*µ* (mm^−1^)	1.280	1.239	1.307	1.442	1.329	1.420
Index ranges	-7 ≤ *h* ≤ 10	-10 ≤ *h* ≤ 10	-23 ≤ *h* ≤ 20	-12 ≤ *h* ≤ 12	-23 ≤ *h* ≤ 20	-26 ≤ *h* ≤ 26
	-26 ≤ *k* ≤ 26	-27 ≤ *k* ≤ 23	-6 ≤ *k* ≤ 5	-19 ≤ *k* ≤ 20	-9 ≤ *k* ≤ 8	-11 ≤ *k* ≤ 10
	-12 ≤ *l* ≤ 12	-12 ≤ *l* ≤ 12	-21 ≤ *l* ≤ 18	-22 ≤ *l* ≤ 22	-28 ≤ *l* ≤ 25	-27 ≤ *l* ≤ 32
Data collected	9903	10,620	5495	12,971	11,162	12,180
Unique data (*R*_int_)	3468, (0.1711)	3601, (0.2086)	1727, (0.1291)	3072, (0.2181)	3354, (0.1985)	4693, (0.1059)
*R*_*1*_^*a*^, *wR*_*2*_^*b*^(I > 2σ(I))	0.0726, 0.1426	0.0932, 0.2049	0.0558, 0.1078	0.0827, 0.1681	0.0962, 0.2124	0.0676, 0.1565
*R*_*1*_^*a*^, *wR*_*2*_^*b*^ (all data)	0.1394, 0.1623	0.1734, 0.2345	0.1063, 0.1219	0.1736, 0.2160	0.2019, 0.2881	0.1176, 0.1755
GOF on *F*^*2*^ (S)	0.944	0.975	0.984	0.979	0.994	0.922
Largest diff. peak, hole (e Å^−3^)	0.590, -0.470	1.080, -0.615	0.601, -0.273	0.679, -0.478	0.674, -1.613	1.122, -0.915
CCDC No.	2,322,873	2,322,874	2,322,875	2,322,876	2,322,877	2,322,878

^*a*^ $${R}_{1} = \sum {\left| {\left| {F_{\tt{O}} } \right| - \left| {F_{\tt{C}} } \right|} \right|} /\sum {\left| {F_{\tt{O}} } \right|}.$$

^*b*^$${wR}_{2} = \left[ {{{\sum {\left( {w\left({F_{\tt{O}}^{2} - F_{\tt{O}}^{2} } \right)^{2} } \right)} } \mathord{\left/ {\vphantom {{\sum {\left( {w\left( {F_{\tt{O}}^{2} - F_{O}^{2} } \right)^{2} } \right)} } {\sum {\left( {w\left( {F_{\tt{O}}^{2} } \right)^{2} } \right)} }}} \right. \kern-\nulldelimiterspace} {\sum {\left( {w\left({F_{\tt{O}}^{2} } \right)^{2} } \right)} }}} \right]^{{1/2}}$$.

## Results and discussion

### Synthesis

Compounds **1−4**, **1α** and **1β** were prepared by reaction of **4bpu** and Zn(OAc)_2_.2H_2_O using the layering method. Additionally, the normal mixing method (slow evaporation) has been tested as a simpler method but has led to the formation of a precipitate. The amounts used for synthesis are optimal in terms of solubility, and the introduced layering method is reproducible technique. Distinguishingly, **1−4** were obtained in the MeOH, EtOH, ethylene glycol and propylene glycol solvents, respectively. Similar to the synthesis method of compound **1**, using individually 3-nitrophenol and 1,3-dinitrobenzene as additive reagent, compounds **1α** and **1β** were acquired, respectively. These additives were also investigated in alternative solvents, but they yielded the same compounds **2–4**. To further investigate the effect of the additive, other components such as 4-nitrophenol, 4-nitrochlorobenzene, 2,4-dinitrochlorobenzene, 2-aminophenol, 3-aminophenol and 4-aminophenol were used in MeOH, EtOH, ethylene glycol and propylene glycol solvents. The results of the investigation of the unit cell dimensions of the single crystals formed by using these additives showed that none of them led to the formation of a new compound. The selected additives possess suitable sites for acting as hydrogen bond donors or acceptors. Additionally, previous studies have shown that compounds containing urea group are highly effective in the detection of nitroaromatic compounds^19^. Therefore, in this work, we aimed to investigate the role of these compounds in the formation of single crystals and in promoting structural diversity.

### Crystal structure description of compounds 1−4, 1α and 1β

#### Compound {[Zn(4bpu)(OAc)_2_](CH_3_OH)}_n_ (1)

X-ray crystallography for the single crystals of compound **1** shows that it crystallizes in the monoclinic crystal system and *P*2_1_/*n* space group. Its asymmetric unit consists of one [Zn(4bpu)(OAc)_2_] unit and one MeOH molecule as guest.

**Fig. 1:**
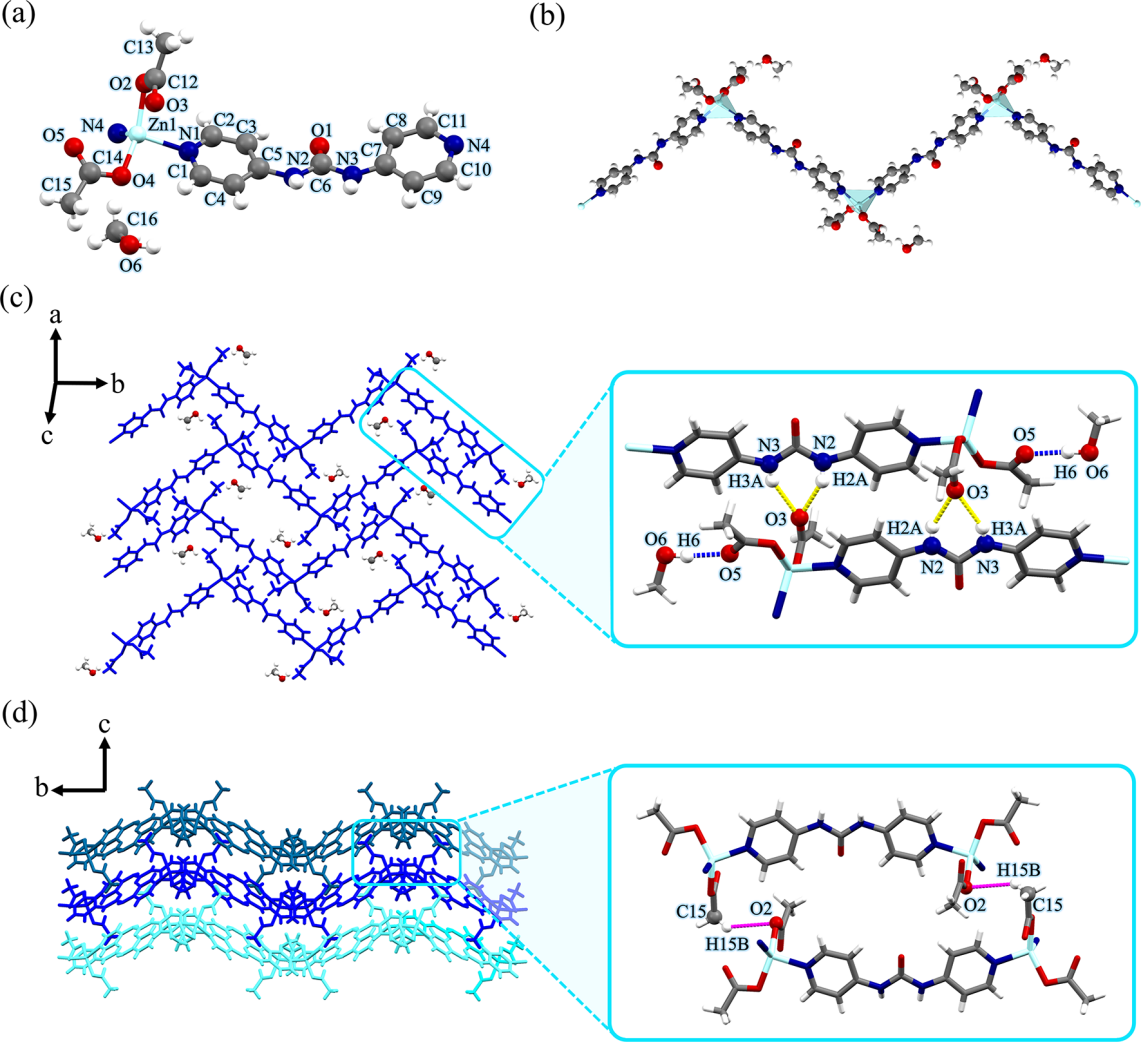
(**a**) View of the coordination environment around zinc(II) with atom labeling scheme in compound **1**. Color code: Zn, light blue; O, red; N, blue; C, grey. (**b**) A section of the 1D zig-zag chain structure of compound **1**. Metal polyhedrons are in light blue for Zn coordination environment. (**c**) The 2D supramolecular layer in compound **1** assembled by hydrogen bond interactions. These interactions are represented by yellow dashed lines. Blue dashed lines denote hydrogen bonds between MeOH molecules and acetate anions. (**d**) The 3D structure of compound **1** formed by hydrogen bonds represented by magenta dashed lines along *c*-axis. For clarity, MeOH molecules were omitted.

As depicted in Fig. 1a, each Zn(II) center shows a four-coordinate environment, wherein two sites were occupied by the pyridyl nitrogen atoms of two different **4bpu** ligands, and the other two positions were completed by two oxygen atoms of acetate anions (unidentate coordination mode). The Zn−O/N bond lengths (Table S1) are all located in the normal accepted range of Zn(II) compounds^75–77^. The geometry of the four-coordinate metal centers can be described by an angular index (*τ*_4_) parameter, which was defined by Houser and co-workers as $\left[ {\left( {360^{^\circ } - \left( {a + \beta } \right)} \right)/141^{^\circ } } \right]$, which *α* and *β* are the two largest angles around a four-coordinate metal center. A *τ*_4_ value of 0 would correspond to a square planar geometry, *τ*_4_ of 0.07, 0.18, 0.5, 0.64 to a seesaw geometry, *τ*_4_ of 0.85 to a trigonal pyramidal geometry, and *τ*_4_ of 1 to a tetrahedral geometry^78^. The central Zn(II) ion displaying a distorted {ZnN_2_O_2_} trigonal pyramidal geometry with the *τ*_4_ parameter being 0.76^78^. In compound **1**, each **4bpu** links two Zn(II) centers to afford a Zn_2_L unit that propagates into a one-dimensional (1D) zig-zag chain in which the distance of two adjacent Zn(II) ions is 14.112 Å (Fig. 1b). Also, the Zn⋅⋅⋅Zn distance in zig-zag chain period and Zn⋅⋅⋅Zn⋅⋅⋅Zn angle is 22.651 Å and 106.75°, respectively (Figure S10 and Table 2).

The typical bifurcated hydrogen bonding interactions of urea moieties (interaction between the carbonyl O atom and the two N−H of the adjacent urea groups) that lead to the formation of 1D hydrogen bonded tape motif was absent in the structure^22,23^. Instead, the 1D zig-zag chains were found to self-assemble into a two-dimensional (2D) network by hydrogen bonding interactions of urea groups with acetate anions and forming $\:{\text{R}}_{\text{2}}^{\text{1}}\text{(6)}$ graph set motif^79,80^. The acetate anions acting as bifurcated acceptors N2−H2A⋯O3 [D⋯A distance = 2.768(7) Å and D−H⋯A angle = 146(17)°] and N3−H3A⋯O3 [D⋯A distance = 2.850(7) Å and D–H⋯A angle = 157(15)°] and also the second acetate anion creates the D(2) graph set by methanol−O−H⋯O (acetate) [D⋯A distance = 2.716(11) Å and D−H⋯A angle = 174(15)°] hydrogen bond (Fig. 1c). As shown in Fig. 1d, the 2D layers further stack along the *a*-axis through the interlayer acetate−C−H⋯O (acetate) [D⋯A distance = 3.711 Å and D−H⋯A angle = 139.93°] interactions to form the three-dimensional (3D) supramolecular structure. A more detailed geometrical description of the hydrogen bond interactions that were presented in Fig. 1c and d is provided in Table S8.

In all compounds, the color scheme for the hydrogen bonding interactions leading to the formation of 2D layers has been selected as follows: interactions between solvents and other sites (blue dashed lines for methanol, orange dashed lines for ethanol, green dashed lines for ethylene glycol, and purple dashed lines for H_2_O), and interactions involving the acetate are represented by yellow dashed lines. The intermolecular interactions leading to the formation of the 3D structure are shown with magenta dashed lines.

#### Compound {[Zn(4bpu)(OAc)_2_](C_2_H_5_OH)}_n_ (2)

Single-crystal X-ray diffraction analysis revealed that **2** crystallized in the monoclinic crystal system and *P*2_1_/*n* space group. Its asymmetric unit comprises one [Zn(4bpu)(OAc)_2_] unit and one guest EtOH molecule. Compounds **1** and **2** are highly isostructural; the unit cell dimensions are similar (Table 1) and the disposition of atoms is almost identical (with exchange MeOH↔EtOH). In principle, these compounds are isostructural pseudopolymorphs.

**Fig. 2:**
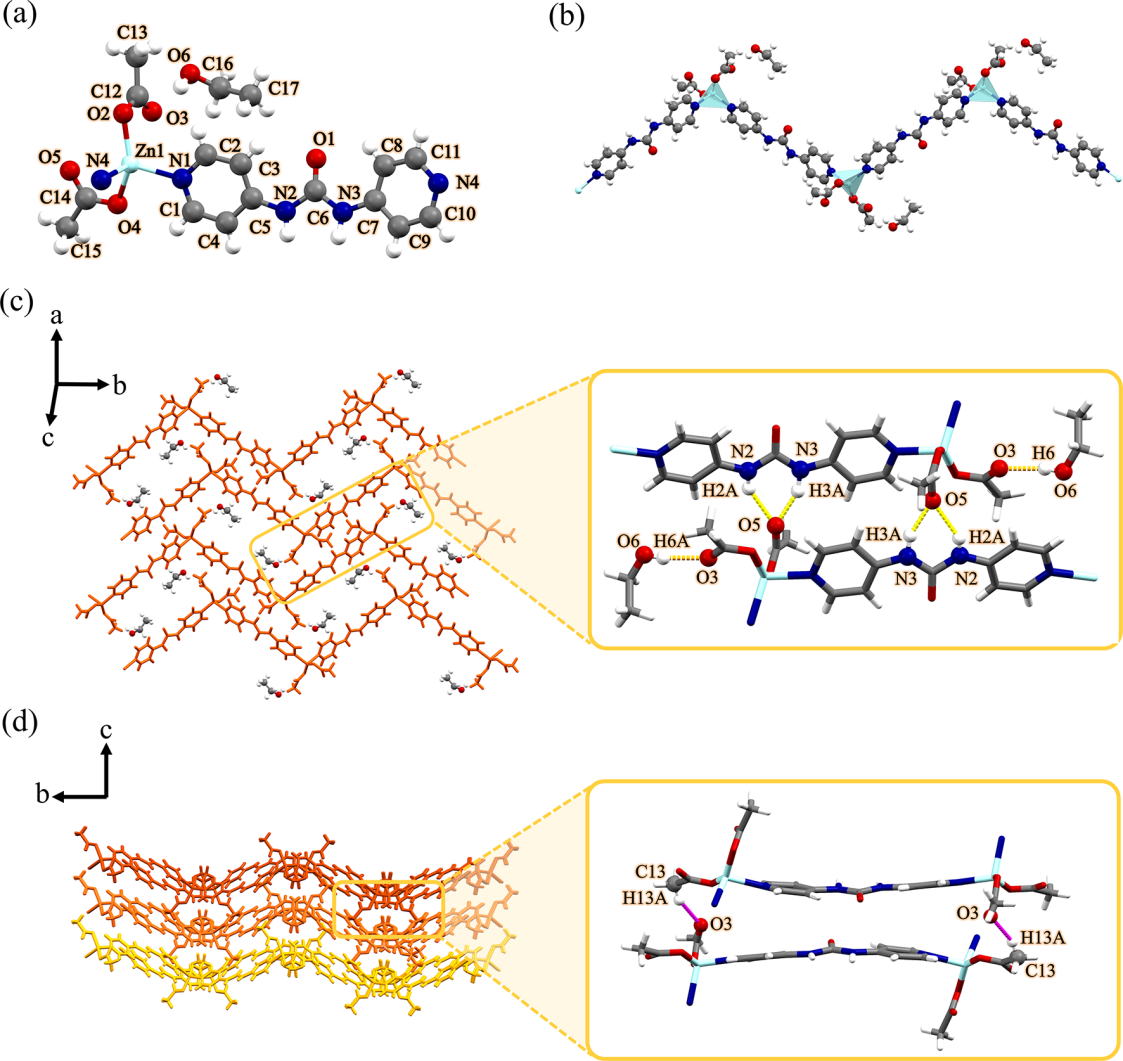
(**a**) View of the coordination environment around zinc(II) with atom labeling scheme in compound **2**. Color code: Zn, light blue; O, red; N, blue; C, grey. (**b**) A section of the 1D zig-zag chain of compound **2**. Metal polyhedrons are in light blue for Zn coordination environment. (**c**) The 2D supramolecular layer in compound **2** assembled by hydrogen bond interactions. These interactions are represented by yellow dashed lines. Orange dashed lines denote hydrogen bonds between EtOH molecules and acetate anions. (**d**) The 3D structure of compound **2** formed by hydrogen bonds represented by magenta dashed lines along *c*-axis. For clarity, EtOH molecules were omitted.

As illustrated in Fig. 2a, each Zn(II) center shows a four-coordinate environment, wherein two sites were occupied by the pyridyl nitrogen atoms of two different **4bpu** ligands, and the other two positions were completed by two oxygen atoms of acetate anions (unidentate coordination mode). Both Zn–N and Zn–O bond distances are well matched with those observed in similar compounds^75–77^. Selected bond distances and angles for compound **2** are listed in Table S2. The central Zn(II) ion displaying a distorted {ZnN_2_O_2_} trigonal pyramidal geometry with the *τ*_4_ parameter being 0.74^78^. In compound **2**, **4bpu** ligands bridge the subsequent Zn(II) centers to afford a Zn_2_L unit and this gives rise to the 1D zig-zag chain in which the distance of two adjacent Zn(II) ions is 14.115 Å (Fig. 2b). Distance of Zn⋅⋅⋅Zn in the zig-zag chain period and Zn⋅⋅⋅Zn⋅⋅⋅Zn angle in compound **2** is 22.830 Å and 107.94°, respectively (Figure S11 and Table 2). The proximity of these values in compounds **1** and **2** confirms the isostructural relationship of these two compounds.

Instead of typical α-tape motif^22,23^, the 1D zig-zag chains are interconnected by hydrogen bonding interactions of urea groups with acetate anions forming $\:{\text{R}}_{\text{2}}^{\text{1}}\text{(6)}$ graph set notation^79,80^ and form 2D network. The acetate anions acting as bifurcated acceptors N2−H2A⋯O5 [D⋯A distance = 2.869(9) Å and D−H⋯A angle = 148(19)°] and N3−H3A⋯O5 [D⋯A distance = 2.750(10) Å and D−H⋯A angle = 155(18)°]. Also, acetate anion forms hydrogen bonding which creates the D(2) graph set by ethanol−O−H⋯O (acetate) [D⋯A distance = 2.745(17) Å and D−H⋯A angle = 170.3°] (Fig. 2c). As shown in Fig. 2d, the 2D layers further stack through the interlayer acetate−C−H⋯O (acetate) [D⋯A distance = 3.538 Å and D−H⋯A angle = 148.67°] interactions to form the 3D supramolecular structure. A more detailed of geometrical description of the hydrogen bond interactions that were presented in Fig. 2c and d is listed in Table S9.

#### Compound {[Zn(4bpu)(OAc)_2_](HOCH_2_CH_2_OH)}_n_ (3)

Single-crystal X-ray diffraction analysis indicates that crystals of compound **3** conform to the orthorhombic *Pcca* space group and asymmetric unit contains of one [Zn_0.5_(4bpu)_0.5_(OAc)] unit and half of guest ethylene glycol molecule.

**Fig. 3:**
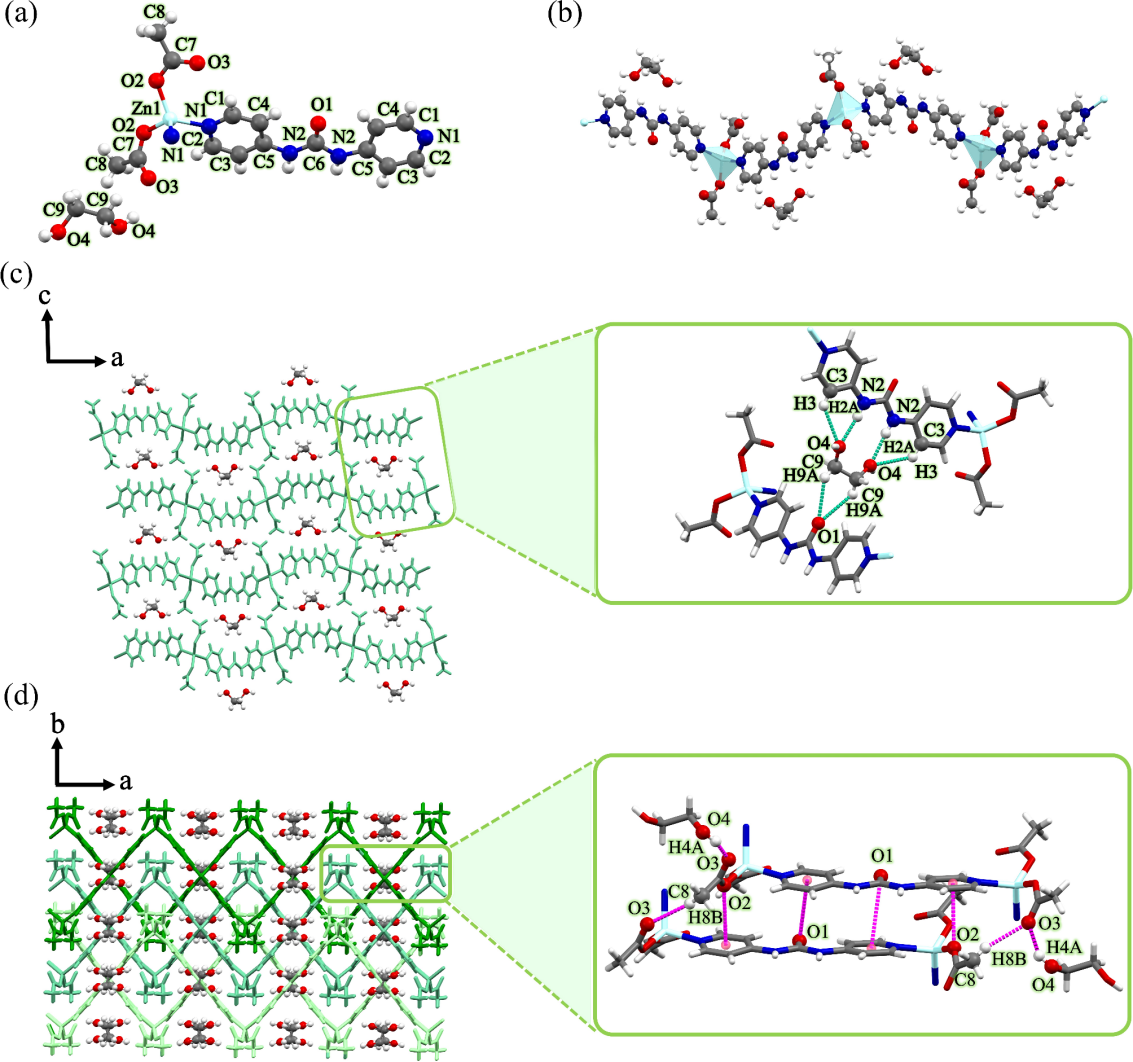
(**a**) View of the coordination environment around zinc(II) with atom labeling scheme in compound **3**. Color code: Zn, light blue; O, red; N, blue; C, grey. (**b**) A section of the 1D zig-zag chain of compound **3**. Metal polyhedrons are in light blue for Zn coordination environment. (**c**) The 2D supramolecular layer in compound **3** assembled by hydrogen bond interactions. These interactions are represented by green dashed lines. (**d**) The 3D structure of compound **3** formed by supramolecular lone pair–π interactions and hydrogen bonds represented by magenta dashed lines along *a*-axis.

As illustrated in Fig. 3a, each Zn(II) center shows a four-coordinate environment, wherein two sites were occupied by the pyridyl nitrogen atoms of two different **4bpu** ligands, and the other two positions were completed by two oxygen atoms of acetate anions (unidentate coordination mode). The Zn−O/N bond lengths (Table S3) are all located in the normal accepted range of Zn(II) compounds^75–77^. The central Zn(II) ion displaying distorted {ZnN_2_O_2_} trigonal pyramidal geometry, with the *τ*_4_ parameter being 0.83^78^. As shown in Fig. 3b, each **4bpu** ligands in compound **3** bridge the subsequent Zn(II) ions to form a 1D zig-zag chain. The distance between the adjacent Zn(II) ions and the distance of Zn⋅⋅⋅Zn in the zig-zag chain period is 14.014 and 19.373 Å, respectively. Also, Zn⋅⋅⋅Zn⋅⋅⋅Zn angle in compound **3** is 87.45° (Figure S11 and Table 2).

Furthermore, these one-dimensional chains sustained on the hydrogen bond with ethylene glycol molecules to form 2D supramolecular layer (Fig. 3c). The ethylene glycol molecules acting as bifurcated hydrogen bond donor and acceptor in the two-dimensional supramolecular layer *via* two $\:{\text{R}}_{\text{2}}^{\text{1}}\text{(6)}$ and one $\:{\text{R}}_{\text{2}}^{\text{1}}\text{(5)}$ graph set notation^79,80^, N2−H2A⋯O4 [D⋯A distance = 2.889(6) Å and D−H⋯A angle = 170(4)°], C3−H3⋯O4 [D⋯A distance = 3.276(7) Å and D–H⋯A angle = 136.3°], C9−H9A⋯O1 [D⋯A distance = 3.314 Å and D–H⋯A angle = 127.02°]. The 2D layers further stack along the *a*-axis through the interlayer acetate−C−H⋯O (acetate) [D⋯A distance = 3.502(8) Å and D−H⋯A angle = 159.7°] and ethylene glycol−O−H⋯O (acetate) [D⋯A distance = 2.728(6) Å and D−H⋯A angle = 141(7)°] interactions to form the three-dimensional supramolecular structure (Fig. 3d). A more detailed description of the hydrogen bond interactions that were presented in Fig. 3c and d is provided in Table S9.

Interestingly, lone pair–π interactions are observed between carbonyl and pyridine moiety of adjacent **4bpu** ligands and also between acetate anions and pyridine ring of **4bpu** ligand with carbonyl⋯Cg distance of 3.760 and 3.391 Å, respectively. These lone pair–π interactions can stabilize the structure efficiently (Fig. 3d).

#### Compound {[Zn(4bpu)(OAc)_2_](0.5H_2_O)}_n_ (4)

X-ray crystallography for the single crystals of compound **4** shows that it crystallizes in the monoclinic crystal system and *C*2/*c* space group. In this compound, asymmetric unit consists of one [Zn(4bpu)(OAc)_2_] unit and half of one guest H_2_O molecule.

**Fig. 4:**
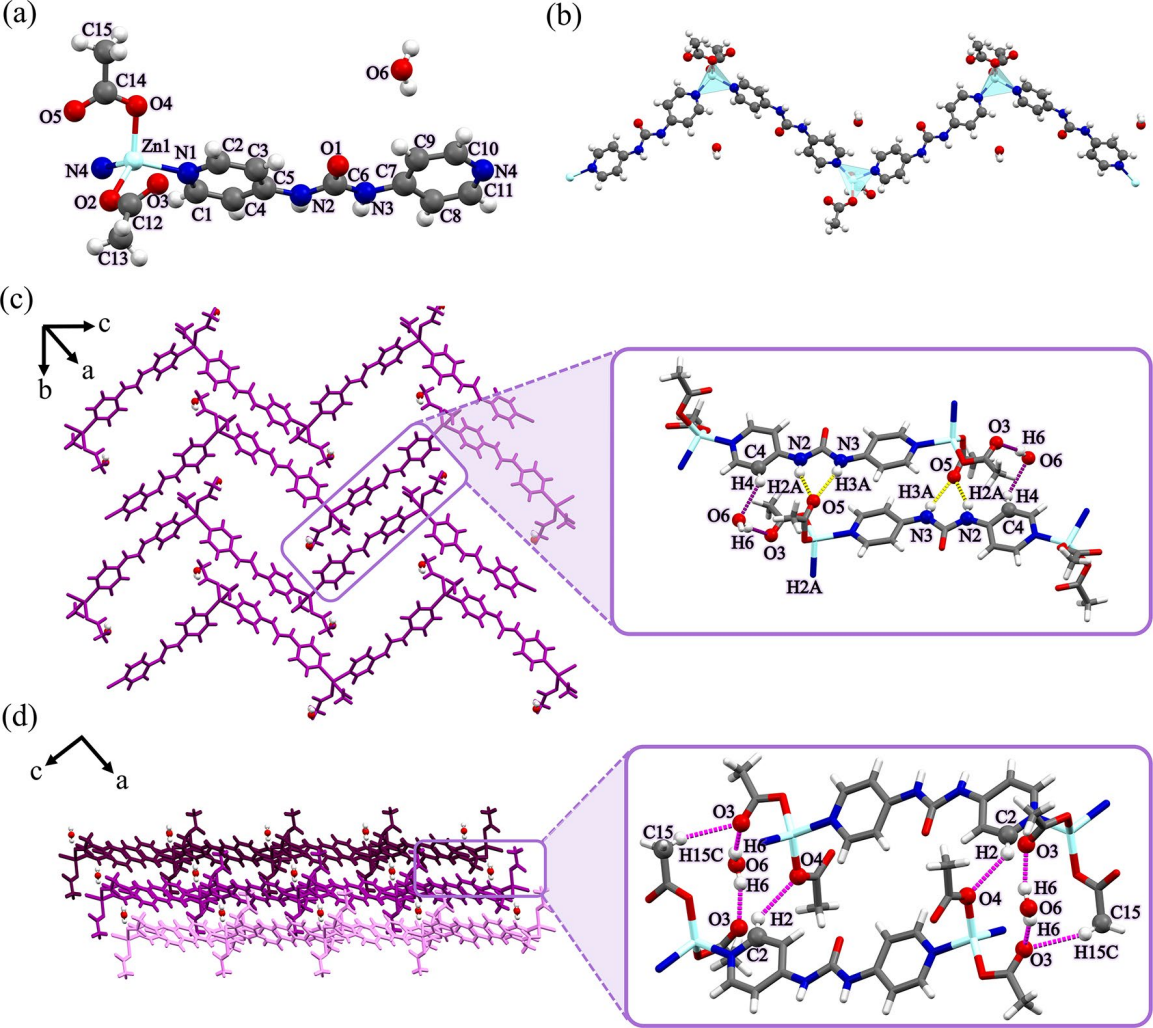
(**a**) View of the coordination environment around zinc(II) with atom labeling scheme in compound **4**. Color code: Zn, light blue; O, red; N, blue; C, grey. (**b**) A section of the 1D zig-zag chain of compound **4**. Metal polyhedrons are in light blue for Zn coordination environment. (**c**) The 2D supramolecular layer in compound **4** assembled by hydrogen bond interactions. These interactions are represented by yellow dashed lines. Purple dashed lines denote hydrogen bonds between H_2_O molecules and framework. (**d**) The 3D structure of compound **4** formed by hydrogen bonds represented by magenta dashed lines.

As shown in Fig. 4a, each Zn(II) center shows a four-coordinate environment, wherein two sites were occupied by the pyridyl nitrogen atoms of two different **4bpu** ligands, and the other two positions were completed by two oxygen atoms of acetate anions (unidentate coordination mode) in the normal range of Zn–N and Zn–O distances^75–77^. Selected bond distances and angles for compound **4** are listed in Table S4. The central Zn(II) ion displaying a {ZnN_2_O_2_} trigonal pyramidal geometry with the *τ*_4_ parameter being 0.85^78^. As depicted in Fig. 4b, each **4bpu** ligand links the neighboring Zn(II) centers to afford a Zn_2_L unit and generates an infinite 1D zig-zag chain. The distance between the adjacent Zn(II) ions and the distance of Zn⋅⋅⋅Zn in the zig-zag chain period is 14.152 and 22.764 Å, respectively. In this structure, the Zn⋅⋅⋅Zn⋅⋅⋅Zn angle is 107.08° (Figure S11 and Table 2).

Instead of formation of typical α-tape motif in urea containing ligands^22,23^, the 1D chains are connected to form two-dimensional network through hydrogen bonding interactions of urea groups with acetate anions and forming cyclic $\:{\text{R}}_{\text{2}}^{\text{1}}\text{(6)}$ graph set motif. The acetate anions act as bifurcated hydrogen bond acceptors: N2−H2A⋯O5 [D⋯A distance = 2.797(10) Å and D−H⋯A angle = 154(11)°] and N3−H3A⋯O5 [D⋯A distance = 2.873(10) Å and D–H⋯A angle = 148(11)°]. Furthermore, the 1D chains are linked by H_2_O molecules through water−O6−H6⋯O3 (acetate) [D⋯A distance = 2.787(13) Å and D–H⋯A angle = 166(14)°] and pyridine−C4−H4⋯O6 (water) [D⋯A distance = 3.165(12) Å and D–H⋯A angle = 137 °] hydrogen bonding interactions (Fig. 4c). As depicted in Fig. 4d, the 2D layers further stack through the interlayer water−O6−H6⋯O3 (acetate) [D⋯A distance = 2.787(13) Å and D−H⋯A angle = 166(14)°], water−O6−H6⋯O3 (acetate) [D⋯A distance = 2.787(13) Å and D−H⋯A angle = 166(14)°], acetate−C15− H15C⋯O3 (acetate) [D⋯A distance = 3.504 Å and D−H⋯A angle = 145.07°] and pyridine−C2−H2⋯O4 (acetate) [D⋯A distance = 3.240(11)  Å and D−H⋯A angle = 134.9°] interactions to form the 3D supramolecular structure (Fig. 4c and d and Table S9).

#### Compound {[Zn(4bpu)(OAc)_2_](CH_3_OH)}_n_ (1α)

Single-crystal X-ray diffraction analysis revealed that **1α** crystallized in the orthorhombic crystal system and *Pbca* space group. Its asymmetric unit comprises of one [Zn(4bpu)(OAc)_2_] unit and one MeOH solvent molecule.

**Fig. 5:**
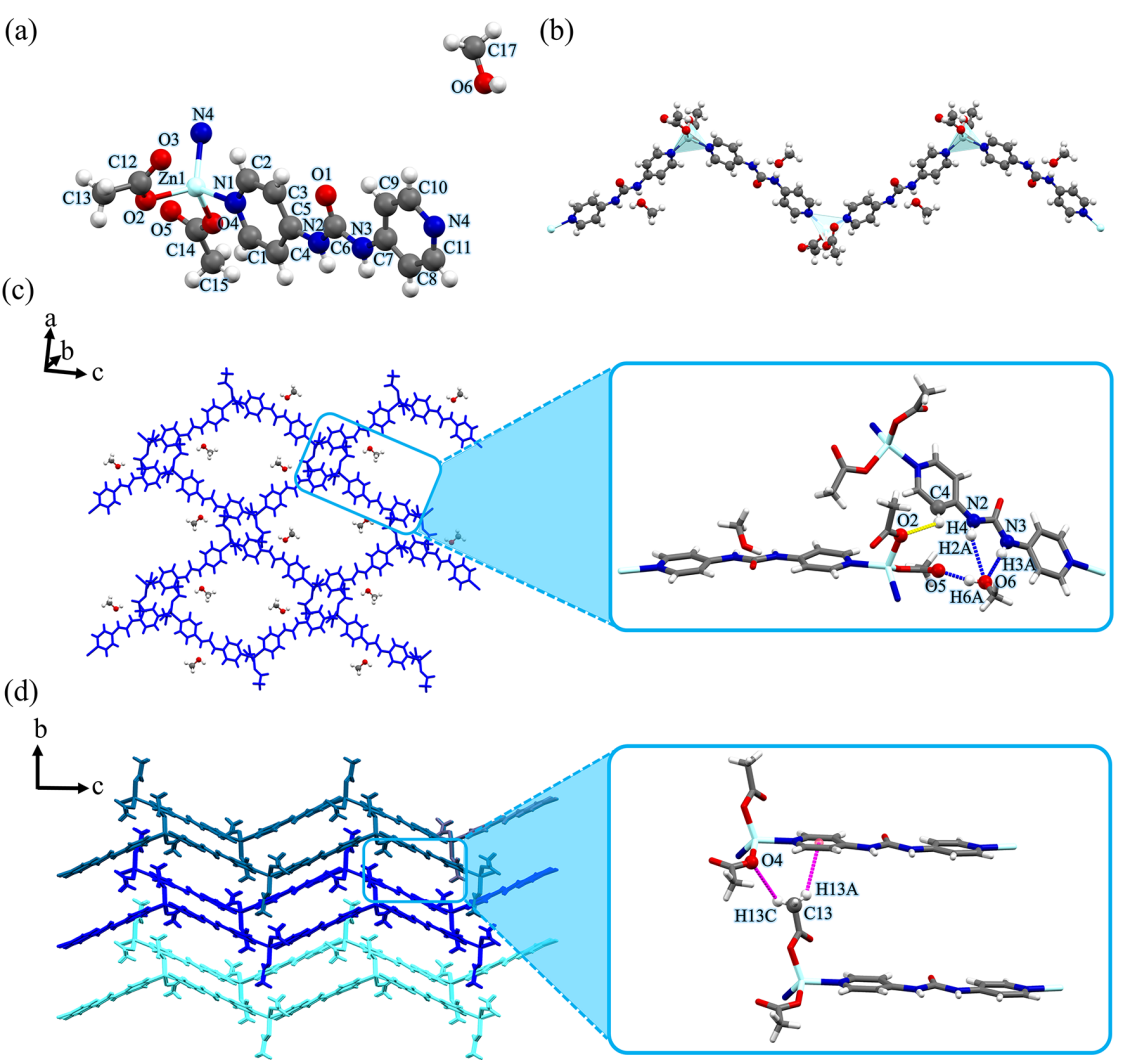
(**a**) View of the coordination environment around zinc(II) with atom labeling scheme in compound **1α**. Color code: Zn, light blue; O, red; N, blue; C, grey. (**b**) A section of the 1D chain of compound **1α**. Metal polyhedrons are in light blue for Zn coordination environment. (**c**) The 2D supramolecular layer in compound **1α** assembled by hydrogen bond interactions. These interactions are represented by yellow dashed lines. Blue dashed lines denote hydrogen bonds between MeOH molecules and framework. (**d**) The 3D structure of compound **1α** formed by supramolecular interactions represented by magenta dashed lines along *b*-axis. For clarity, MeOH molecules were omitted.

As shown in Fig. 5a, each Zn(II) center shows a four-coordinate environment, wherein two sites were occupied by the pyridyl nitrogen atoms of two different **4bpu** ligands, and the other two positions were completed by two oxygen atoms of acetate anions (unidentate coordination mode). The Zn−O/N bond lengths are all located in the normal accepted range of Zn(II) compounds (Table S5)^75–77^. The central Zn(II) ion displaying a distorted {ZnN_2_O_2_} trigonal pyramidal geometry with the *τ*_4_ parameter being 0.92^78^. In compound **1α**, each **4bpu** ligands bridge the subsequent zinc metal centers to afford a Zn_2_L unit and this gives rise to the 1D zig-zag chain in which the distance of two adjacent zinc ions is 14.275 Å (Fig. 5b). Also, distance of Zn⋅⋅⋅Zn in the zig-zag chain period and Zn⋅⋅⋅Zn⋅⋅⋅Zn angle is 23.956 Å and 114.09°, respectively (Figure S10 and Table 2).

In this compound, 1D chains are further self-assembled *via* hydrogen bonding interactions: N2−H2A⋯O6 [D⋯A distance = 3.327(19) Å and D−H⋯A angle = 145.2°], N3−H3A⋯O6 [D⋯A distance = 2.809(18) Å and D−H⋯A angle = 172.5°], O6−H6A⋯O5 [D⋯A distance = 2.613(15) Å and D−H⋯A angle = 167.2(2)°] and C4−H4⋯O2 [D⋯A distance = 3.279 Å and D−H⋯A angle = 119.78°], wherein the MeOH molecule acts as the hydrogen bonding bridge between urea moiety and acetate oxygen atom (Fig. 5c). As depicted in Fig. 5d, the 2D layers further stack through the interlayer acetate−C13−H13C⋯O4 (acetate) [D⋯A distance = 3.655 Å and D−H⋯A angle = 110.80°] hydrogen bonding and *via* acetate−C13−H13A⋯π (pyridine ring) form the 3D supramolecular structure (Fig. 5c and d and Table S8).

**Table 2:** Geometric characteristics for zig-zag chains of **1–4** and **1α**.

Compounds	1	2	3	4	1α
M1⋅⋅⋅M2, d [Å]	14.112	14.115	14.014	14.152	14.275
M2⋅⋅⋅M3, d [Å]	14.112	14.115	14.014	14.152	14.275
M1⋅⋅⋅M3 (Length of the zig-zag chain period), d [Å]	22.651	22.830	19.373	22.764	23.956
M1⋅⋅⋅M2⋅⋅⋅M3 angle, α [°]	106.75	107.94	87.45	107.08	114.09
*τ*_4_	0.76	0.74	0.83	0.85	0.92
Geometry	Distorted trigonal pyramidal	Distorted trigonal pyramidal	Distorted trigonal pyramidal	Trigonal pyramidal	Distorted trigonal pyramidal

#### Compound {[Zn_3_(4bpu)_3_(OAc)_6_](CH_3_OH)_2_}_n_ (1β)

The single-crystal X-ray analysis for compound **1β** reveals that it crystallizes in the monoclinic crystal system and *C*2/*c* space group. Its asymmetric unit comprises one [Zn_1.5_(4bpu)_1.5_(OAc)_3_] unit and one MeOH guest molecule.

**Fig. 6:**
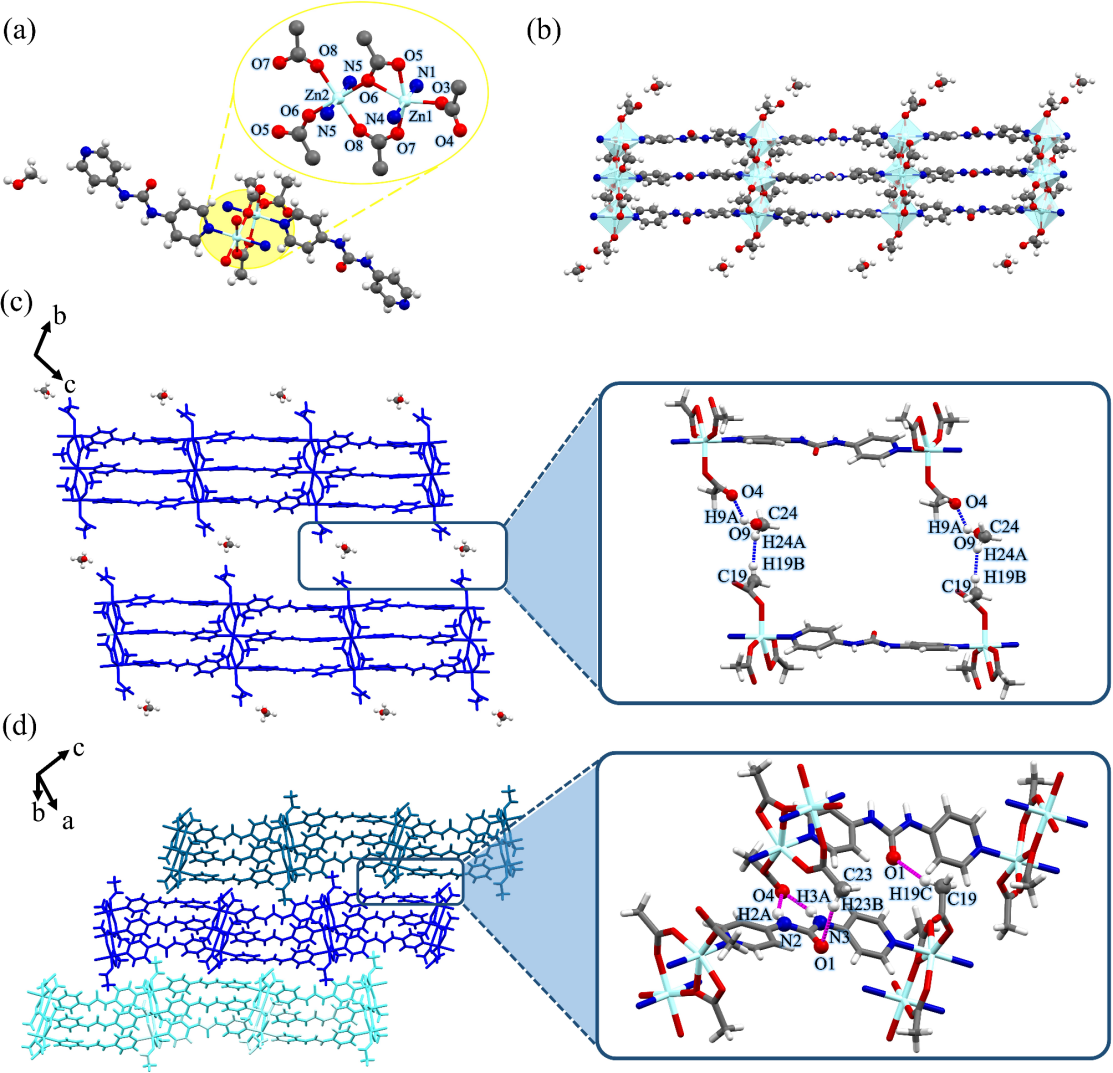
(**a**) View of the coordination environment around zinc(II) ions with atom labeling scheme in compound **1β**. Color code: Zn, light blue; O, red; N, blue; C, grey. (**b**) A section of the 1D triple-stranded ladder of compound **1β**. Metal polyhedrons are in light blue for Zn coordination environment. (**c**) The 2D supramolecular layer in compound **1β** assembled by hydrogen bond interactions. Blue dashed lines denote hydrogen bonds between MeOH molecules and framework. (**d**) The 3D structure of compound **1β** formed by hydrogen bonds represented by magenta dashed lines along *b*-axis. For clarity, MeOH molecules were omitted.

In compound **1β** acetate anions participate in the self-assembly process along with **4bpu** ligands and form a rare 1D triple-stranded ladder structural motif^81–85^. In this compound, there are three different coordination modes for acetate anions: monodentate, bridge and chelate-bridge modes. As shown in Fig. 6a, the Zn1 and Zn2 centers are linked by acetate anions to form Zn_2_ units. As depicted in Figure S12, distance between Zn1 and Zn2 in Zn_2_ unit is 3.863 Å. As seen in Fig. 6b and S12, Zn1 atoms are located on the outer legs and are six-coordinated by two pyridine nitrogen atoms of two different **4bpu** ligands and four oxygen atoms of three acetate anions that form a {ZnN_2_O_4_} distorted octahedral coordination environment. In the outer legs, the distance between the adjacent Zn(II) ions is 14.270 Å and the Zn⋅⋅⋅Zn⋅⋅⋅Zn angle is 176.42°. Also, the Zn2 atom is located on the middle leg and metal center is six-coordinated by two pyridine nitrogen atoms of two different **4bpu** ligands and four oxygen atoms of four acetate anions that form a {ZnN_2_O_4_} distorted octahedral coordination geometry. In the middle leg, the distance between the adjacent Zn(II) ions and the Zn⋅⋅⋅Zn⋅⋅⋅Zn angle are 14.263 Å and 180°, respectively. Both Zn–N and Zn–O bond distances are well matched with those observed in similar compounds^75,76^. Selected bond distances and angles for compound **1β** are listed in Table S6.

The 1D triple-stranded ladders are further supported by hydrogen bonding interactions of acetate anions and MeOH, to form two-dimensional network. The acetate anions act as hydrogen bond acceptors through O9−H9A⋯O4 hydrogen bonding: [D⋯A distance = 2.450(11) Å and D−H⋯A angle = 133.8°]. Furthermore, the other side of MeOH molecules involves acetate−C19−H19B⋯H24A (MeOH) connection, facilitating interactions among 1D triple stranded-ladder (Fig. 6c). The 2D layers further stack through the interlayer acetate−C23−H23B⋯O1 (urea) [D⋯A distance = 3.713(8) Å and D−H⋯A angle = 148.66°] and acetate−C19− H19⋯O1 (urea) [D⋯A distance = 3.417(11) Å and D−H⋯A angle = 148.7°]. Furthermore, the acetate anions act as bifurcated acceptors: N2−H2A⋯O4 [D⋯A distance = 3.126(8) Å and D−H⋯A angle = 151.1°] and N3−H3A⋯O4 [D⋯A distance = 2.826(8) Å and D–H⋯A angle = 165.1°] to form the 3D supramolecular structure (Fig. 6d). A more detailed description of the hydrogen bond interactions that were presented in Fig. 6c and d is provided in Table S8.

### The effect of solvent and additive on conformation of ligand

**Fig. 7:**
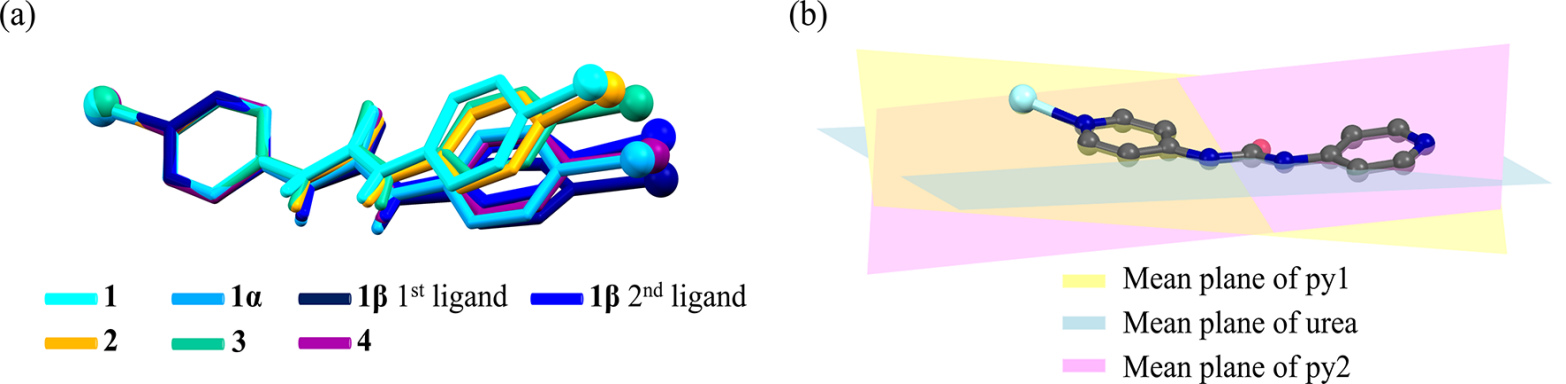
(**a**) Structural overlay image of ligands in **1–4**, **1α** and **1β**. Metal ions are shown as balls. For clarity, hydrogen atoms of pyridyl groups were omitted (**b**) Representation of pyridyl and urea group mean plane in **4bpu** ligand. Metal ion is shown as light blue ball. For clarity, hydrogen atoms were omitted.

The final structure of coordination polymers can be affected by the solvent system used in the synthesis^30,48^. In addition, additives can tune the conformation of the ligand and create structural diversity through structural isomerism^28^. By comparing the overlay images in the synthesized supramolecular isomers, significant differences in the conformation, curvature and dihedral angles of the ligands are revealed. Observing these conformational changes in ligands helps to better understand the effects of solvents or additives in the control and structural directing of coordination polymers (Fig. 7a). The molecular conformation of the **4bpu** ligand is considered through angles involving pyridyl-urea and pyridyl-pyridyl planes (Fig. 7b). Investigations into the obtained compounds indicate that the **4bpu** ligands displayed slightly nonplanar conformations (Table 3 and Figures S13–S18).

**Table 3:** Angles between pyridyl-urea and pyridyl-pyridyl planes of ligand in compounds **1−4**, **1α** and **1β**.

	∠ Mean plane of py1−urea [°]	∠ Mean plane of urea−py2 [°]	∠ Mean plane of py1−py2 [°]
Ligand in **1**	12.54	16.17	12.39
Ligand in **2**	17.17	12.57	13.98
Ligand in **3**	4.45	4.45	8.87
Ligand in **4**	6.78	10.75	12.42
Ligand in **1α**	5.93	6.27	2.49
1st ligand in **1β**	9.11	18.20	26.22
2nd ligand in **1β**	12.81	12.81	24.57

### Effect of solvent on controlling pseudopolymorphism in 1−4

Compounds **1−4** show supramolecular isomerism by solvation. These pseudopolymorphic compounds adopt 1D zig-zag chain structure. All solvents in preparation of compounds were used directly without purification and drying procedures. The use of various alcoholic solvents such as methanol, ethanol and ethylene glycol lead to the formation of various pseudopolymorphs **1−3** with different guest solvents. Notably, when propylene glycol is used as solvent in the synthesis of compound **4**, this solvent does not participate in the structure; instead, water serves as the guest solvent in the crystal structure of pseudopolymorph **4** (Scheme 1). In view of our previous work, lattice occluded solvent molecules have significant effects on guest uptake studies via preventing the self-association of urea groups^86^.

### Effect of additive on controlling supramolecular isomerism and morphology of compounds 1, 1α and 1β

Additive molecules can have a crucial role in the construction of supramolecular isomers. There are a few examples of conformational isomers where different additives significantly affect the conformation of the ligands as well as the overall topology of structures^28,58^. However, additive-induced polymorphism of coordination polymers has been rarely reported so far^57^.

According to our experimental procedures and Scheme S1, the polymorphs **1α**, **1β** and **1** were prepared from Zn(OAc)_2_.2H_2_O and **4bpu** ligand in the presence of 3-nitrophenol, 1,3-dinitrobenzene and without additive at room temperature, respectively. Both compounds **1** and **1α** possess the same formula, differing only in their supramolecular interactions with each other and they exhibit 1D zig-zag chain structure. By altering the additive, compound **1β** is formed, characterized by a 1D triple-stranded ladder structural motif. Interestingly, although these additives do not participate in the structural frameworks, we cannot obtain the product **1α** and **1β** without the addition of additives.

**Fig. 8:**
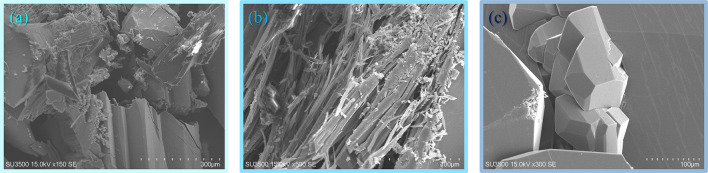
SEM images of (**a**) **1**, (**b**) **1α** and (**c**) **1β**.

Since material properties such as gas storage, catalytic activity, etc. can be affected by crystal size and morphology, recently the control of crystal morphology during the crystallization process has attracted much attention, especially in industry^87–89^. Kitagawa and co-workers were able to increase the catalytic activity of a MOF by controlling its crystal size^87^. As stated in the previous sections, the experimental conditions for the synthesis of **1α** and **1β** are the same. In such conditions, choosing 3-nitrophenol as an additive led to the formation of light-yellow needle-shaped crystals of **1α**, and choosing 1,3-dinitrobenzene led to colorless plate-shaped crystals of **1β**. Additive molecules were apparently able to act as templates and through interactions cause an increase or decrease the growth rate of different face of crystals. For further investigation, SEM images were taken which clearly show the different morphology of **1**, **1α** and **1β** (Fig. 8).

According to Table 4, all compounds were synthesized using the layering method in the presence of various additive agents. Single-crystal structural analysis revealed that, in other cases, the additive had no effect and the compound **1** were obtained. It should be noted that, the impact of additive was noticeable solely when methanol was used and it did not appear with alternative solvents. As shown in Figures S19–S22, the examination of ATR-FTIR spectra further validates these results.

**Table 4:**
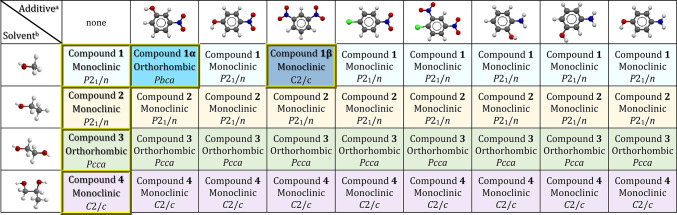
Presentation of the results for additives and solvents used in the synthesis of **1−4**, **1α** and **1β**.

^a^Additive (left to right): 3-nitrophenol, 4-nitrophenol, 1,3-dinitrobenzene, 4-nitrochlorobenzene, 2,4-dinitrochlorobenzene, 2-aminophenol, 3-aminophenol and 4-aminophenol. ^b^Solvent (up to down): methanol, ethanol, ethylene glycol and propylene glycol.

### Dissolution-recrystallization structural transformation (DRST) study in pseudopolymorphs

**Fig. 9:**
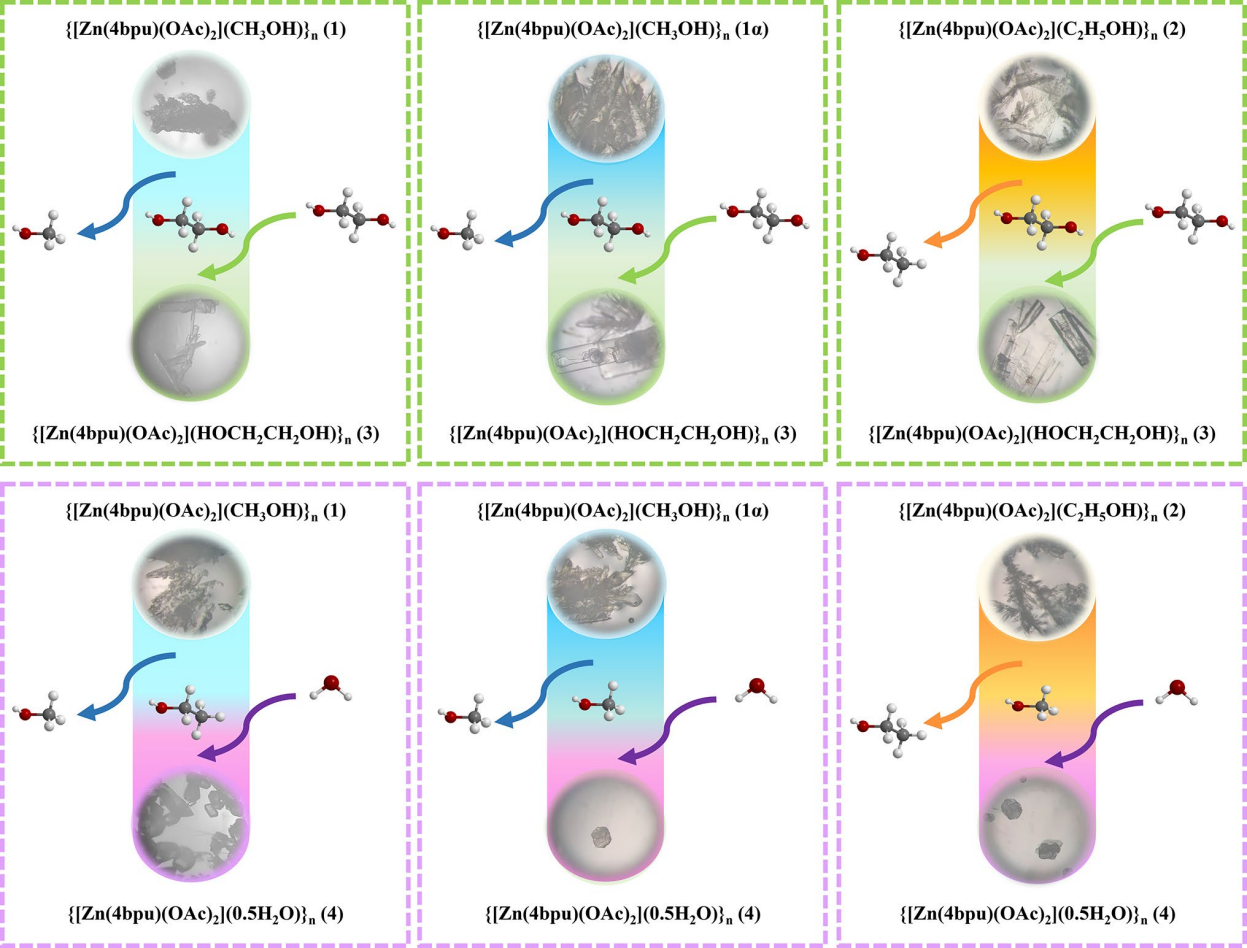
Transformation of **1**, **1α** and **2** through a DRST process.

Sometimes, a synthesized crystalline compound can be utilized as a precursor for the synthesis of another novel crystalline material. Although, most structural transformations are focused on single-crystal-to-single-crystal (SC-SC) structural conversion, in some cases this transformation is performed through the dissolution-recrystallization structural transformation (DRST) approach^30,32,35,90,91^. To investigate the structural transformation, several fresh single crystals of all the synthesized compounds were kept separately in different solvents such as MeOH, EtOH, ethylene glycol, and propylene glycol. The structural transformations observed are represented in Fig. 9. In the first step, single crystals of compounds **1**, **2** and **1α** were dissolved in ethylene glycol and after a few days, new single crystals were formed in the container. X-ray single-crystal unit cell dimension analysis of the obtained crystals showed that they are identical to compound **3**. The fresh single crystals of **2** and **1α** dissolved in MeOH and the structural transformation observed through DRST process are shown in Fig. 9. The result of single-crystal X-ray analysis of these new crystals revealed that they are identical to compound **4**. In the same manner the fresh single crystals of **1** immersed in EtOH and the DRST structural transformations observed to compound **4** (Fig. 9).

The result of synthetic experiments and structural transformations using the DRST method in our previous work, showed that the water content in the reaction vessel plays a crucial role in the formation of pseudopolymorphic CPs^35^. These results demonstrate that the transformation between pseudopolymorph CPs is achievable through the DRST process. It should be noted that in other cases transformation (SC-SC or DRST) was not observed and the outcomes of these tests are summarized in Table S10.

### Spodium bonds (SpBs) in compounds 1−4 and 1α

**Fig. 10:**
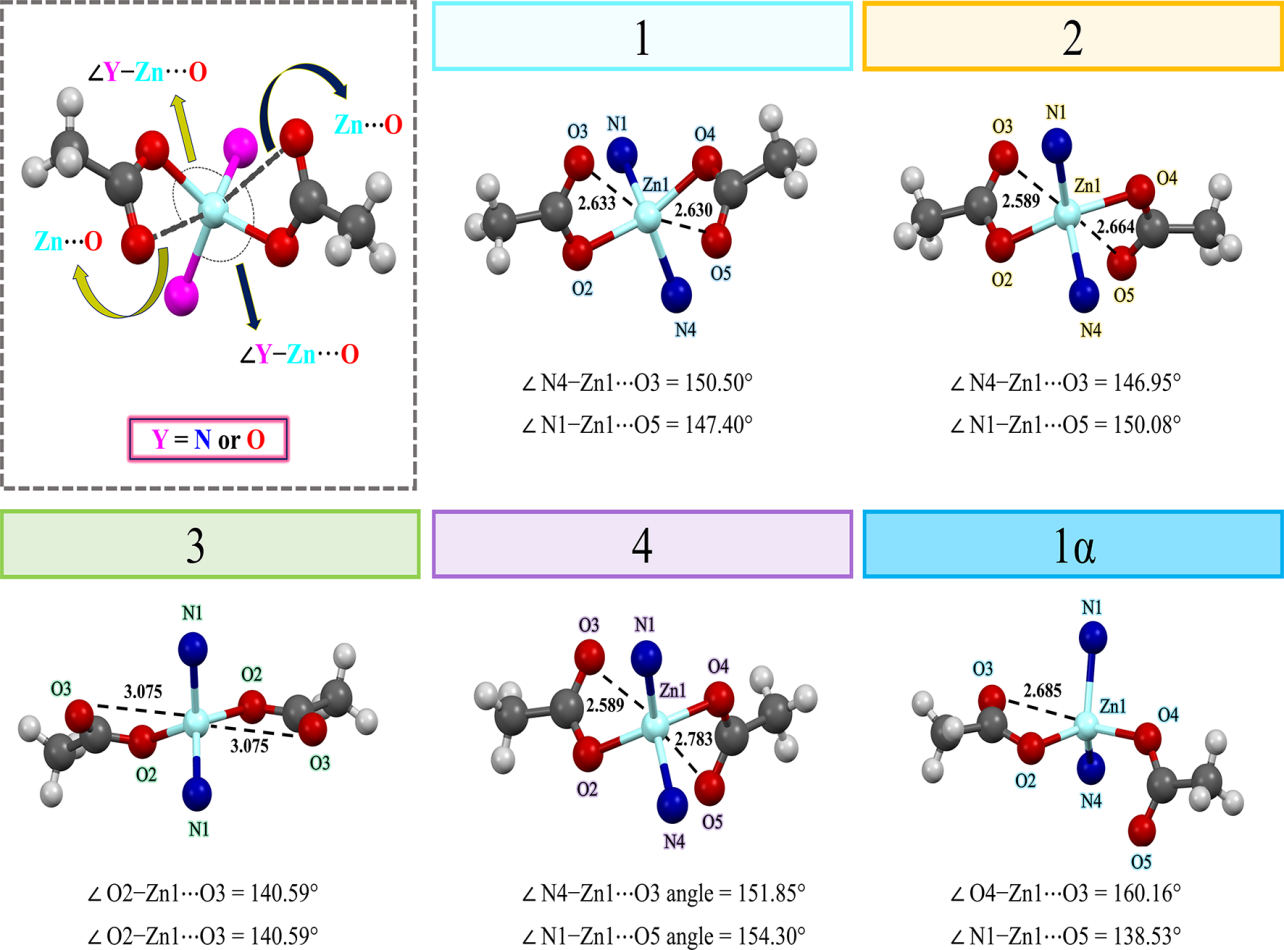
Geometric parameters of spodium bond formed with the uncoordinated oxygen atom of acetate anions. Coordination environment of the structures **1–4** and **1α** showing the spodium bonds (distances are in Å).

The concept of the spodium bonding is relevant to non-covalent interactions. The importance of intramolecular spodium bonds in Zn(II) complexes have been recently discussed^66,67,92^. As depicted in Fig. 10 and Table S11, one of the most striking findings in the crystal structure of **1**, **2** and **4** is the existence of Zn1⋯O3 and Zn1⋯O5 intramolecular spodium bonds, which are formed with the uncoordinated oxygen atom of acetate anions. Both interactions are longer than the sum of the covalent radii of zinc and oxygen atoms ($\:\sum{R}_{\text{cov}}\text{ = 1.88}\:$Å) and smaller than the sum of the Bondi’s van der Waals radii of these atoms ($\sum{R}_{\text{vdW}}=\text{2.91}\:$Å)^93^. The bond angles of N4−Zn1⋯O3 and N1−Zn1⋯O5 in **1**,** 2** and **4** are in the accepted range of spodium bond [140° ≤ θ (∠Y−Zn···A) ≤ 180° where Y, A = (N or O)]^94^.

Also, particularly notable finding in the crystal structure of **3** as illustrated in Fig. 10 and Table S11, is the formation of Zn1⋯O3 intramolecular spodium bonds, which are formed with the uncoordinated O3 oxygen atom of coordinated acetate anions. Both interactions are longer than the sum of covalent radii ($\:\sum{R}_{\text{cov}}\text{ = 1.88}$ Å) and according to Hu and Robertson (H&R) radius, are smaller than the sum of the H&R’s van der Waals radii ($\sum{R}_{\text{vdW}}=\text{3.56}\:$Å), respectively^95^. The bond angles of O2−Zn1⋯O3 in **3** are in the accepted range of spodium bond, [140° ≤ θ (∠Y−Zn···A) ≤ 180° where Y, A = (N or O)]^94^. Considering the spodium bonds, the zinc metal centers can be considered as a six-coordination geometry.

According to Fig. 10, only one of the acetate groups in compound **1α** participate in intramolecular spodium bonds with the Zn metal center. Intramolecular spodium bonds of Zn1⋯O3 formed with the uncoordinated oxygen atom of acetate anions in **1α** (Table S11). This interaction is longer than the sum of the covalent radii of zinc and oxygen atoms ($\:\sum{R}_{\text{cov}}\text{ = 1.88}\:$Å) and smaller than the sum of the Bondi’s van der Waals radii of these atoms ($\:\sum{R}_{\text{vdW}}=\text{2.91}\:$Å)^93^. The bond angle of O4−Zn1⋯O3 in **1α** is in the accepted range of spodium bond [140° ≤ θ (∠Y−Zn···A) ≤ 180° where Y, A = (N or O)]^94^. Due to the spodium bond, the metal center now exhibits a five coordinated geometry.

**Fig. 11:**
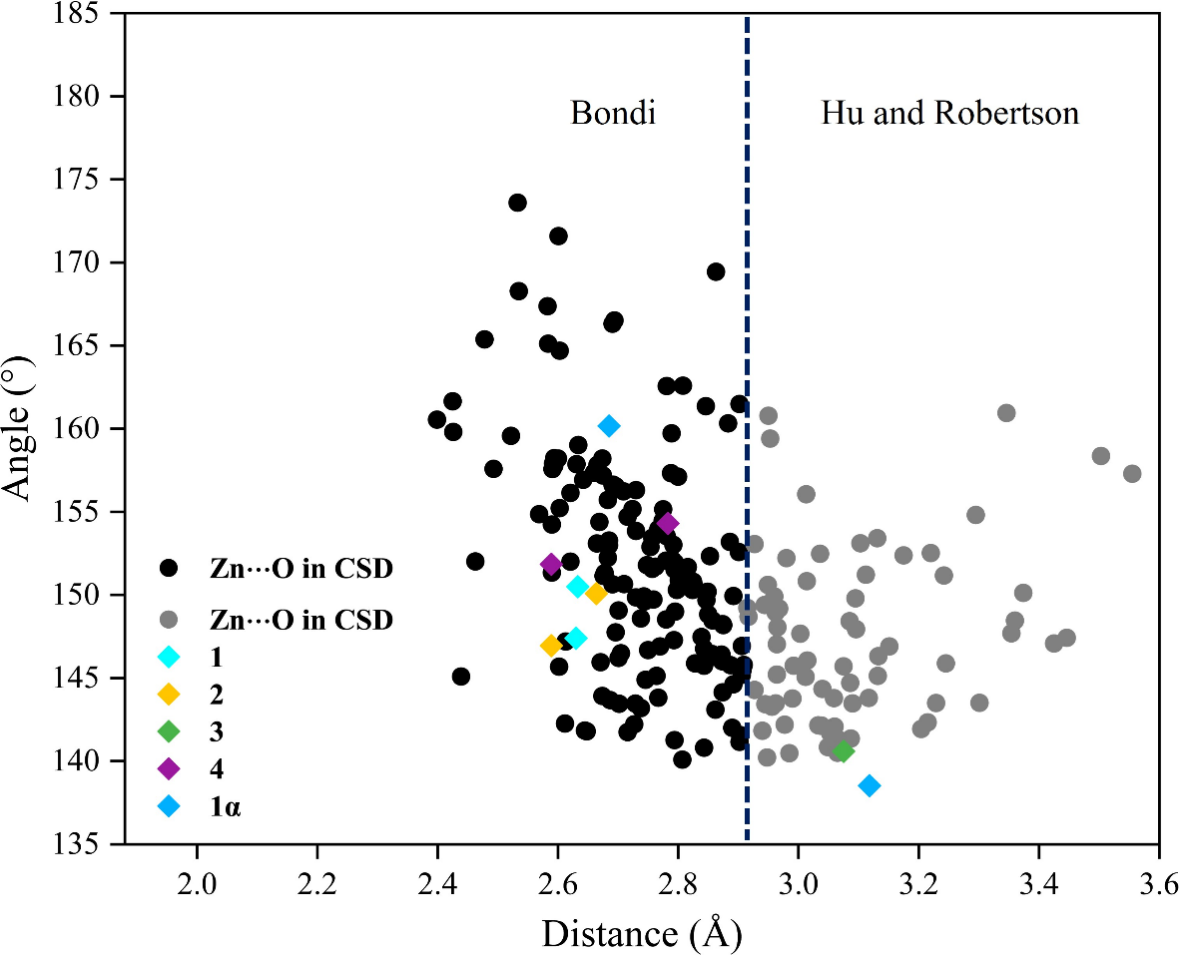
Scatter plot of Y−Zn···O (Y = N or O) angle versus the Zn···O distance that are in acceptable range of spodium bond extracted from the CSD data. The vertical dashed lines mark the data involved in sum of the van der Waals radius overlap according to Bondi’s and Hu and Robertson van der Waals radius for zinc and oxygen atoms.

Moreover, a statistical analysis of Cambridge Structural Database (CSD) with the help of ConQuest version 5.45^96^, evidences that the spodium bonding is somewhat directional, further supporting the relevance of this interaction in crystal engineering. This search particularly with contribution of acetate anion in coordination sphere has conducted. The processing of the obtained data allowed the recognition of several more examples of these novel intramolecular interactions. As evident from the histogram plot in Fig. 11, both the sum of van der Waals radius according to Bondi and H&R have been considered within the specified range.

### Analysis of Hirshfeld surfaces

**Fig. 12:**
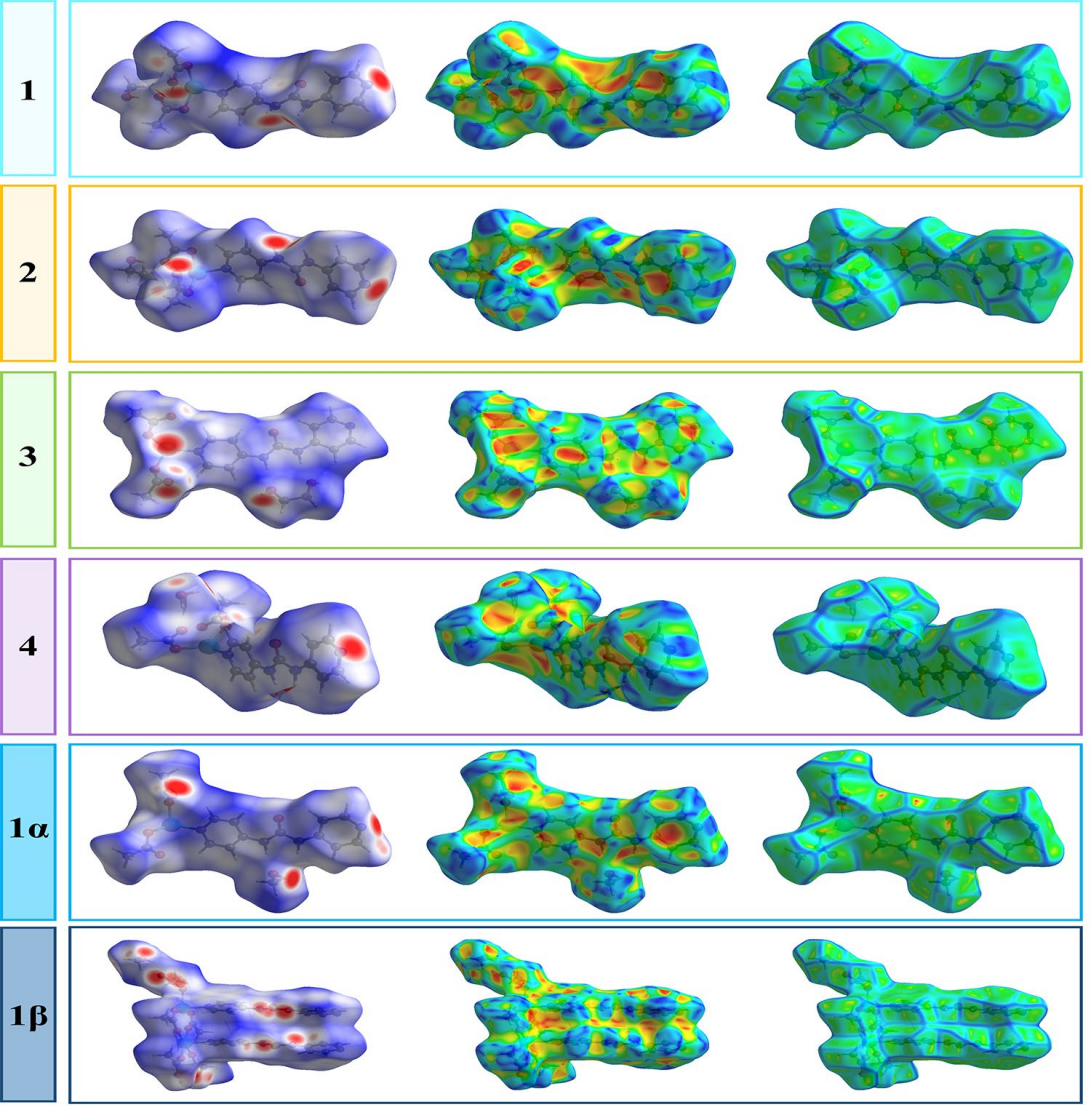
Hirshfeld surfaces mapped with *d*_norm_ (left side), shape index (middle) and curvedness (right side) for the presented compounds.

The supramolecular interactions around the coordination polymers are further analyzed by studying the Hirshfeld surfaces (HSs) and 2D fingerprint plots (FPs), which are generated by using CrystalExplorer^97^ based on the CIF files, including those to differentiate between pseudopolymorphs and supramolecular isomers. The HSs give additional insight into the long- and short-range interactions experienced by the molecules. Moreover, the FPs that derived from the Hirshfeld surfaces, provide detailed information about the nature, type, and relative contributions of the intermolecular interactions. The HSs of compounds are illustrated in Fig. 12, showing surfaces that have been mapped over the normalized contact distance (*d*_norm_) range of -0.5 to 1.5 Å, shape index (-1.0 to 1.0 Å) and curvedness (-4.0 to 0.4 Å).

The surfaces are transparent to permit visualization of the asymmetric unit of each coordination polymer. The geometric parameters presented in Table S8 and S9 are summarized effectively by the deep red spots visible on the *d*_norm_ surfaces, indicative of hydrogen bonds and other weak interactions like H···H contacts. As depicted in Figure S23, the two main contributors to the Hirshfeld surfaces are H···H, ranging from 37.5% (**4**) to 52.9% (**1β**), O···H/H···O, ranging from 19.6% (**3**) to 31.7% (**4**). The significant difference of the H⋯H interactions between the structures of each compound is reflected in the fingerprint plots which spread only up to *d*_i _= *d*_e_ = 1.186 Å in **1**, *d*_i_ = *d*_e_ = 1.174 Å in **2**, *d*_i_ = *d*_e_ = 1.072 Å in **3**, *d*_i_ = *d*_e_ = 0.888 Å in **4**, *d*_i_ = *d*_e_ = 1.136 Å in **1α** and *d*_i _= *d*_e_ = 0.848 Å in **1β**. In all cases, the O···H/H···O interactions are evidenced by the two distinct spikes in the (*d*_i_, *d*_e_) region of (1.120, 0.780), (1.070, 0.720), (1.120, 0.780), (1.100, 0.760), (1, 0.640) and (1.090, 0.740) for **1−4**, **1α** and **1β**, respectively. According to FPs, the other prominent contributor to the Hirshfeld surfaces is C⋯H/H⋯C close contact, that attributed to C−H⋯π interaction and comprises 13.7, 13.4, 5.8, 16.2, 15.3 and 7.6% of the total surface for each molecule in **1−4**, **1α** and **1β**, respectively. The total percentage contributions to the surface contacts are represented graphically in Figure S24.

As shown in Figure S25, the Zn−N bonds on both sides of the Hirshfeld surface mapped over *d*_norm_ for all compounds appear as light red spots perpendicular to the Zn−N bond direction. The strength of the Z−N bonds is also evident as the rectangular orange spots and the green flat regions on the Hirshfeld surface mapped with shape-index and over curvedness, respectively. Similar trends to those just described are evident for the Zn−O bonds. The orange patches of irregular shape surrounded by yellow regions on the shape-index mapped Hirshfeld surfaces about the zinc centers facing the oxygen atoms are indicative of differences in the Zn−O bond lengths.

The small red regions on the Hirshfeld surfaces mapped over curvature reflect the nature and strength of the Zn−O bonds. As depicted in Figure S26, the zinc coordination environments are also rationalized in the FPs considering only the HSs about the central atom for each of the compounds. It is evident in all fingerprint plots that the long red spikes indicate Zn−N bonds, whereas the red pencil tips are due to the Zn−O bonds. This data extracted from Hirshfeld surfaces confirm the presence of intramolecular spodium bond in **1**, **2**, **4** and **1α**. For compound **3**, since the accepted range of the spodium bond was determined by the sum of the H&R’s van der Waals radii, and considering the specific orientation of the uncoordinated oxygen atoms of the acetate anions for forming hydrogen bonds with adjacent acetate anions and ethylene glycol guest molecule, the intramolecular spodium bond cannot be analyzed by Hirshfeld surfaces.

### FT-IR studies

The FT-IR spectrum of the **4bpu** ligand and compounds **1−4**,** 1α** and **1β** was measured using the KBr pellet (Figures S3–S9). All compounds exhibited vibrations related to C=O urea, N–H urea, C=C aromatic, COO acetate, O–H and C–H groups in their spectrum. The stretching vibration peak of C=O and N–H groups of urea function in **4bpu** ligand are centered at 1739 and 3402 cm^−1^, respectively^72^. From SC-XRD analysis, it is clear that the **4bpu** ligand in compounds **1**, **2**, **4** and **1α** do not display any hydrogen bonding involving the C=O urea functionality. Thus, the C=O urea stretching band in the range of 1739–1746 cm^−1^ for compounds **1**, **2**, **4** and **1α** correspond well with that obtained for **4bpu** ligand, whereas slightly low-energy shift of the corresponding bands in the range of 1725–1730 cm^−1^ for the compounds **3** and **1β** could be due to its participation in hydrogen bonding. It is noteworthy that in all the structures studied in this work, the urea N–H is involved in N–H⋯O hydrogen bonding and the corresponding N–H bending bands^98^ appear in the range of 1590–1628 cm^−1^. The free acetate ion exhibits the symmetric and asymmetric stretching vibration at 1414 cm^−1^ and 1578 cm^−1^, respectively^99^. The covalently bonded acetate to metal by monodentate coordination mode, shifted asymmetric stretching vibration to higher frequencies. Two spectral bands appearing in the range of 1412–1431 cm^−1^ and 1590–1598 cm^−1^ confirm the presence of acetate anion with monodentate coordination mode in compounds **1−4** and **1α**. Two spectral bands appearing in 1420 cm^−1^ and 1564 cm^−1^ confirm the presence of acetate anion with chelate-bridging coordination mode in compound **1β**. The medium spectral bands in the range of 619 to 623 cm^−1^ corresponds to bending vibrational frequencies of the O–C–O acetate group^81,99^. Above 3000 cm^−1^, weak spectral bands in the range of 3292 to 3449 cm^−1^ corresponds to stretching vibrational frequencies of the N–H urea group and indicate the presence of guest water molecules in **4**. Also, vibrational frequencies of the C–H aromatic fall from 3010 to 3092 cm^−1^ that appears with a medium peak^25,100,101^.

### Powder X-ray diffraction (PXRD) and thermogravimetric analysis (TGA)

To identify the bulk purity of compounds, their powder X-ray diffraction (PXRD) patterns were investigated. As shown in Figures S27–S32, the experimental patterns match quite well with those of the corresponding simulated patterns, indicating the bulk purity of all compounds.

Thermal gravimetric analysis (TGA) of compounds was carried out to estimate the thermal decomposition of the frameworks. The thermal stability of compounds was measured under nitrogen atmosphere in the temperature range of 25–800 ℃ with a heating rate of 10 °C/min (Figure S33). The TGA curve of **1** shows the gradual weight loss of the one MeOH molecule (calculated 7.46 wt %; observed 7.75 wt %) from 53 °C to 172 °C. The weight loss of 76.49 wt % in the temperature range of 172–486 °C corresponds to the decomposition of two acetate anions and one **4bpu** ligand (calculated 77.32 wt %). For compound **2**, in the temperature range of 57 °C to 214 °C, the weight loss of 10.03 wt % can be attributed to the loss of one EtOH solvent molecule (calculated 10.38 wt %). The second step starts from 214°C; the gradual weight loss of 70.10 wt % till 800 °C corresponds to the decomposition of two acetate anions and one **4bpu** ligand (calculated 74.88 wt %). The TGA curve of compound **3** shows a multi-step mass loss of 13.76 wt % (calculated 13.49%) in the temperature range of 165–181 °C corresponds to the loss of one ethylene glycol molecule and the weight loss of 69.74 wt % in the temperature range of 181–800 °C corresponds to the decomposition of two acetate anions and one **4bpu** ligand (calculated 72.27 wt %). The TGA curve of **4** shows the gradual weight loss of the one H_2_O molecule (calculated 2.21 wt %; observed 2.34 wt %) from 73°C to 155 °C. The weight loss of 79.32 wt % at 155–800 °C which is attributed to decomposition of two acetate anions and one **4bpu** ligand (calculated 81.7 wt %). The TGA thermogram of **1α** shows the gradual weight loss of the one MeOH molecule (calculated 7.46 wt %; observed 5.63 wt %) from 73°C to 169 °C. The weight loss of 75.83 wt % at 169–800 °C which is attributed to the decomposition of two acetate anions and one **4bpu** ligand (calculated 77.33 wt %). In TGA curve of **1β** there are two obvious weight loss steps. First step shows the gradual weight loss of the two MeOH molecule (calculated 5.09 wt %; observed 5 wt %) from 44 °C to 175 °C and the second step demonstrated the weight loss of 79.91 wt % at 175–800 °C which is attributed to the decomposition of six acetate anions and three **4bpu** ligand (calculated 79.29 wt %).

## Conclusion

Using a ditopic ligand (**4bpu**) containing one urea functional group, solvent/additive dependent supramolecular isomers, including six Zn(II) coordination polymers, were prepared and structurally characterized. The structural differences in the four solvent-dependent supramolecular isomers (compounds **1**–**4)**, are related to urea-solvent interactions in the pseudopolymorphs, which prevented the intermolecular self-assembly process among the urea moieties (urea⋯urea hydrogen bonding of the α-tape motif). This underscores the critical role of solvent in influencing the final architecture of coordination polymers. Two additive-induced supramolecular isomers, compounds **1α** and **1β**, showing 1D chain in different manners (zig-zag 1D chain and triple-stranded ladder 1D chain), have been synthesized by controlling the use of additive (3-nitrophenol and 1,3-dinitrobenzene). One of the highlights of this work is the first-time synthesis of the rare triple-stranded ladder structure facilitated by the use of additive. The results of this research showed that the use of organic additives can be used as an effective approach to control supramolecular isomerism, achieve diversity in the structures of coordination polymers and help chemists to construct new supramolecular isomers. We have proved the presence of intramolecular spodium bonds in compounds **1–4** and **1α** between the four-coordinated Zn(II) ion and the oxygen atom of the acetate anion, which was within the accepted range of this interaction. These findings enhance the fundamental understanding of these non-covalent interactions. The various supramolecular interactions observed in the synthesized compounds, such as hydrogen bonds, lone pair–π, C−H⋯π and spodium bonds, play a critical role in stabilizing the structures. These supramolecular interactions maintain the integrity and robustness of the coordination polymers, ensuring their structural stability and contributing to the formation of diverse supramolecular isomers. We also succeeded for the first time in interconversion between pseudopolymorphs in coordination polymers via the DRST method simply by immersing the parent species in different solvents.

## Electronic supplementary material

Below is the link to the electronic supplementary material.

Supplementary Material 1

## Data Availability

“All other relevant data generated and analysed during this study, which include experimental, spectroscopic, crystallographic and computational data, are included in this article and its Supplementary Information.” CCDC 2,322,873 (1), 2,322,874 (2), 2,322,875 (3), 2,322,876 (4), 2,322,877 (1α) and 2,322,878 (1β) contains the supplementary crystallographic data for this paper. These data can be obtained free of charge via www.ccdc.cam.ac.uk/data_request/cif, or by emailing data_request@ccdc.cam.ac.uk, or by contacting The Cambridge Crystallographic Datacenter, 12 Union Road, Cambridge CB2 1EZ, UK. fax: +44 1,223,336,033.
