# Supplementary material for: Solvent and additive-controlled supramolecular isomerism in zinc coordination polymers
Source: Sci Rep. 2024 Nov 11;14:27586. doi: 10.1038/s41598-024-77298-6 (PMC11554826; doi:10.1038/s41598-024-77298-6)
Supplement: Supplementary file 1 — Supplementary Material 1 [file 41598_2024_77298_MOESM1_ESM.pdf]

Electronic Supplementary Information

Solvent and additive-controlled supramolecular isomerism in zinc coordination polymers

Ghazale Khorshidi & Behrouz Notash*

Department of Inorganic Chemistry, Shahid Beheshti University, 1983969411, Tehran, Iran. Fax: +98 2122431663; Tel: +98 2129904363.

Email: b_notash@sbu.ac.ir

Synthesis of 1,3-di(pyridin-4-yl)urea (**4bpu**) ligand……………………………..………………3
Figure S1. ^{1}H NMR spectrum of **4bpu** in DMSO-d_6, 300 MHz ……..………..…………….…..…4
Figure S2. ^{13}C NMR spectrum of **4bpu** in DMSO-d_6, 75 MHz………...……...…………….…..……5
Figure S3. FT-IR spectrum of **4bpu** in KBr………………….……………………...……….6
Figure S4. FT-IR spectrum of **1** in KBr……………………………….………………….……7
Figure S5. FT-IR spectrum of **2** in KBr………………...……….…………………………….…8
Figure S6. FT-IR spectrum of **3** in KBr……………………...….……………………….……9
Figure S7. FT-IR spectrum of **4** in KBr……………………..……………………………...…10
Figure S8. FT-IR spectrum of **1α** in KBr……………………….....…….…………………...…11
Figure S9. FT-IR spectrum of **1β** in KBr………………………..……………………..…...…12
Figure S10. Zn···Zn distances [Å] and Zn···Zn···Zn angles [°] in 1D zig-zag chain of **1** and **1α**…………………………………………………………………………………..….13
Figure S11. Zn···Zn distances [Å] and Zn···Zn···Zn angles [°] in 1D zig-zag chain of **2**–**4**……...……14
Figure S12. Zn···Zn distances [Å] and Zn···Zn···Zn angles [°] in 1D triple-stranded ladder of **1β**………………………………………………………………………………….……..15
Figure S13. Angles between pyridyl-urea and pyridyl-pyridyl planes of ligand in compound **1**……………………………………..………………………………………………16
Figure S14. Aangles between pyridyl-urea and pyridyl-pyridyl planes of ligand in compound **2**……………………………………..………………………………………………17
Figure S15. Angles between pyridyl-urea and pyridyl-pyridyl planes of ligand in compound **3**……………………………………..………………………………………………18
Figure S16. Angles between pyridyl-urea and pyridyl-pyridyl planes of ligand in compound **4**……………………………………..………………………………………………19

Figure S17. Angles between pyridyl-urea and pyridyl-pyridyl planes of ligand in compound **1α**……………………………………………………………………………………………20
Figure S18. Angles between pyridyl-urea and pyridyl-pyridyl planes of ligands in compound **1β**……………………………………………………………………………………………21
Figure S19. Comparison between ATR-FTIR spectra of compound **1** and the compounds obtained in the presence of different additives: as-synthesized **1**, **1α** and **1β**…………………………………22
Figure S20. Comparison between ATR-FTIR spectra of compound **2** and the compounds obtained in the presence of different additives: as-synthesized **2**…………………………………………..23
Figure S21. Comparison between ATR-FTIR spectra of compound **3** and the compounds obtained in the presence of different additives: as-synthesized **3**…………………………………………..24
Figure S22. Comparison between ATR-FTIR spectra of compound **3** and the compounds obtained in the presence of different additives: as-synthesized **3**…………………………………………..25
Figure S23. Fingerprint plots of compounds **1−4**, **1α** and **1β**………………………………..……….26
Figure S24. The relative contribution of different intermolecular interactions to the Hirshfeld surface area in the presented compounds…………………………………………………………..27
Figure S25. Views of Hirshfeld surfaces for the zinc center in the presented compounds mapped over d_{norm}, shape index and curvedness…………………………………………………………28
Figure S26. Comparison of the fingerprint plots calculated for the zinc center, in the presented compounds…………………………………………………………………………………………29
Figure S27. PXRD patterns of **1**. Black: Simulated from the X-ray single crystal data; Cyan: observed for the as-synthesized solids. ……………..…………………………………………...…30
Figure S28. PXRD patterns of **2**. Black: Simulated from the X-ray single crystal data; Orange: observed for the as-synthesized solids. ……………………..……………………………………...…31
Figure S29. PXRD patterns of **3**. Black: Simulated from the X-ray single crystal data; Green: observed for the as-synthesized solids. ……………………..……………………………………...…32
Figure S30. PXRD patterns of **4**. Black: Simulated from the X-ray single crystal data; Purple: observed for the as-synthesized solids. …………………………………………………..........…33
Figure S31. PXRD patterns of **1α**. Black: Simulated from the X-ray single crystal data; Light blue: observed for the as-synthesized solids. ……………………..……………………………………...…34
Figure S32. PXRD patterns of **1β**. Black: Simulated from the X-ray single crystal data; Dark blue: observed for the as-synthesized solids. ……………………..……………………………………...…35
Figure S33. The TGA diagrams of compounds **1**-**4**, **1α** and **1β**……….……..………………..….36
Scheme S1. Synthetic method, microscopic image and structural motifs for **1**, **1α** and **1β**…..…37
Table S1. Selected bond lengths [Å] and angles [°] for **1**…………………………………………..38
Table S2. Selected bond lengths [Å] and angles [°] for **2**…………………………………………..38
Table S3. Selected bond lengths [Å] and angles [°] for **3**…………………………………..……….38
Table S4. Selected bond lengths [Å] and angles [°] for **4**…………………………………………..38
Table S5. Selected bond lengths [Å] and angles [°] for **1α**………………………………………..….39
Table S6. Selected bond lengths [Å] and angles [°] for **1β** ………...………...………..………39
Table S7. Bond valence sums for zinc ions in compounds **1−4**, **1α** and **1β**……………………..40
Table S8. Hydrogen bonds geometry (D−H···A) for **1**, **1α** and **1β**……………………………41
Table S9. Hydrogen bonds geometry (D−H···A) for **2−4**…………………………………...……42

Table S10. Structural tranformation tests for **1−4**, **1α** and **1β**. ……………..…………..………..43
Table S11. Geometrical parameters of spodium bonds in **1−4** and **1α**………..……...…………...43
X-ray crystallography………………………………………………...……………………..44
References……………………………………………………….……………………………44

Synthesis of 1,3-di(pyridin-4-yl)urea (4bpu) ligand:

The ligand **4bpu** was synthesized according to the reported procedure.[1] Isonicotinic acid hydrazide (3 mmol, 410 mg) was dissolved in 10 ml 25% aq. HCl at 0 °C and $NaNO_2$ (5 mmol, 350 mg) dissolved in 5 mL ice cold water was added to it with stirring. Stirring was continued for 1 h, maintaining the temperature below 5 °C. The solution was neutralized by adding solid Na_2CO_3 and extracted with 65 ml toluene. The organic extracts were dried over Na_2SO_4 and filtered. 4-aminopyridine (3 mmol, 282 mg) was added to the filtrate and refluxed for 12 h. The precipitate was filtered and dried to afford **4bpu** in 70% yield. mp: 206–208 °C. ^{1}H NMR (DMSO-d_6, 300 MHz): δ = 7.47(d, 4H), 8.42(d, 4H), 9.36(s, 2H). ^{13}C NMR (DMSO-d_6, 75 MHz) δ = 122.97, 146.45, 150.70, 152.26. IR data (KBr pellet, cm^{-1}): 3402(m), 2219(w), 1940(w), 1831(w), 1739(s), 1640(s), 1604(s), 1586(s), 1537(s), 1517(s), 1496(s), 1423(s), 1335(s), 1325(m), 1286(s), 1245(m), 1191(s), 1004(s), 898(m), 828(s), 791(m), 737(m), 657(m), 646(m), 527(s) (Figure S4).

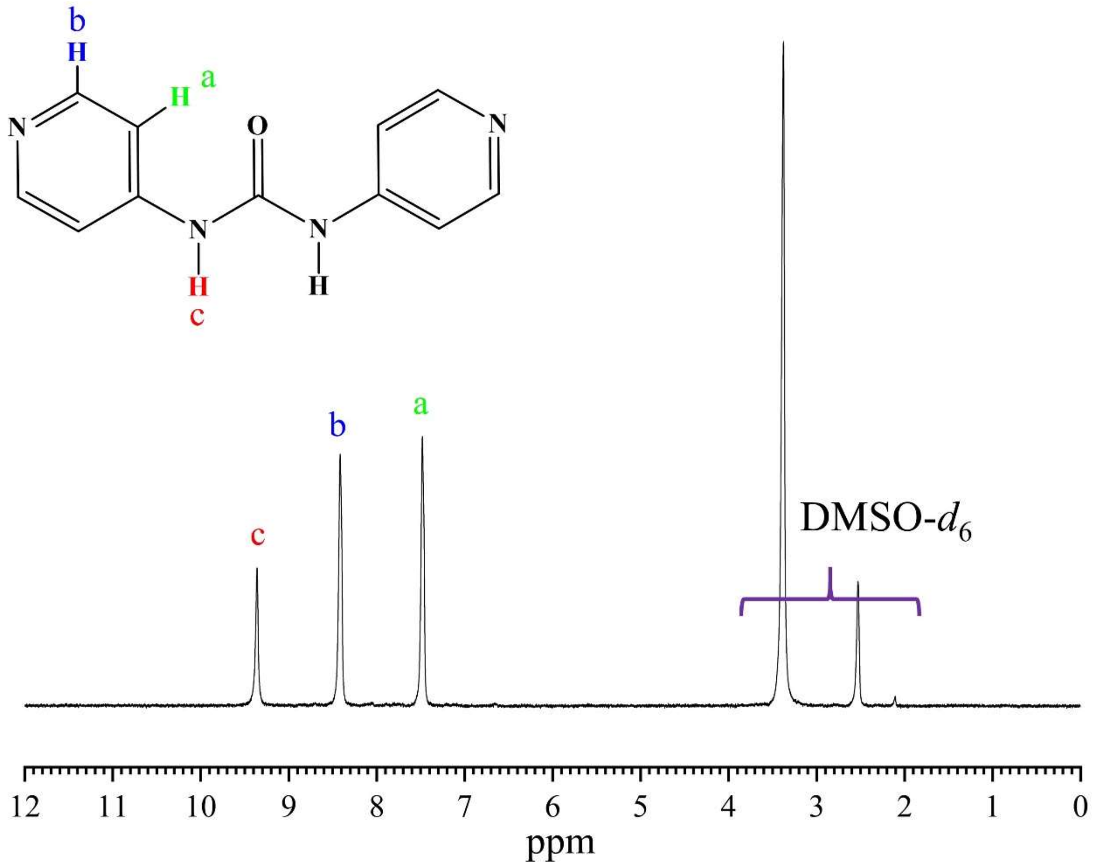

Figure S1. ^{1}H NMR spectrum of **4bpu** in DMSO-d_6, 300 MHz.

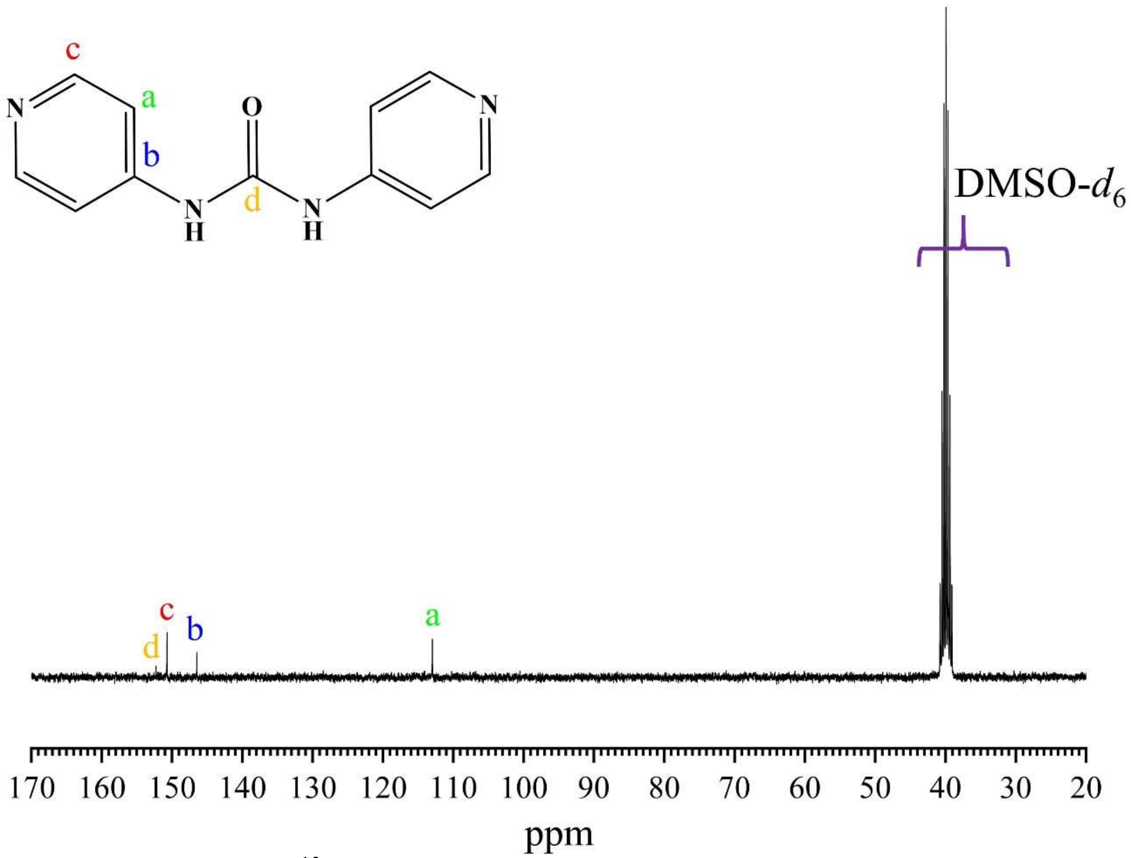

Figure S2. ^{13}C NMR spectrum of **4bpu** in DMSO-d_6, 75 MHz.

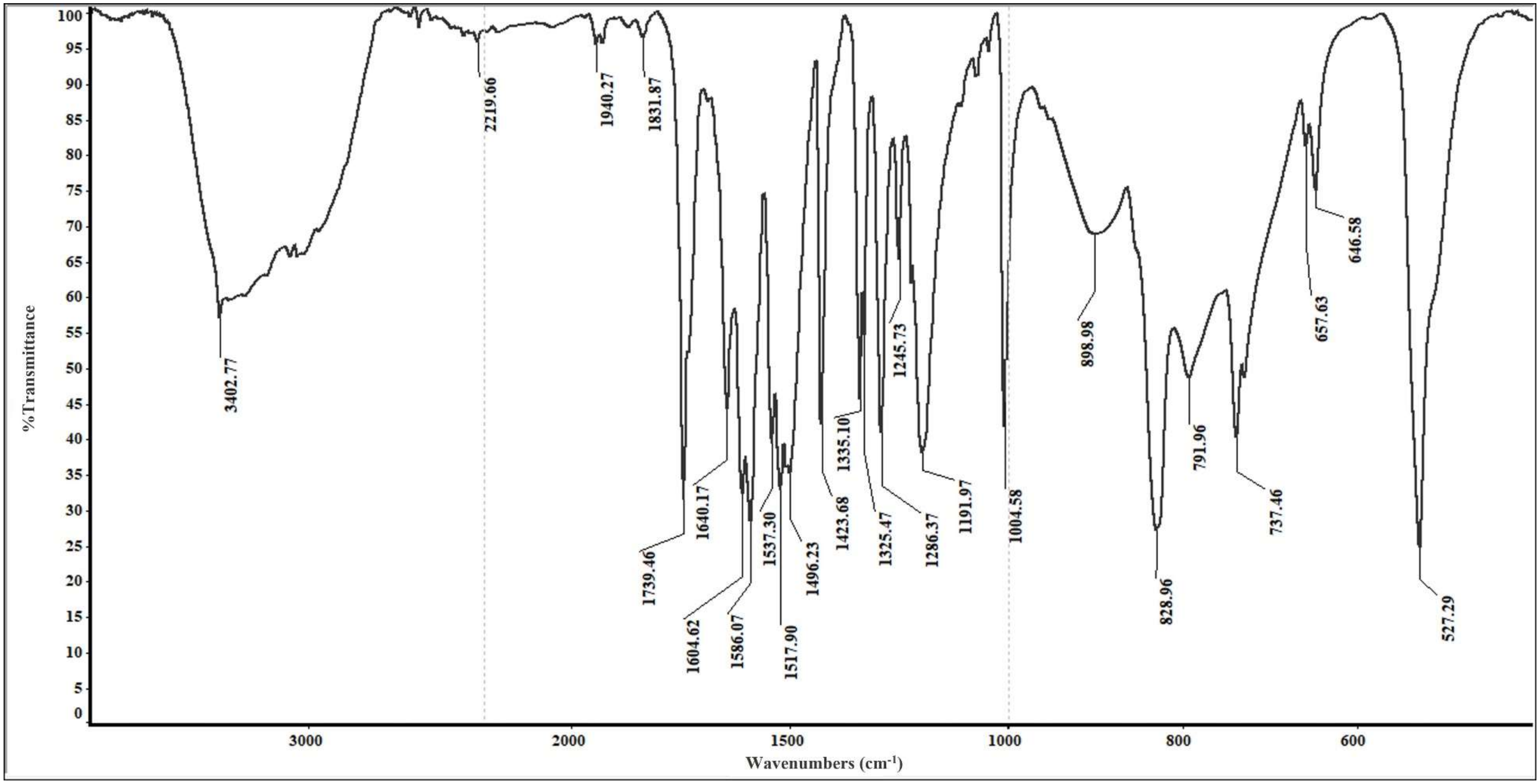

Figure S3. FT-IR spectrum of **4bpu** in KBr.

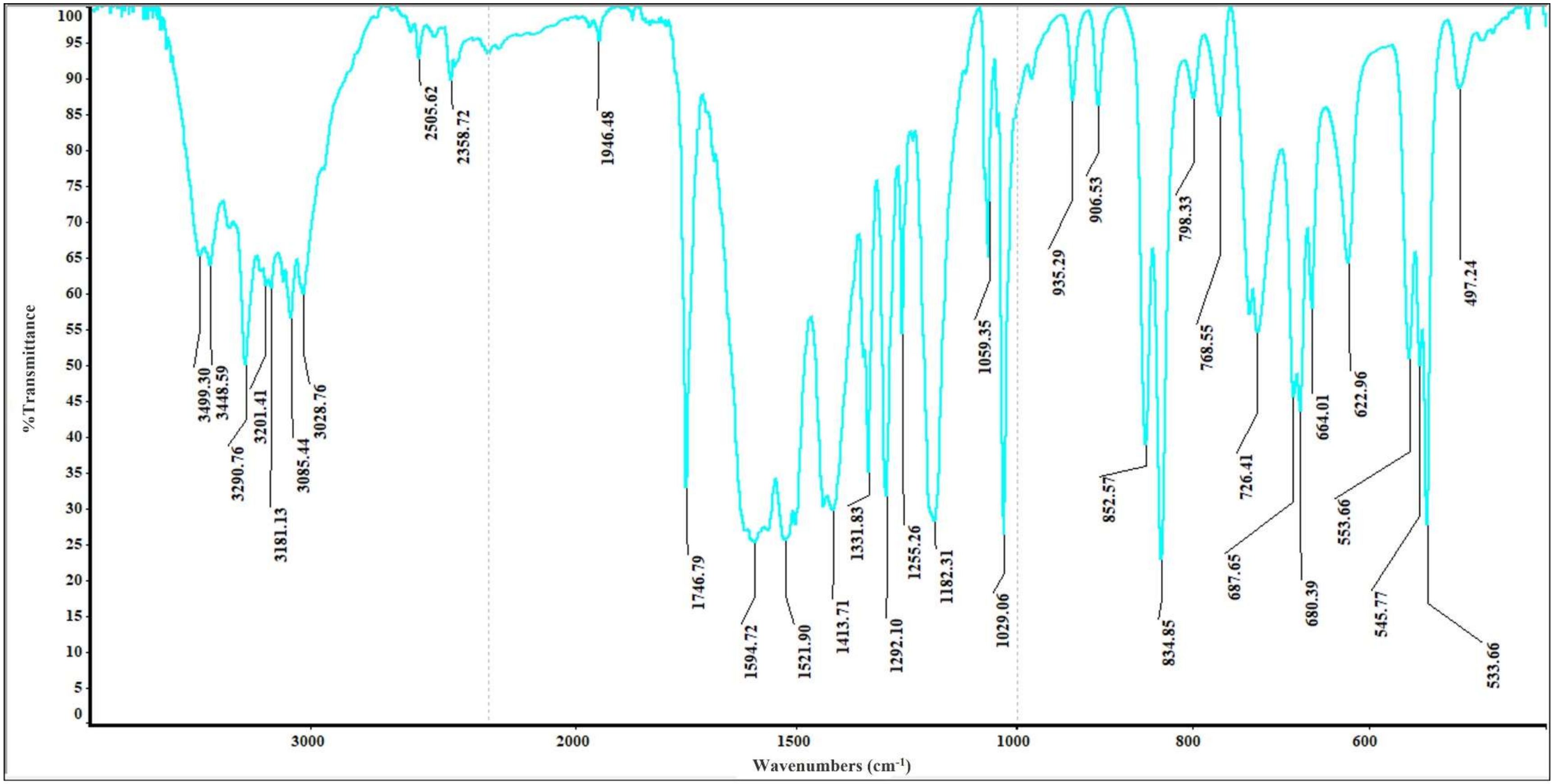

Figure S4. FT-IR spectrum of **1** in KBr.

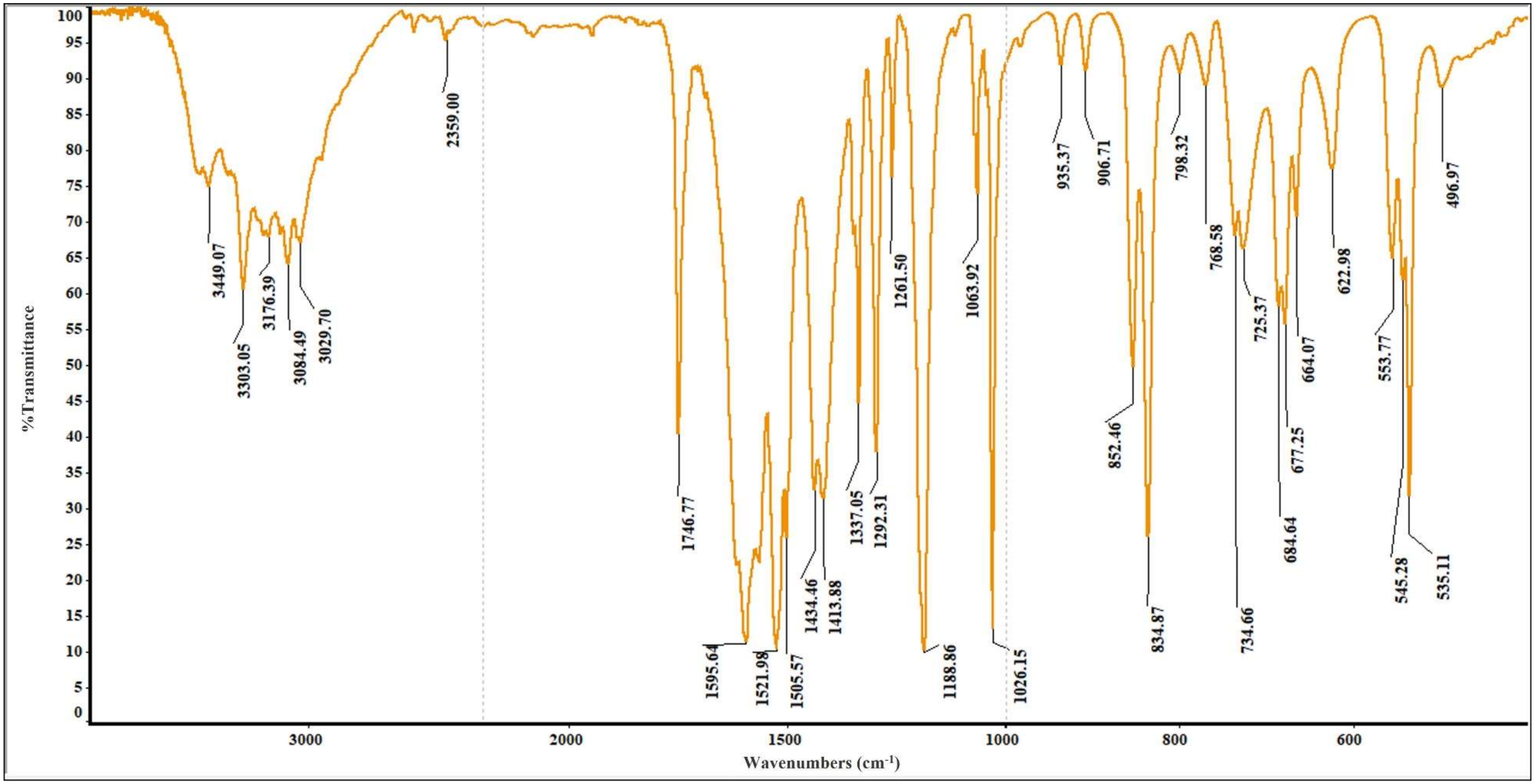

Figure S5. FT-IR spectrum of **2** in KBr.

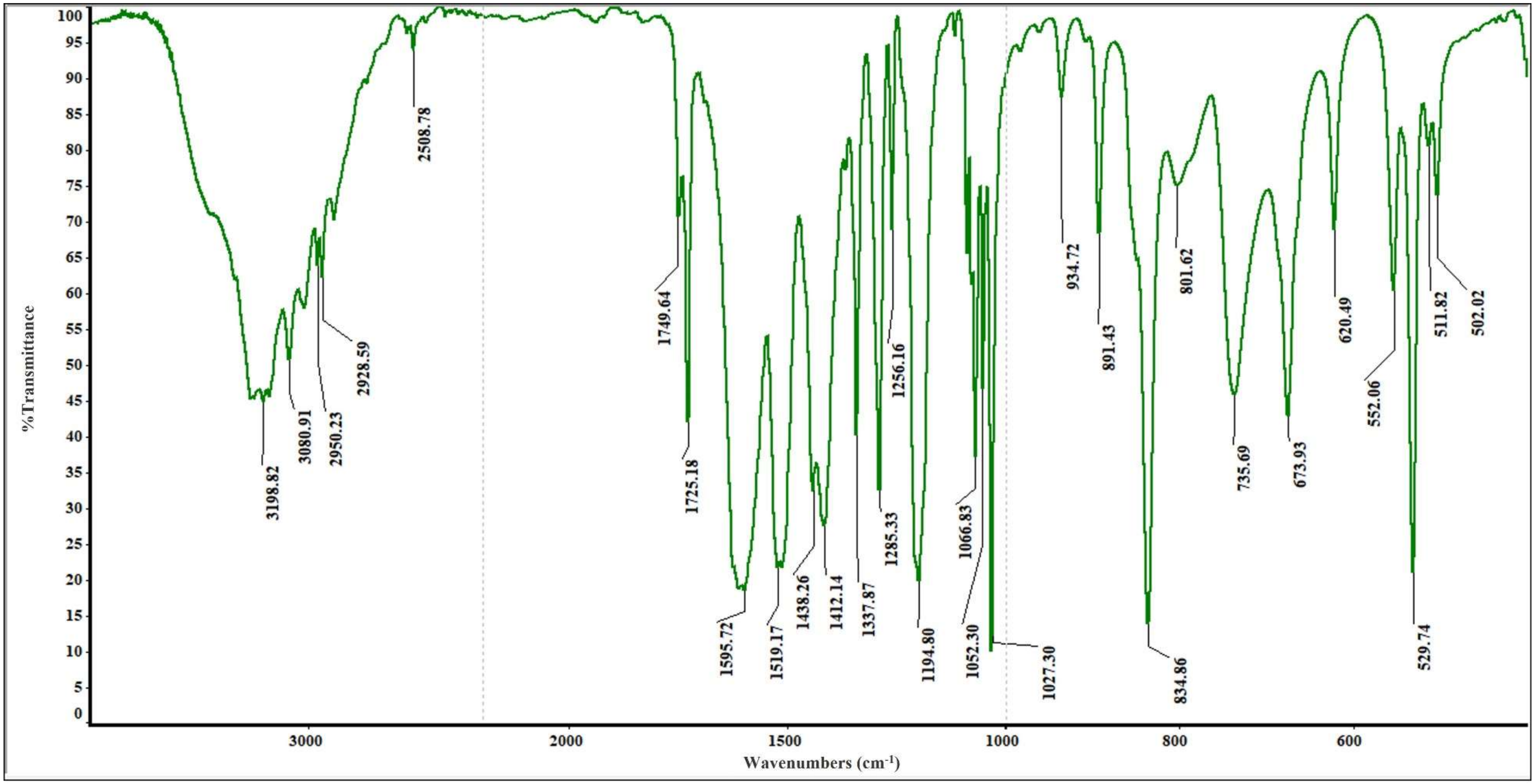

Figure S6. FT-IR spectrum of **3** in KBr.

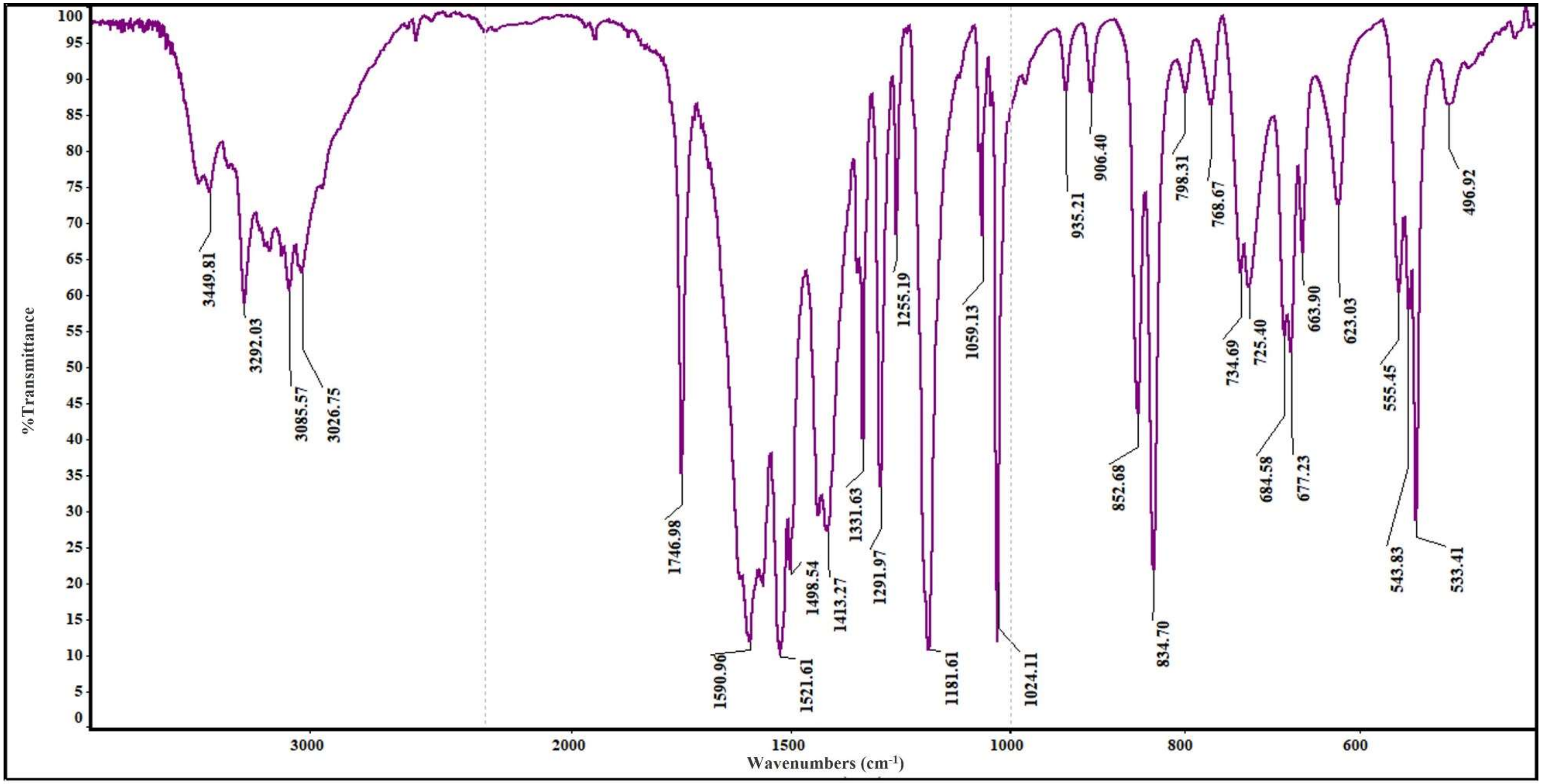

Figure S7. FT-IR spectrum of **4** in KBr.

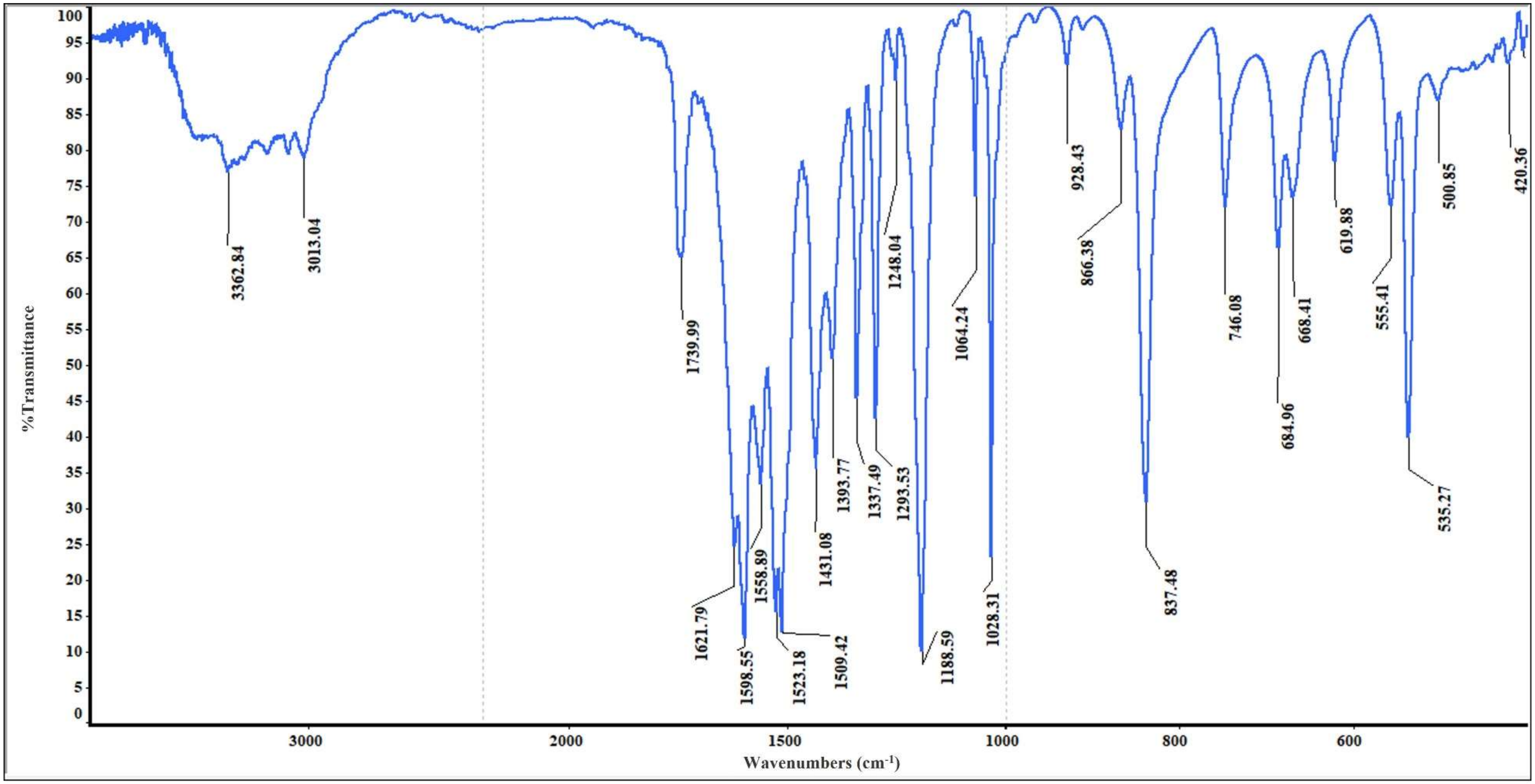

Figure S8. FT-IR spectrum of **1α** in KBr.

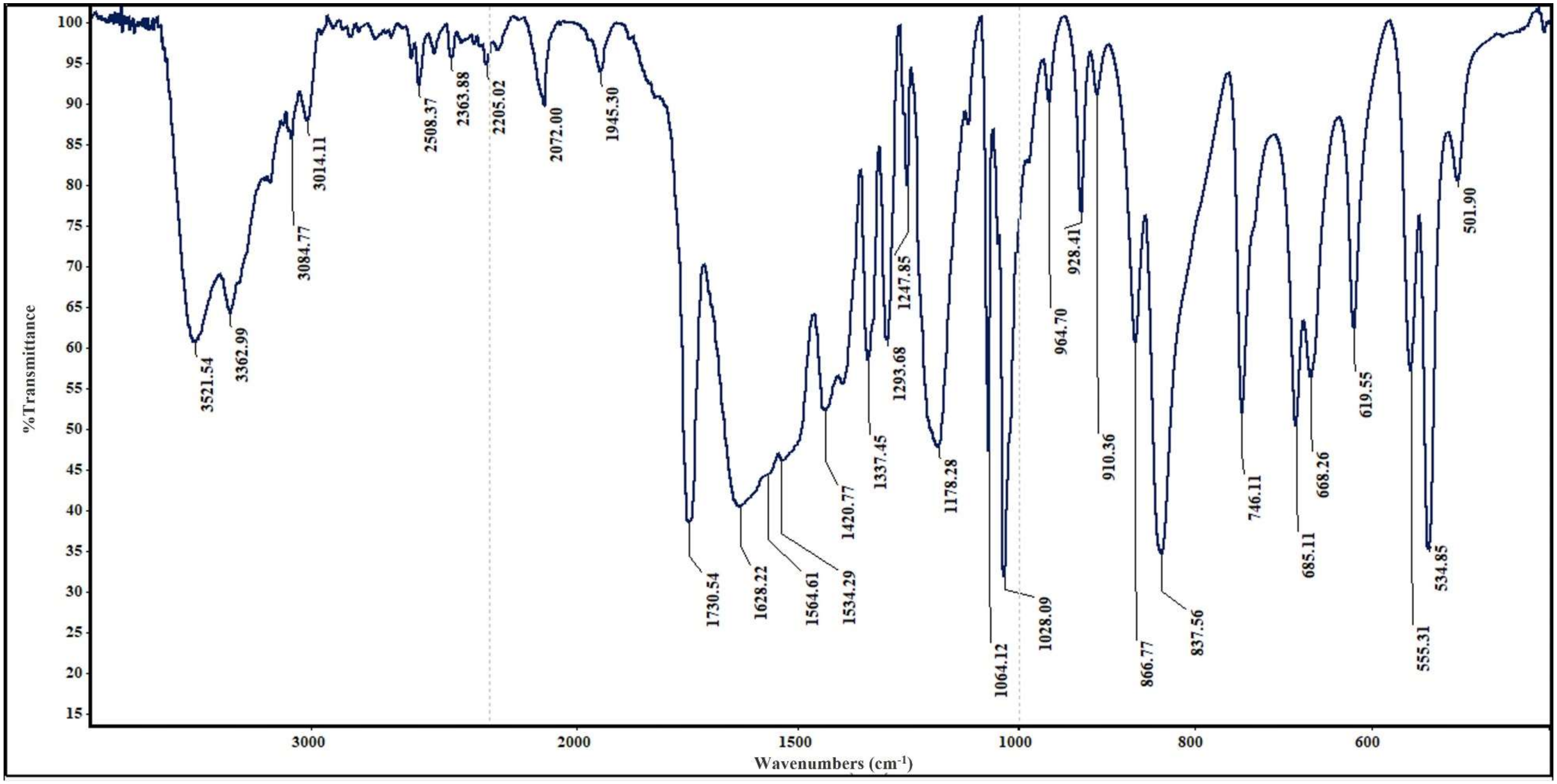

Figure S9. FT-IR spectrum of **1β** in KBr.

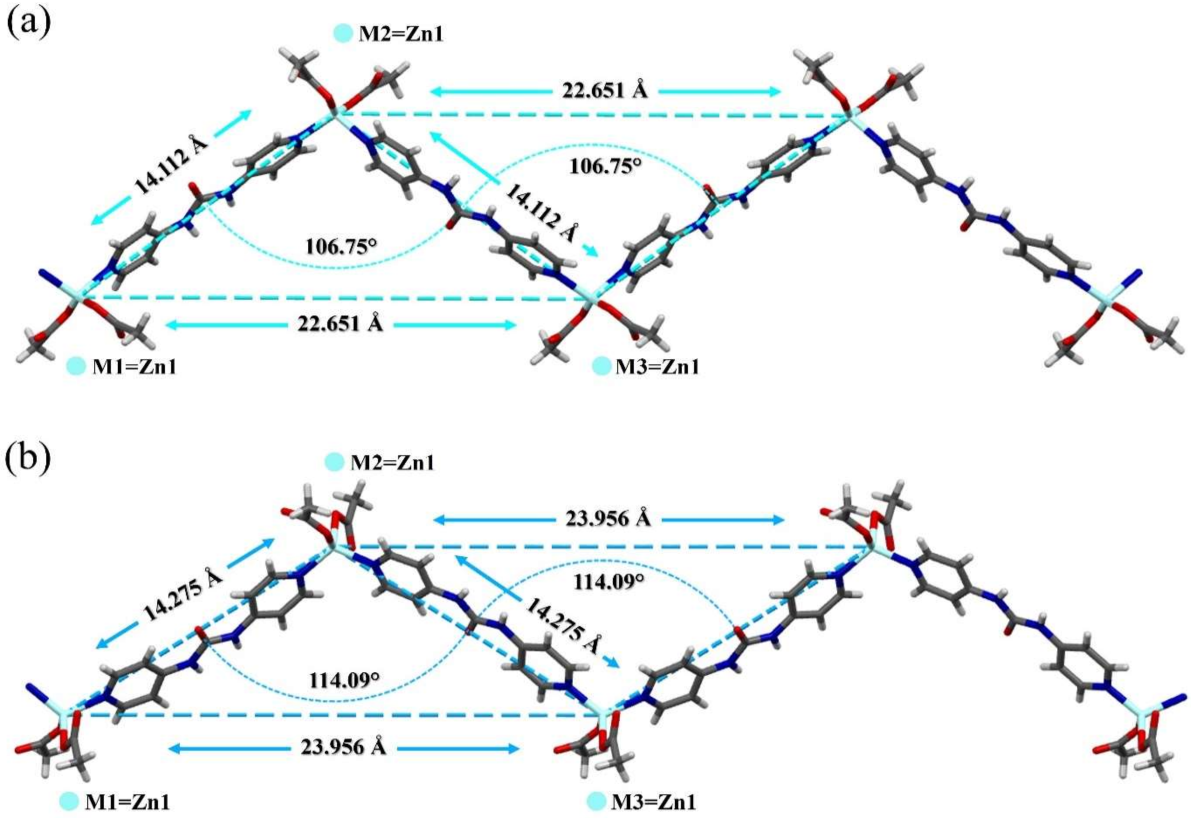

Figure S10. Zn···Zn distances [Å] and Zn···Zn···Zn angles [°] in 1D zig-zag chain of (a) **1** and (b) **1α**.

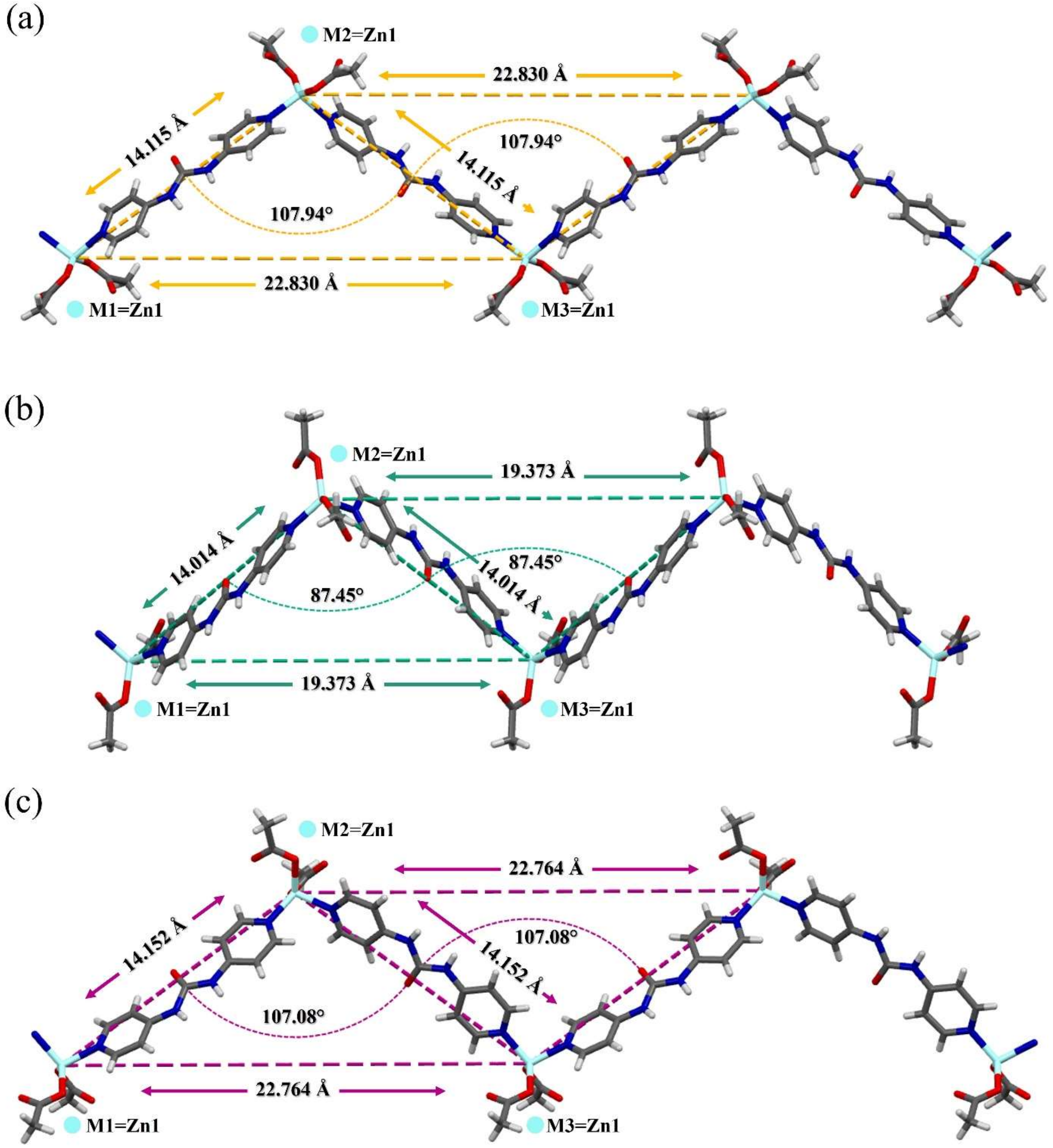

Figure S11. Zn···Zn distances [Å] and Zn···Zn···Zn angles [°] in 1D zig-zag chain of (a) **2**, (b) **3** and (c) **4**.

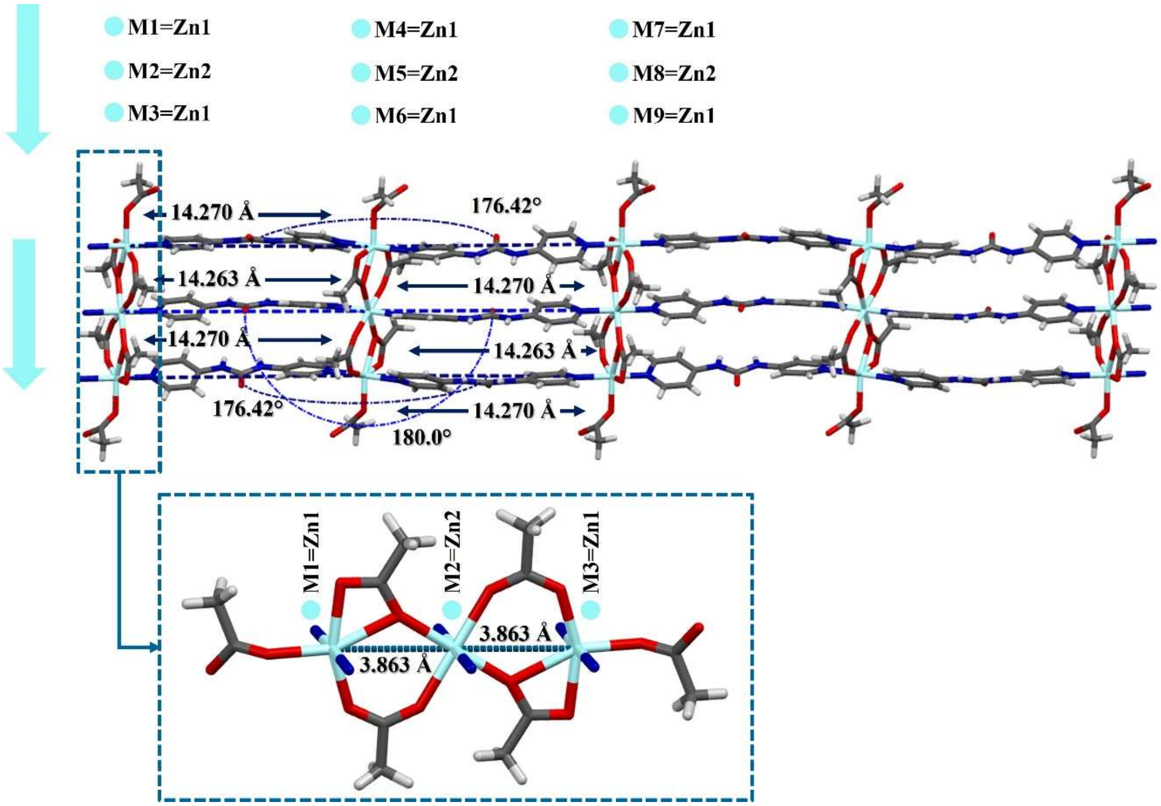

Figure S12. Zn···Zn distances [Å] and Zn···Zn···Zn angles [°] in 1D triple-stranded ladder of **1β**.

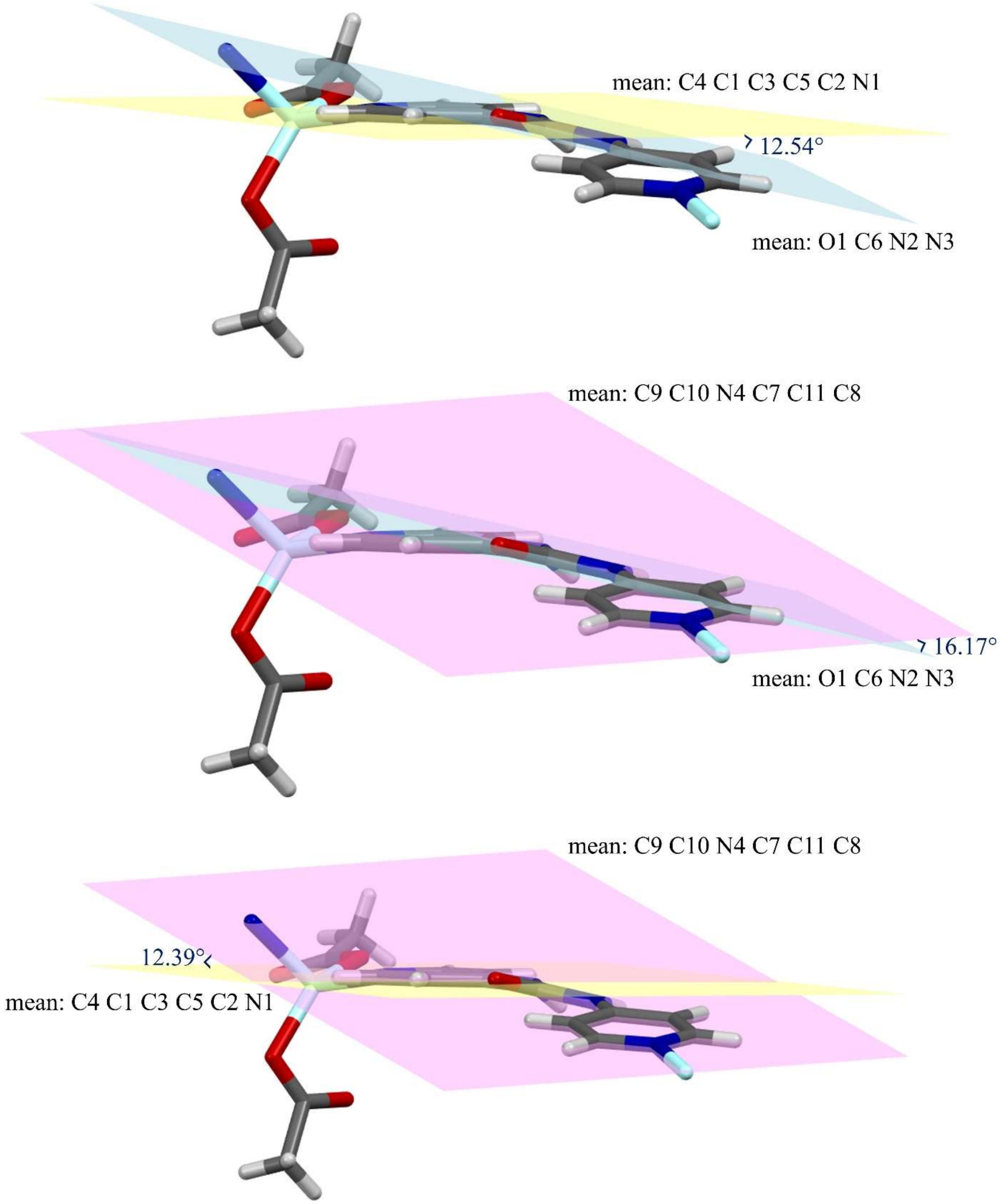

Figure S13. Angles between pyridyl-urea and pyridyl-pyridyl planes of ligand in compound **1**.

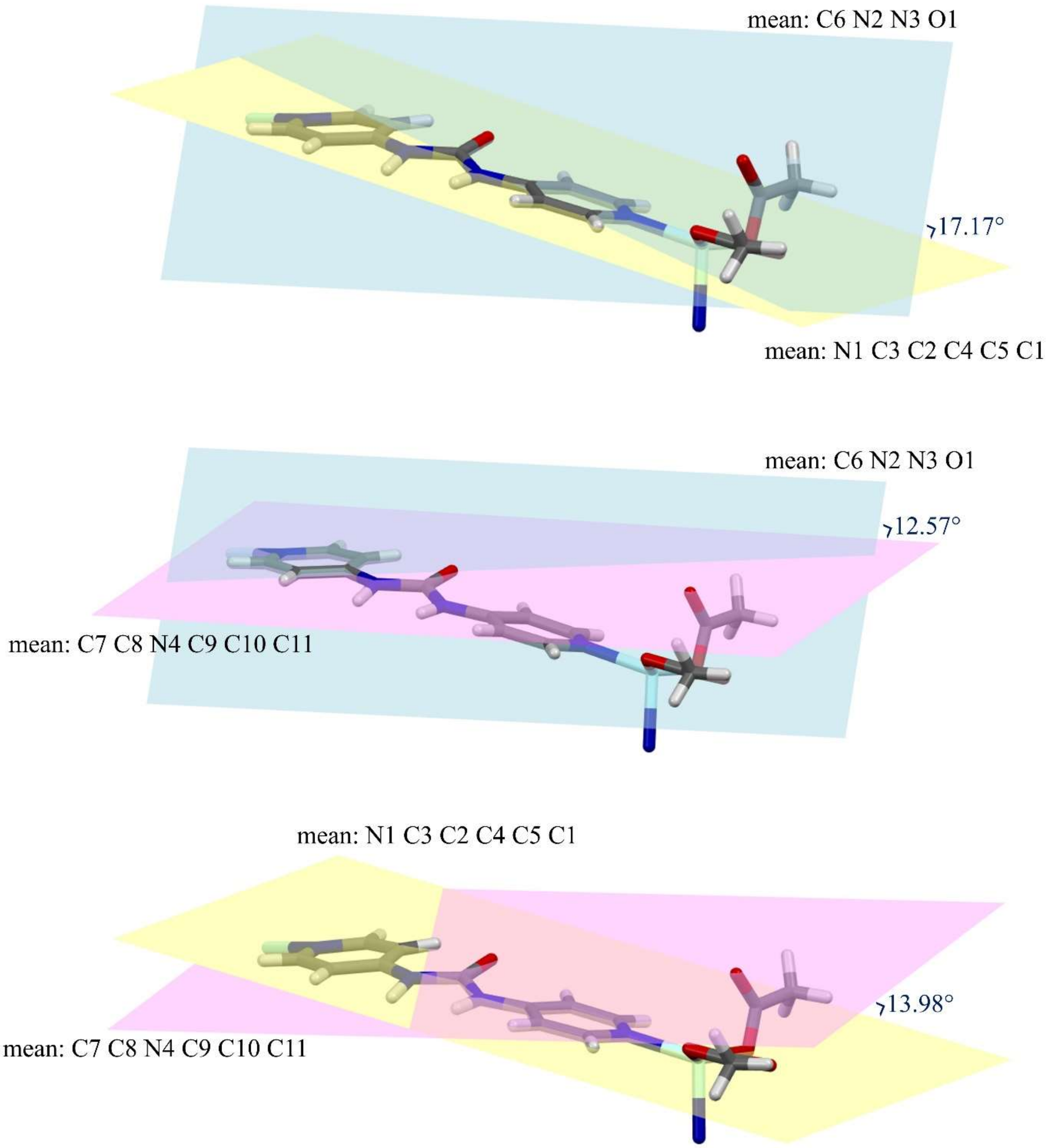

Figure S14. Angles between pyridyl-urea and pyridyl-pyridyl planes of ligand in compound **2**.

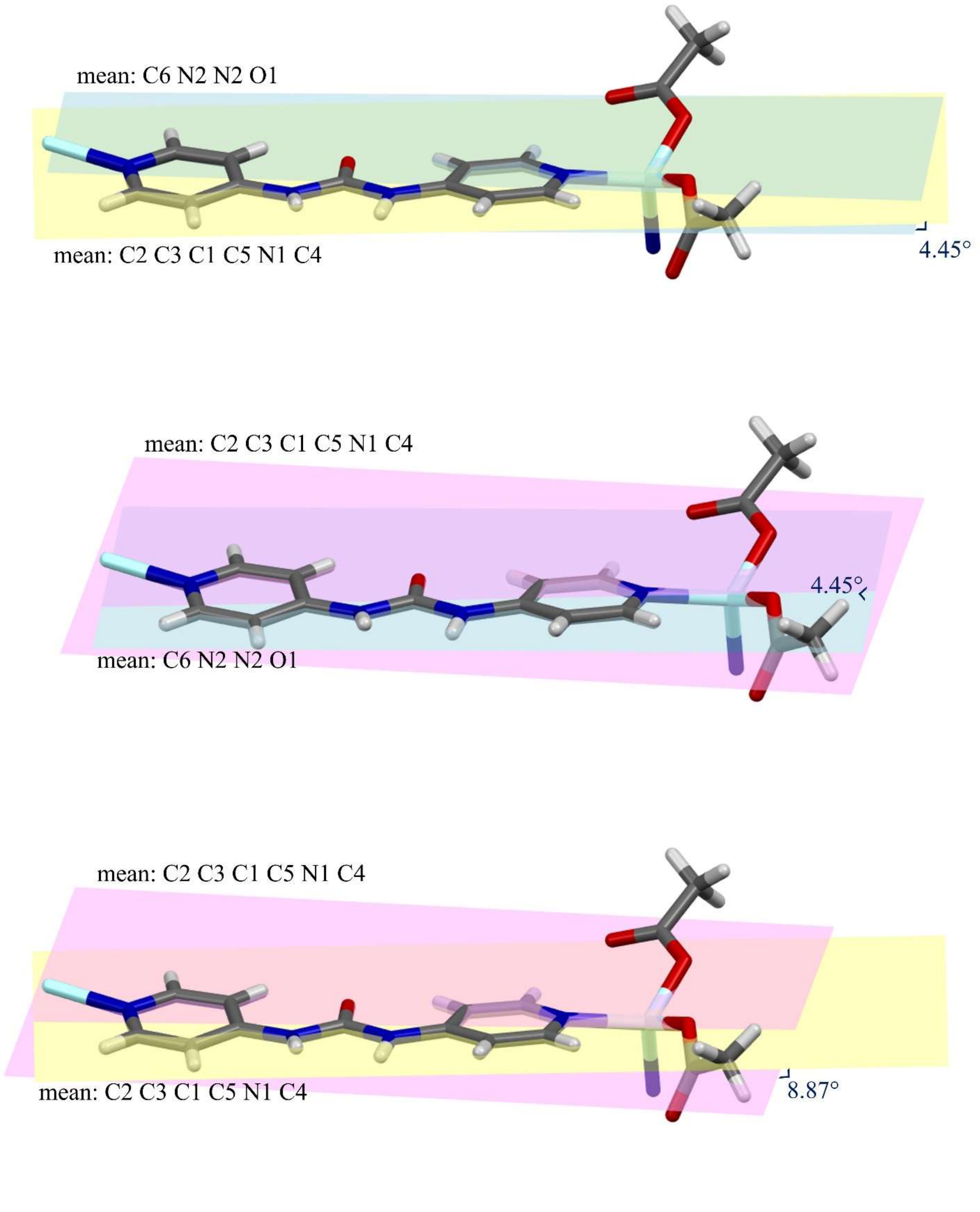

Figure S15. Angles between pyridyl-urea and pyridyl-pyridyl planes of ligand in compound **3**.

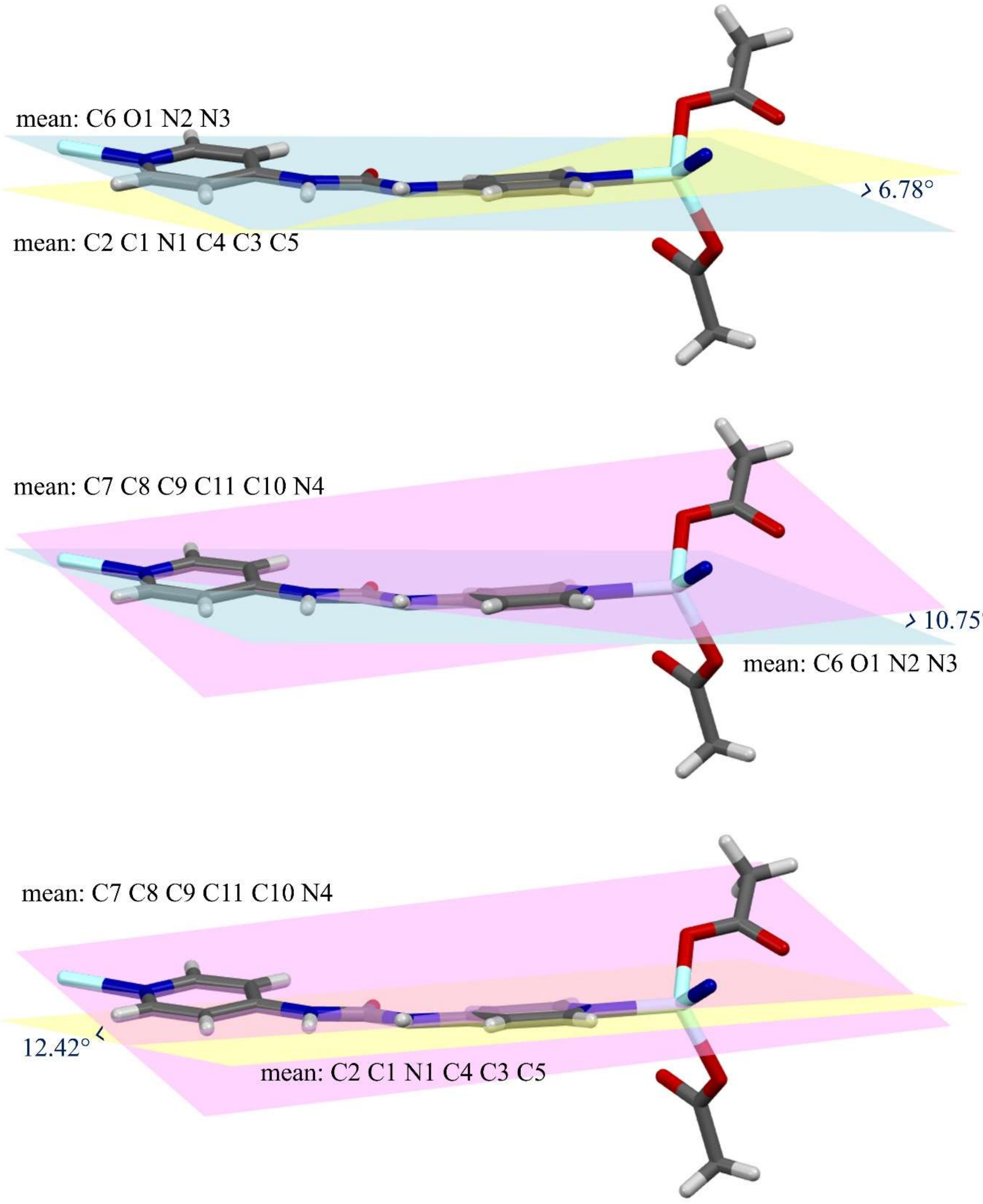

Figure S16. Angles between pyridyl-urea and pyridyl-pyridyl planes of ligand in compound **4**.

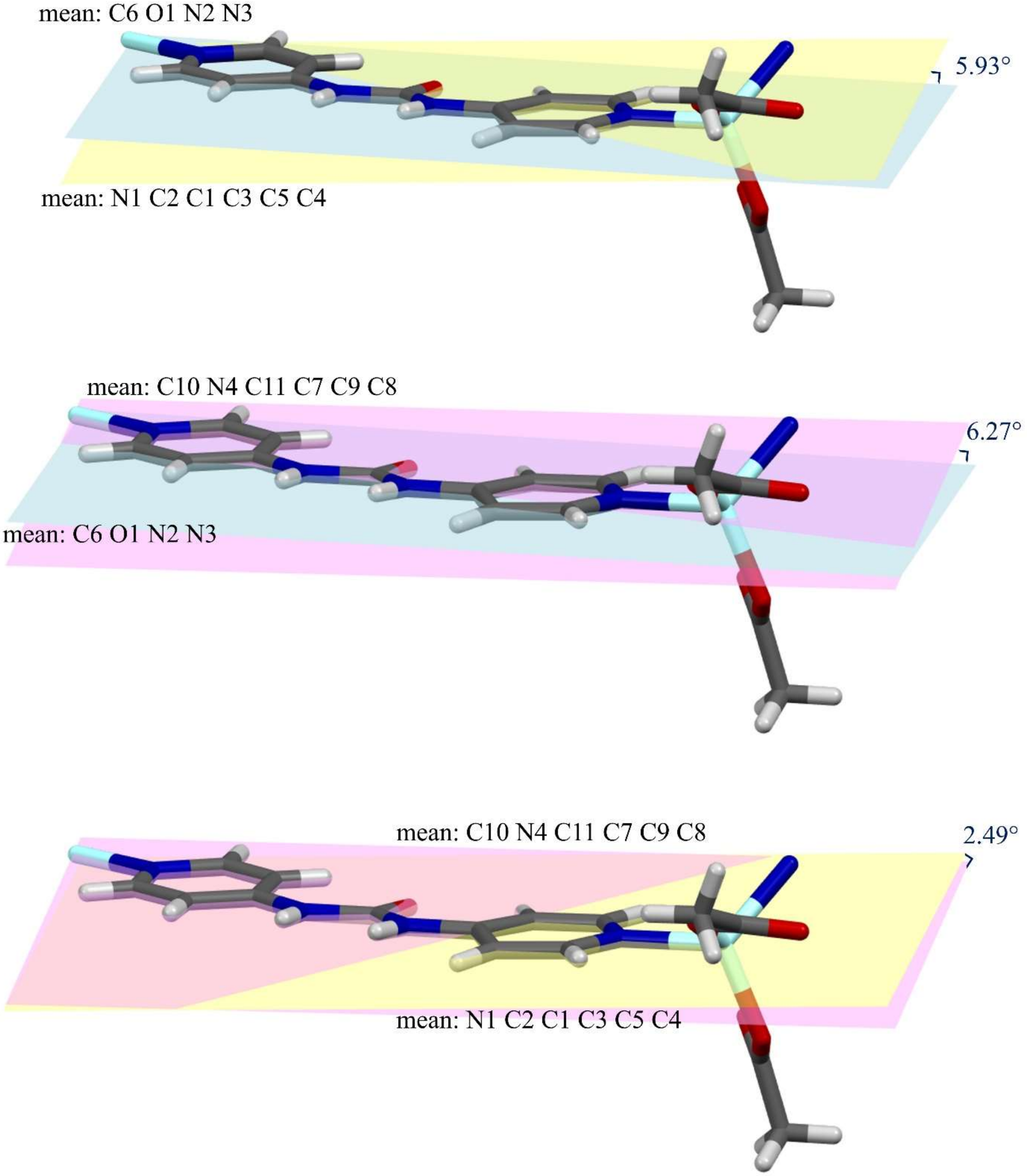

Figure S17. Angles between pyridyl-urea and pyridyl-pyridyl planes of ligand in compound **1α**.

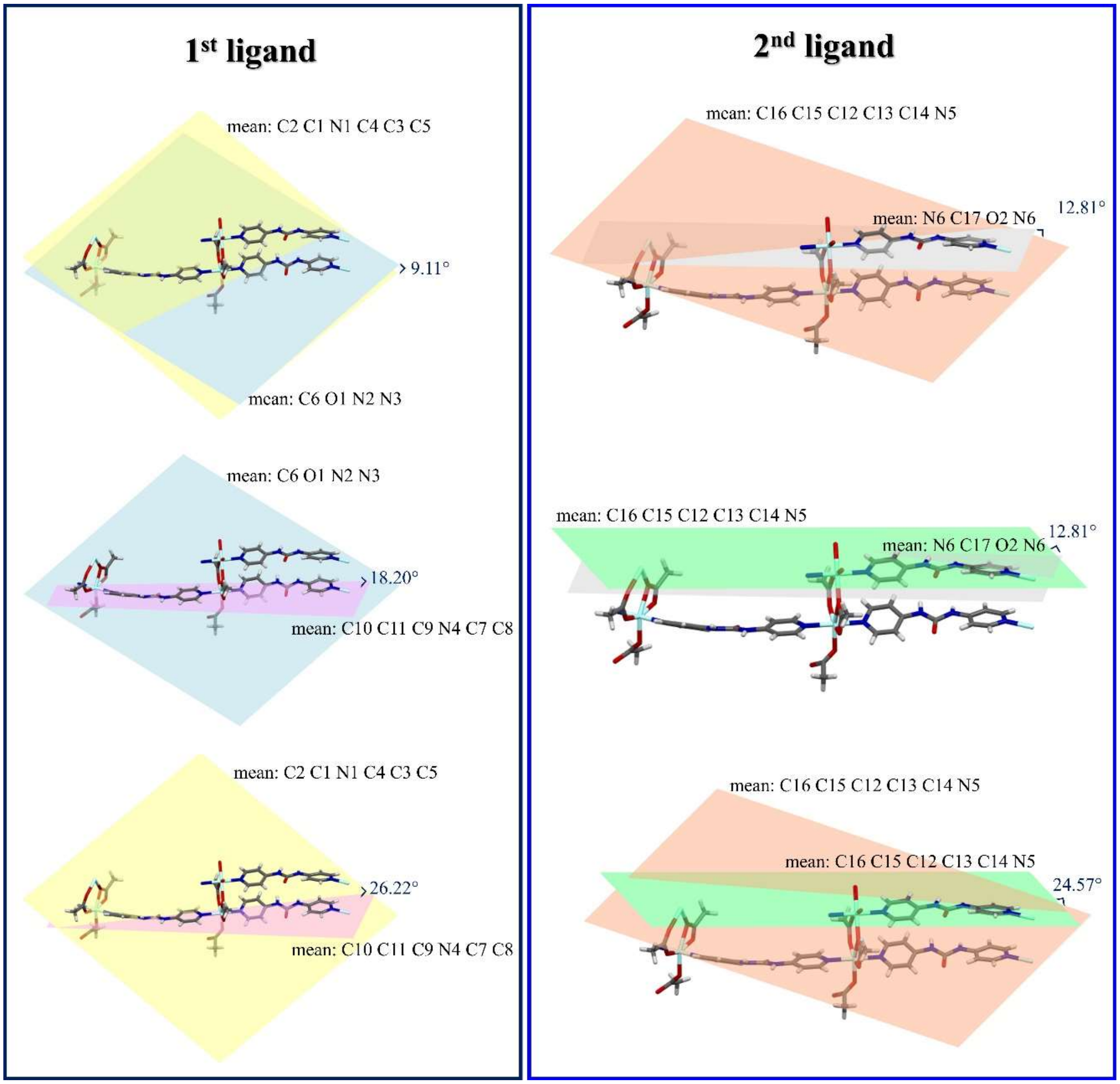

Figure S18. Angles between pyridyl-urea and pyridyl-pyridyl planes of ligands in compound **1β**.

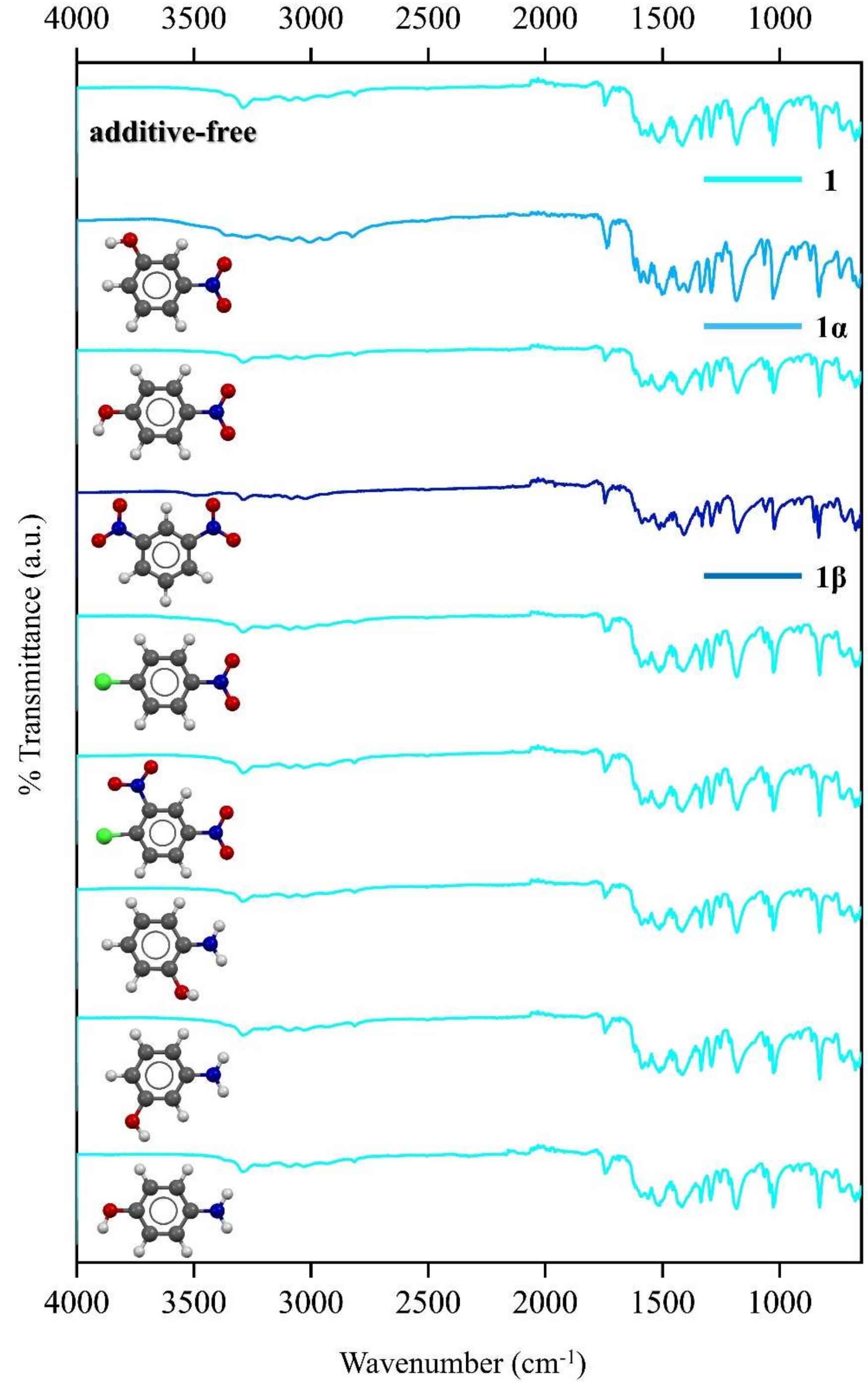

Figure S19. Comparison between ATR-FTIR spectra of compound **1** and the compounds obtained in the presence of different additives: as-synthesized **1**, **1α** and **1β**.

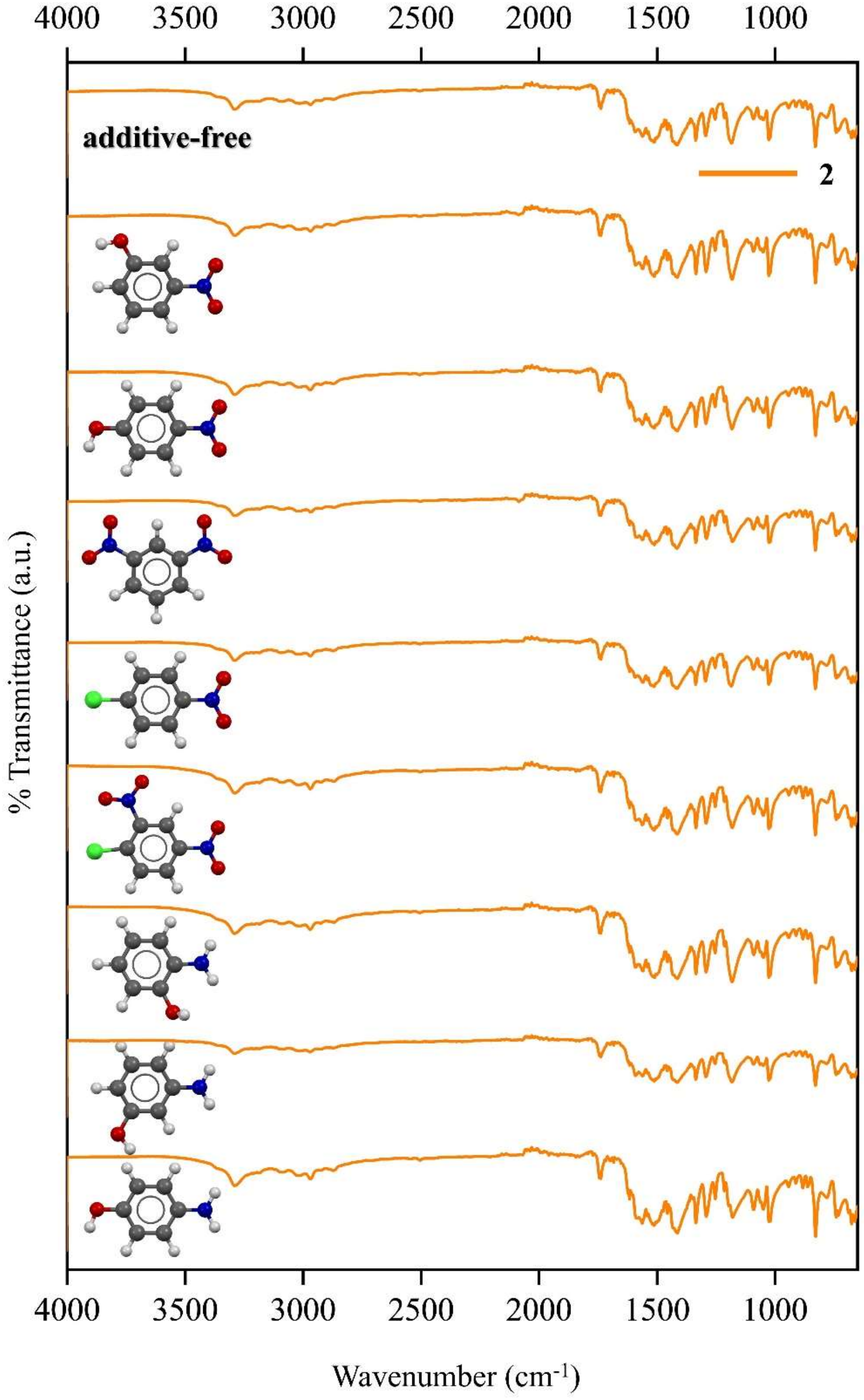

Figure S20. Comparison between ATR-FTIR spectra of compound **2** and the compounds obtained in the presence of different additives: as-synthesized **2**.

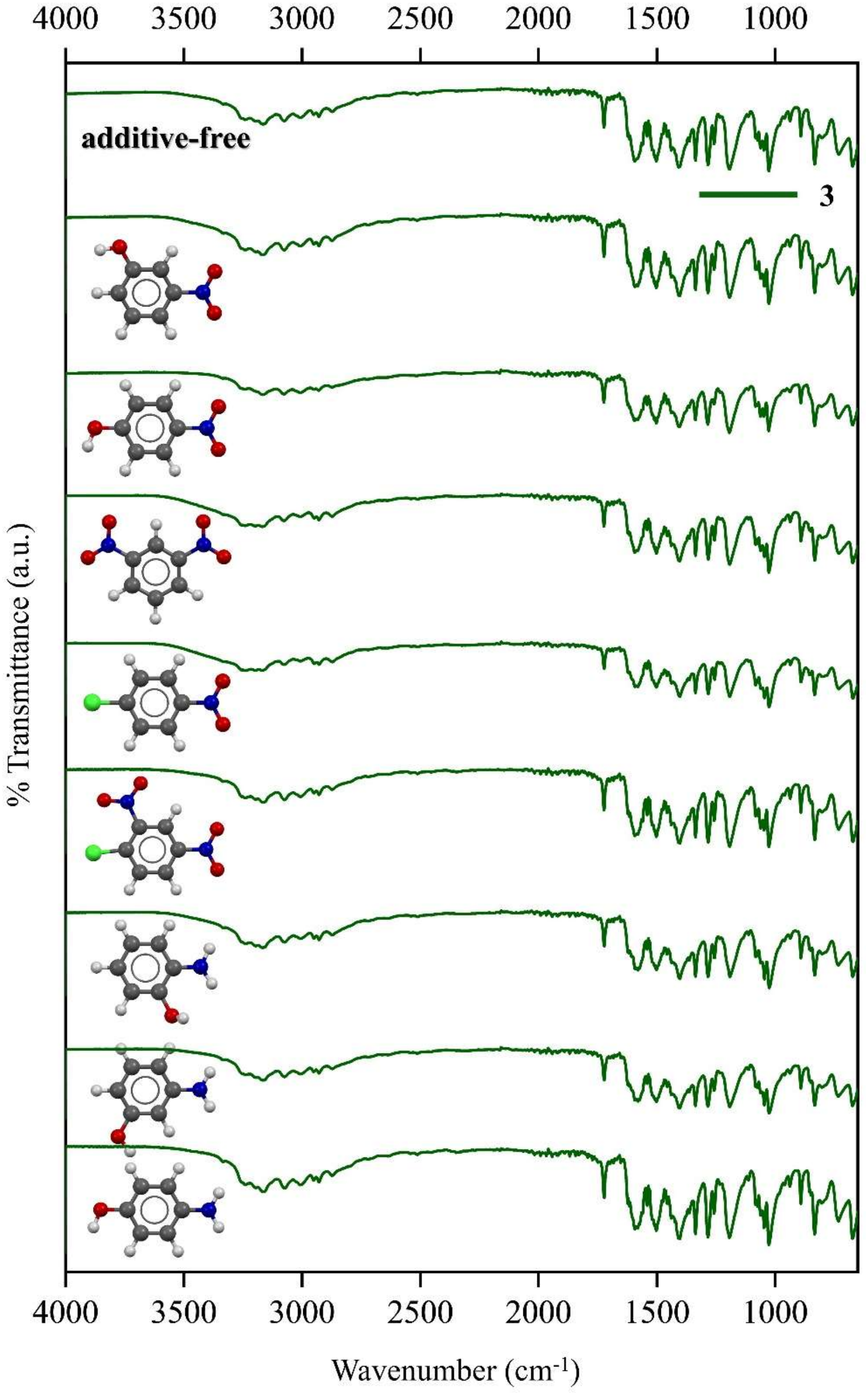

Figure S21. Comparison between ATR-FTIR spectra of compound **3** and the compounds obtained in the presence of different additives: as-synthesized **3**.

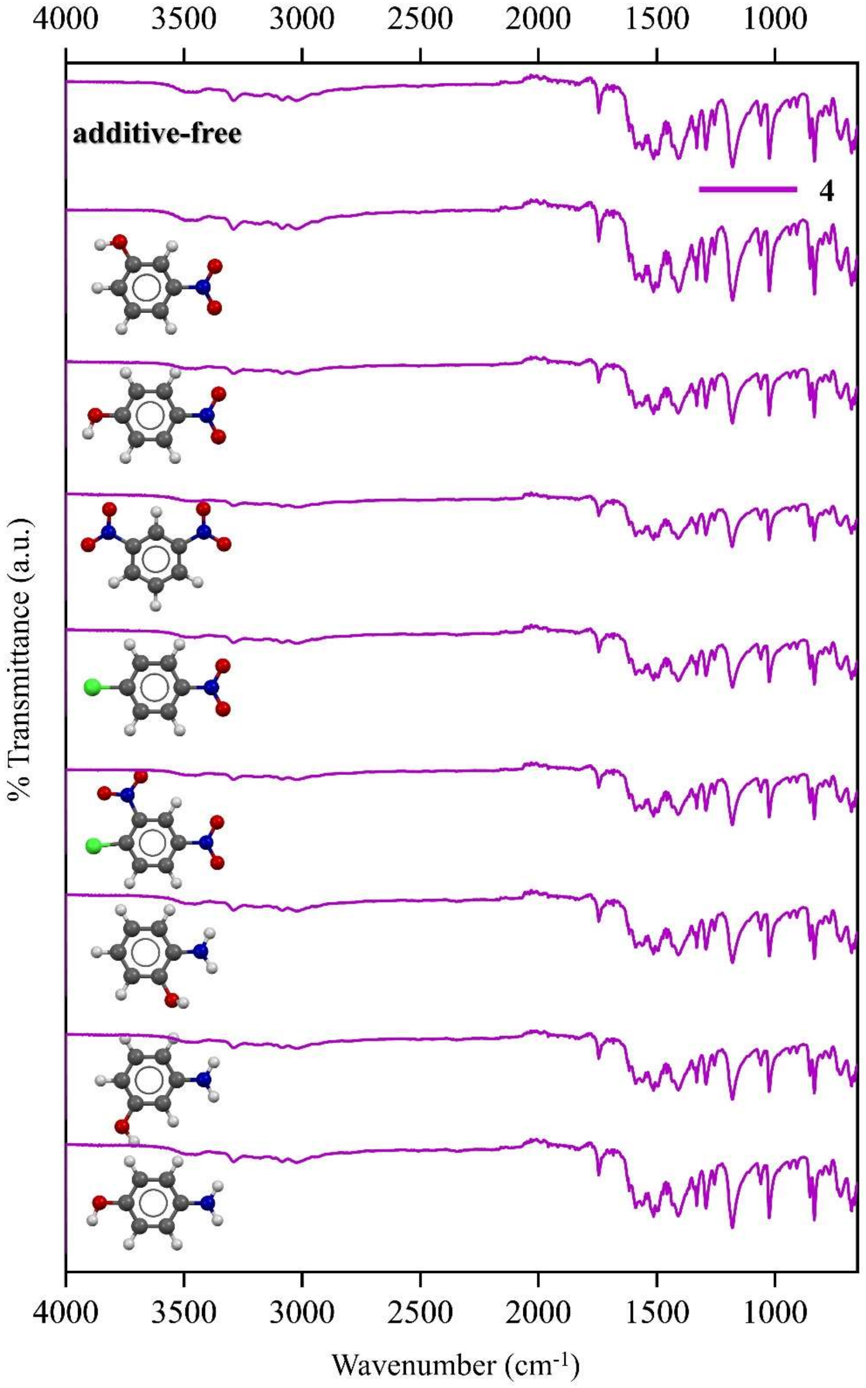

Figure S22. Comparison between ATR-FTIR spectra of compound **4** and the compounds obtained in the presence of different additives: as-synthesized **4**.

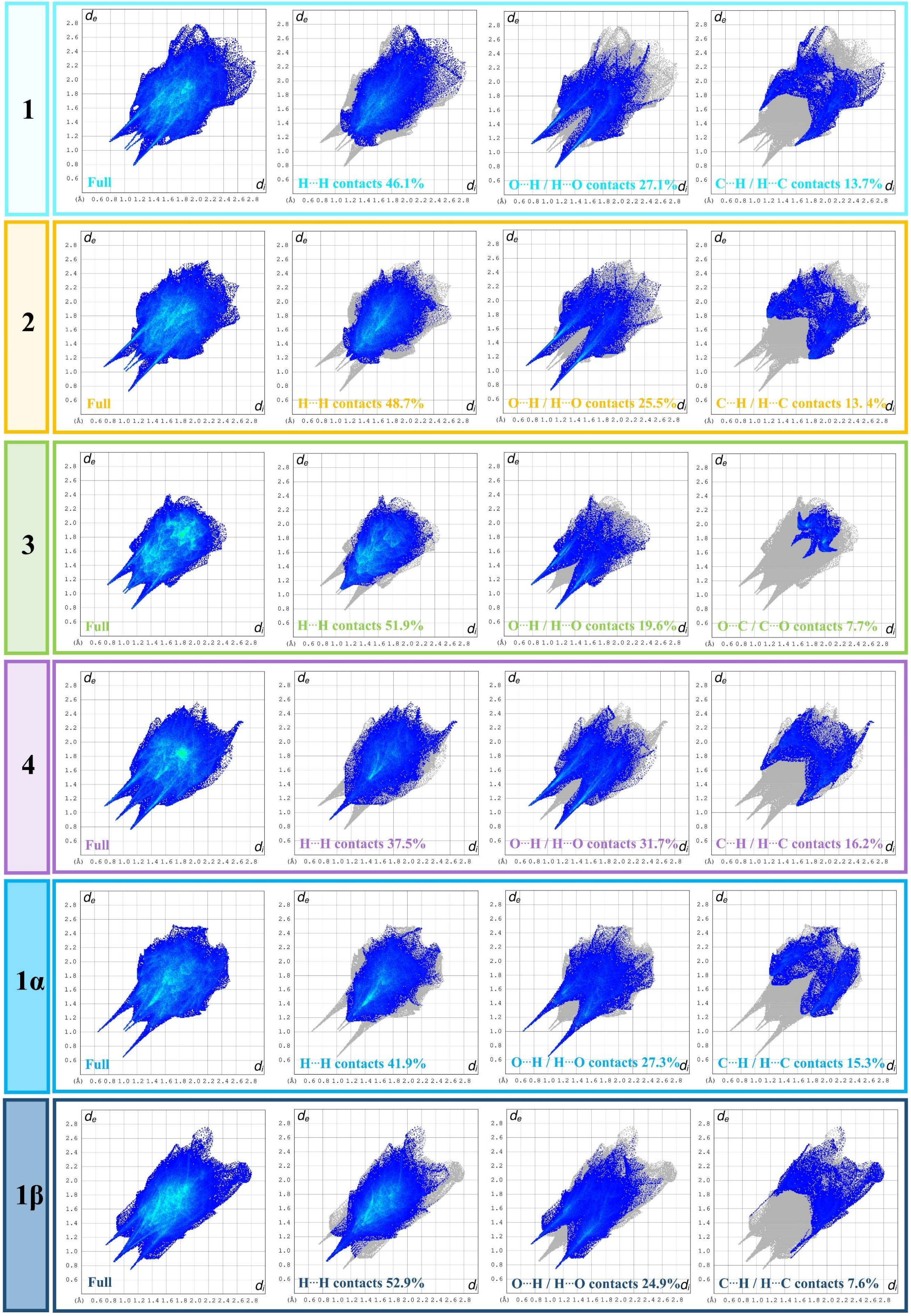

Figure S23. Fingerprint plots of compounds **1**-**4**, **1α** and **1β**, Full (left side) and resolved into H···H, O···H / H···O, and C···H / H···C contacts showing the percentages of contacts contributed to the total Hirshfeld surface area of the structures of presented compounds.

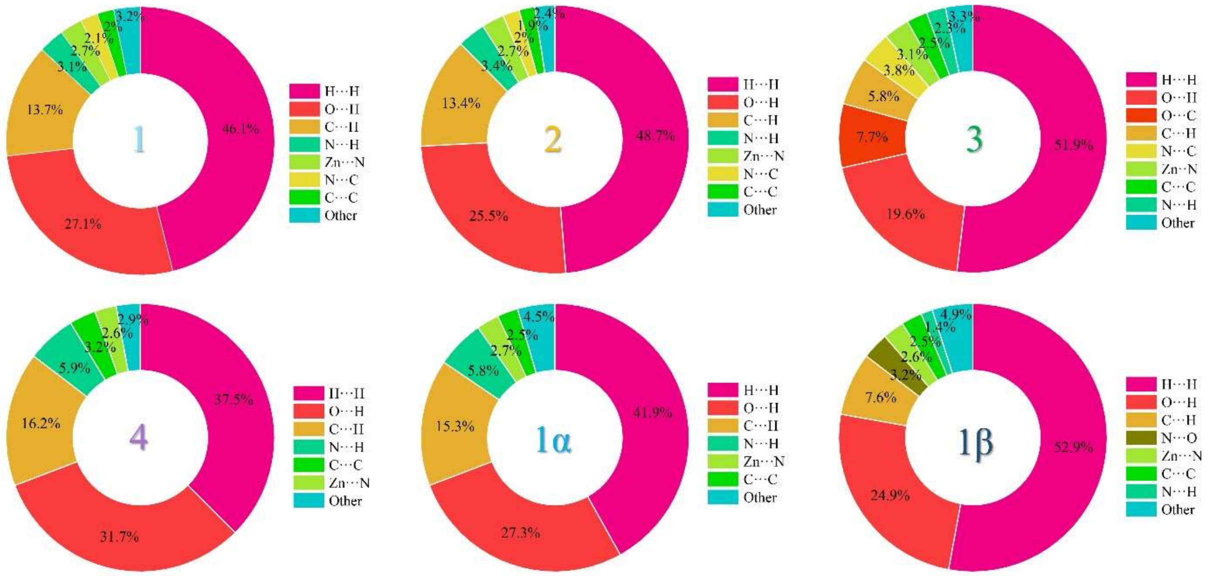

Figure S24. The relative contribution of different intermolecular interactions to the Hirshfeld surface area in the presented compounds.

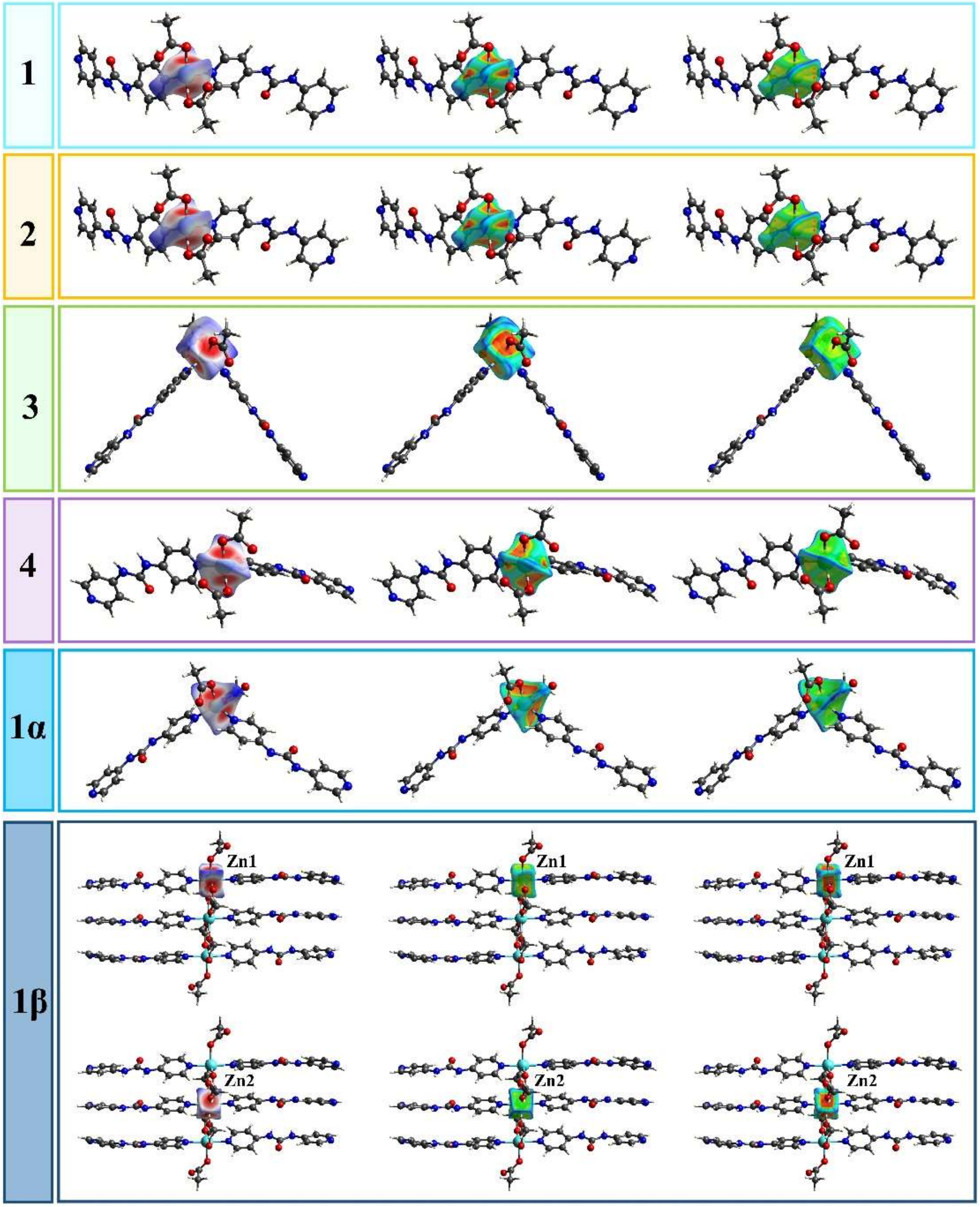

Figure S25. Views of Hirshfeld surfaces for the zinc center in the presented compounds mapped over d_{norm} (left), shape index (middle) and curvedness (right). For compound **1β** the Hirshfeld surfaces for the zinc centers, Zn1(top row) and Zn2 (bottom row) are presented.

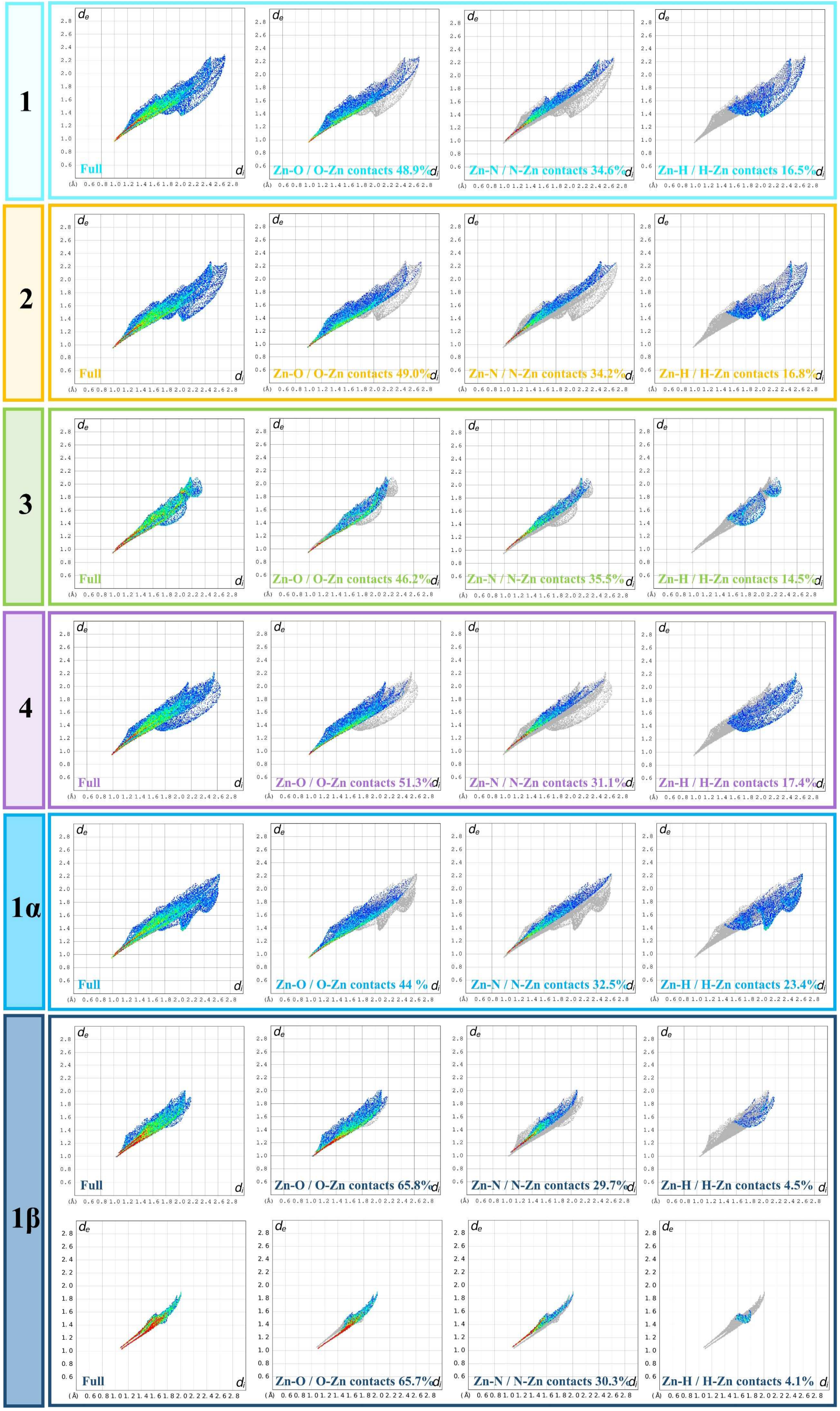

Figure S26. Comparison of the fingerprint plots calculated for the zinc center, in the presented compounds. For compound **1β** the fingerprint plots for the zinc centers, Zn1(top row) and Zn2 (bottom row) are presented.

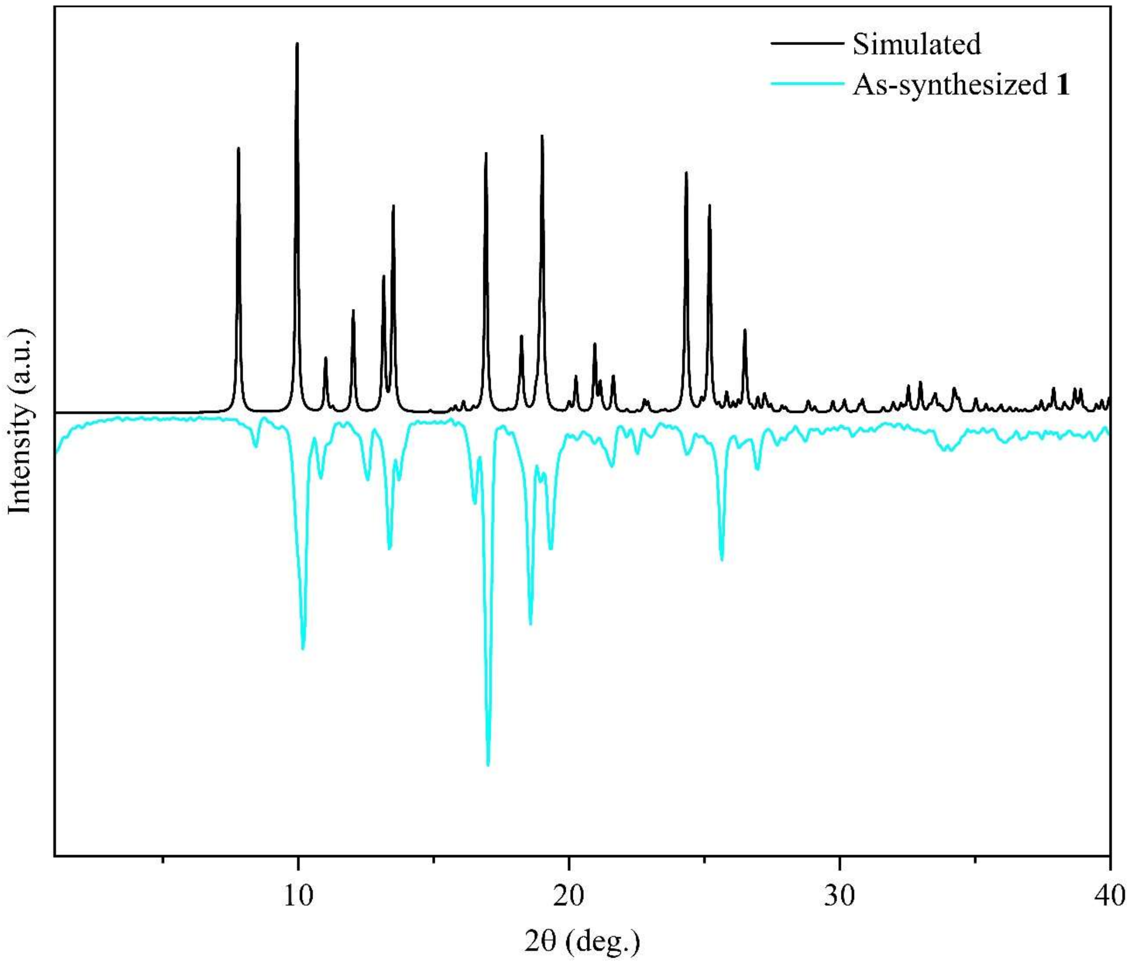

Figure S27. PXRD patterns of **1**. Black: Simulated from the X-ray single-crystal data; Cyan: observed for the as-synthesized solids.

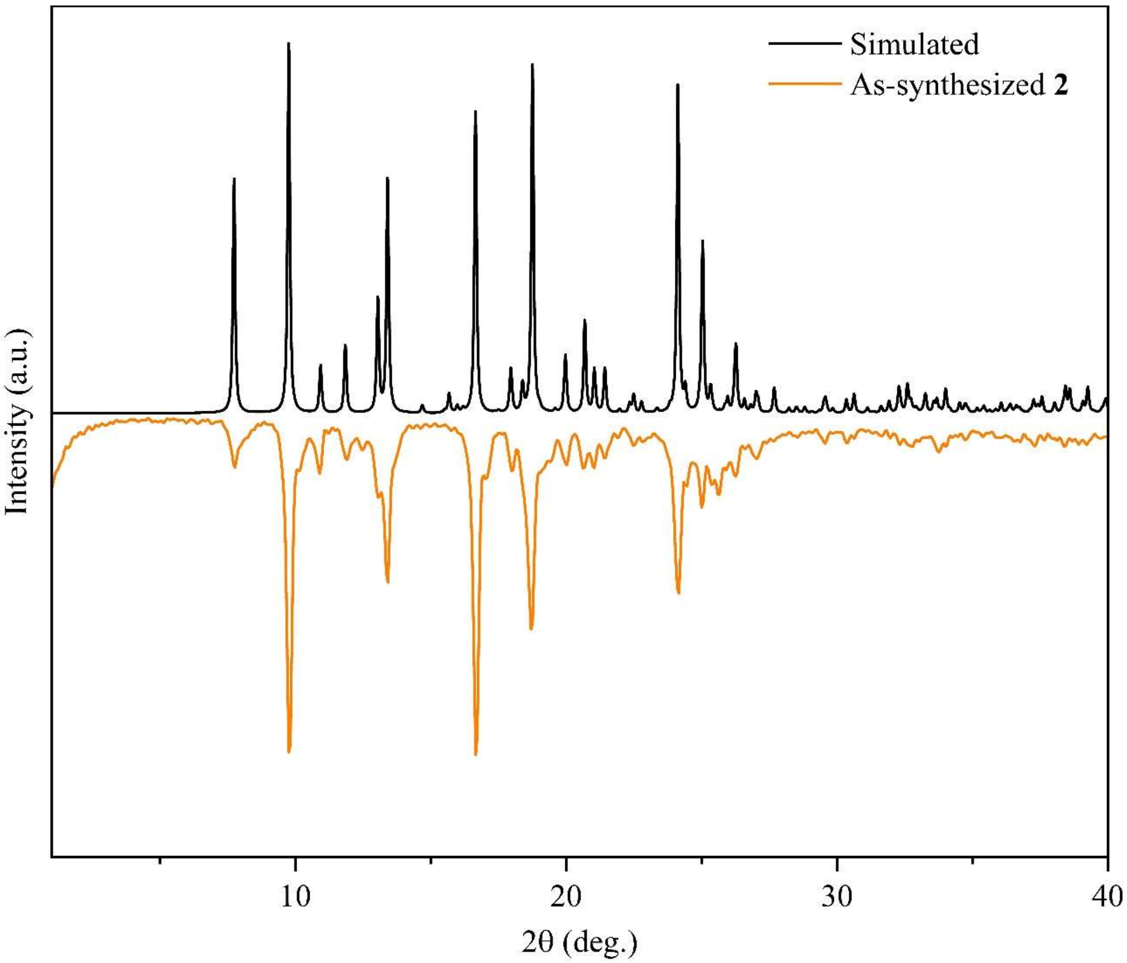

Figure S28. PXRD patterns of **2**. Black: Simulated from the X-ray single-crystal data; Orange: observed for the as-synthesized solids.

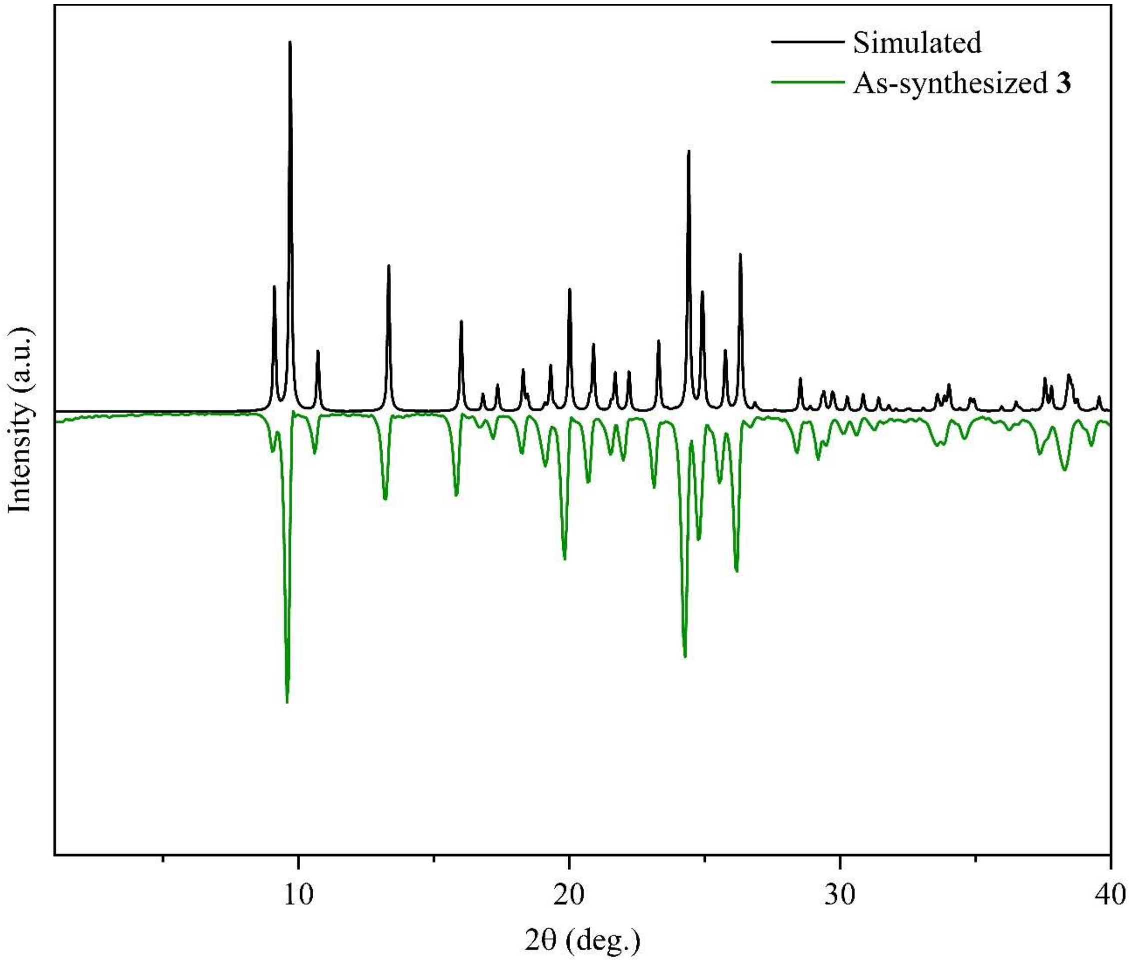

Figure S29. PXRD patterns of **3**. Black: Simulated from the X-ray single-crystal data; Green: observed for the as-synthesized solids.

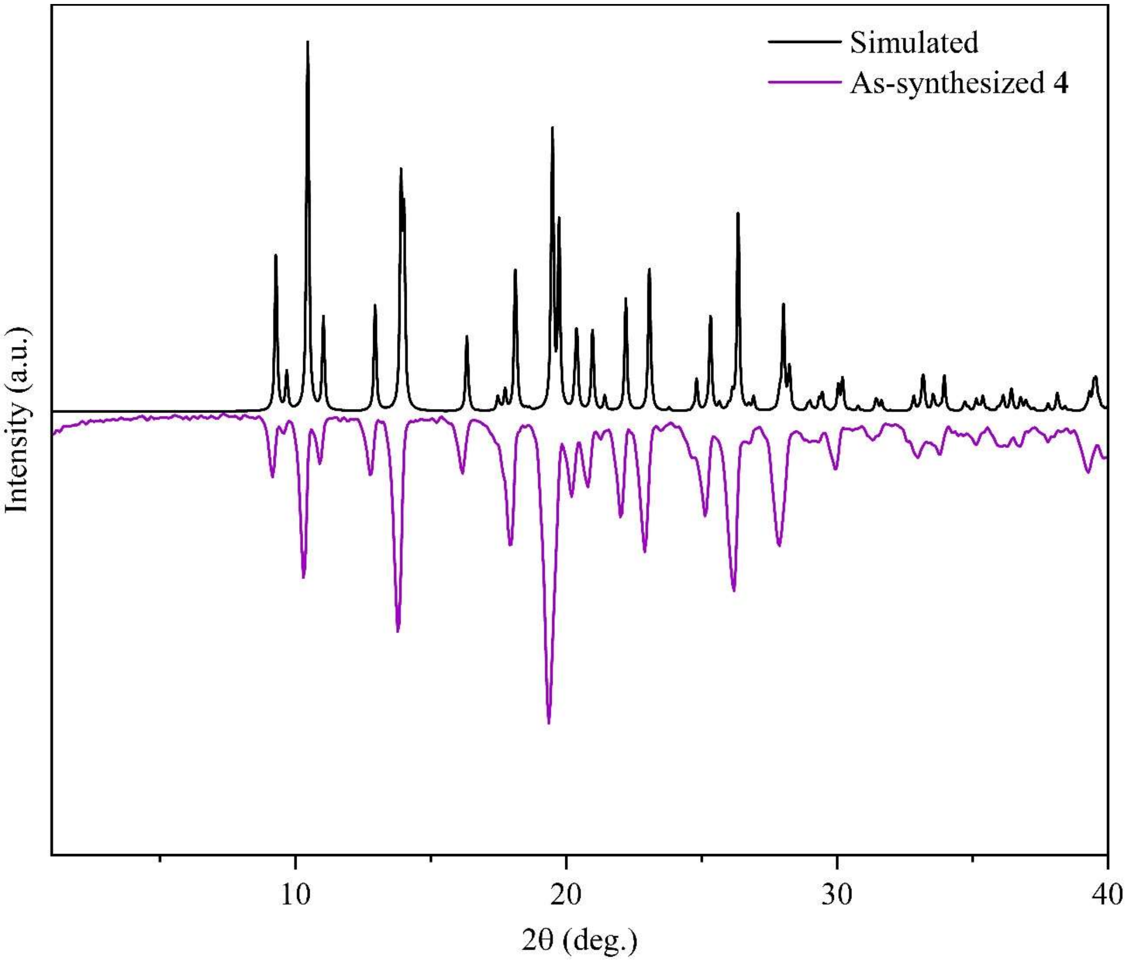

Figure S30. PXRD patterns of **4**. Black: Simulated from the X-ray single-crystal data; Purple: observed for the as-synthesized solids.

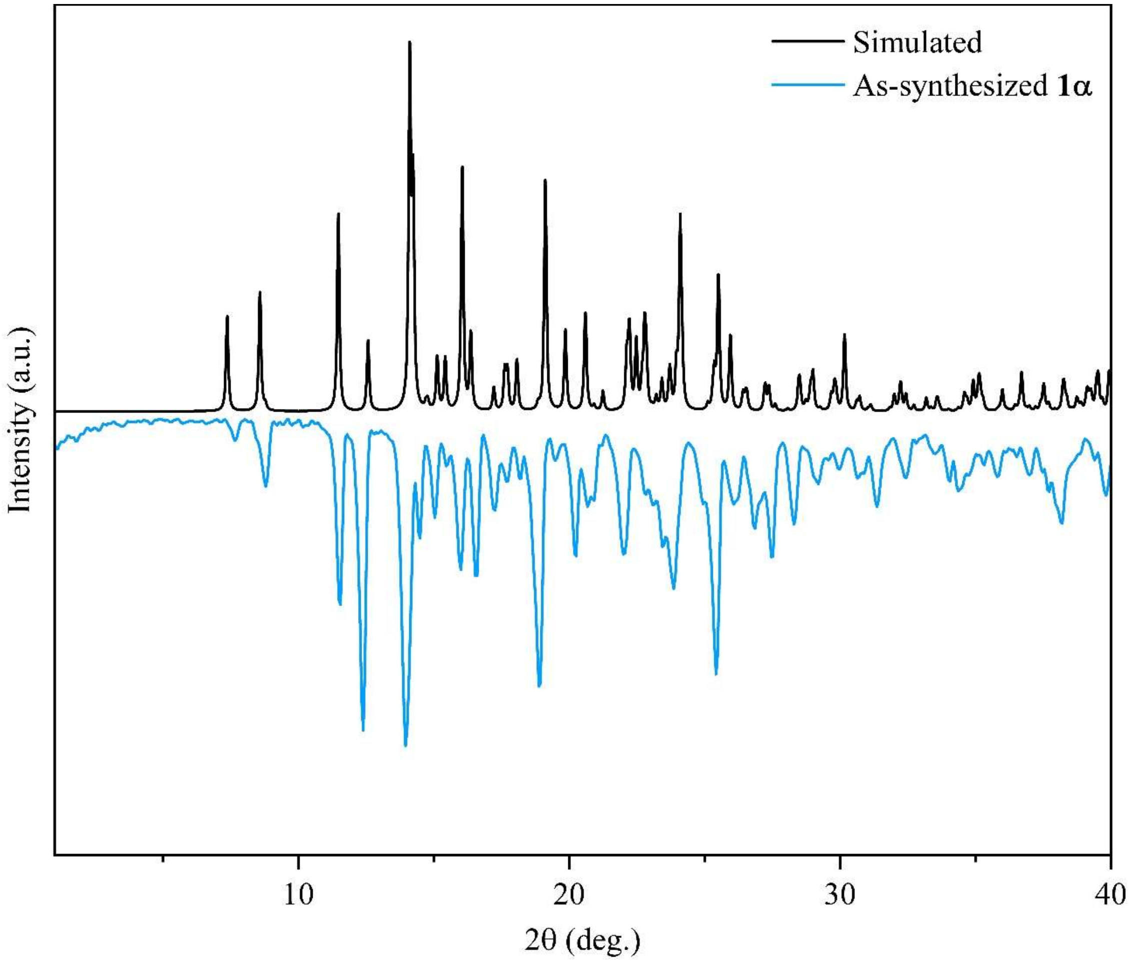

Figure S31. PXRD patterns of **1α**. Black: Simulated from the X-ray single-crystal data; Light blue: observed for the as-synthesized solids.

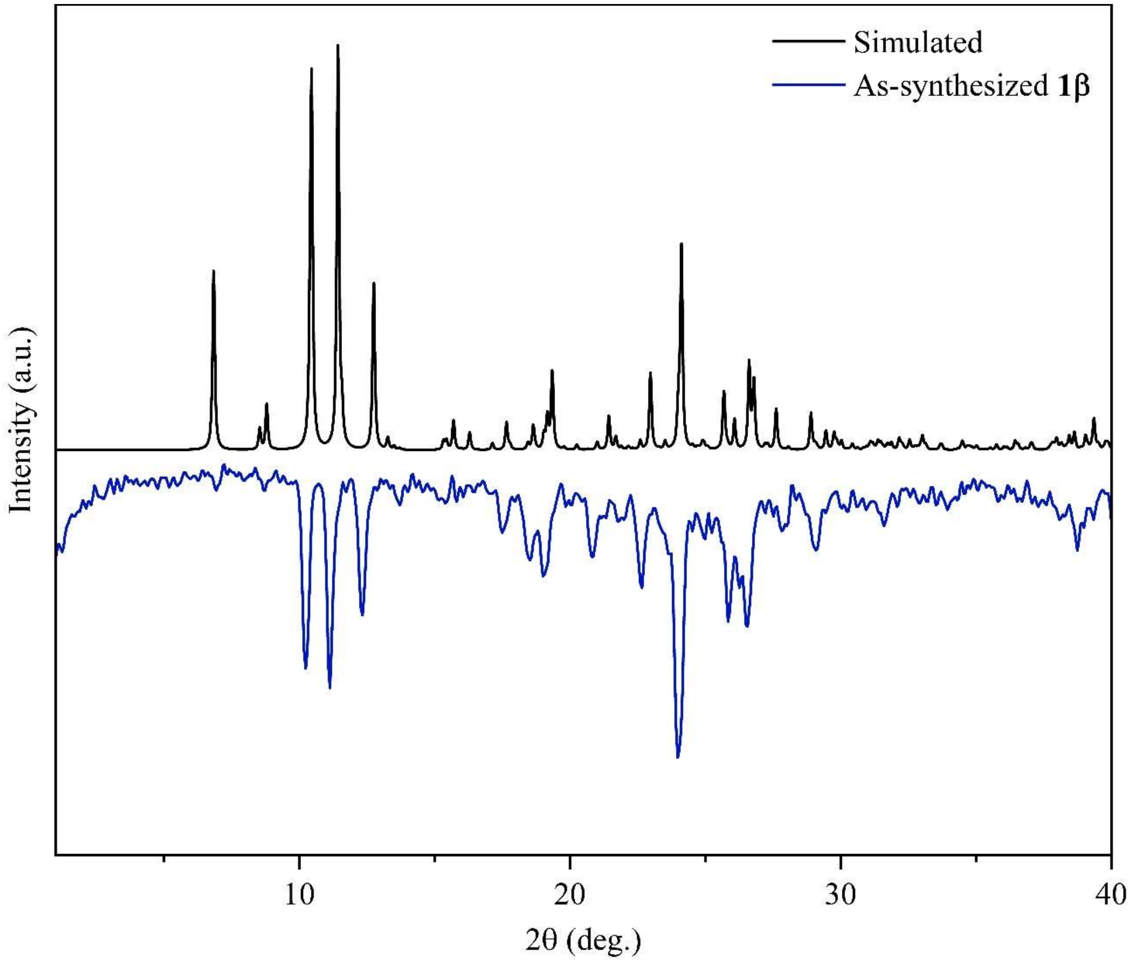

Figure S32. PXRD patterns of **1β**. Black: Simulated from the X-ray single-crystal data; Dark blue: observed for the as-synthesized solids.

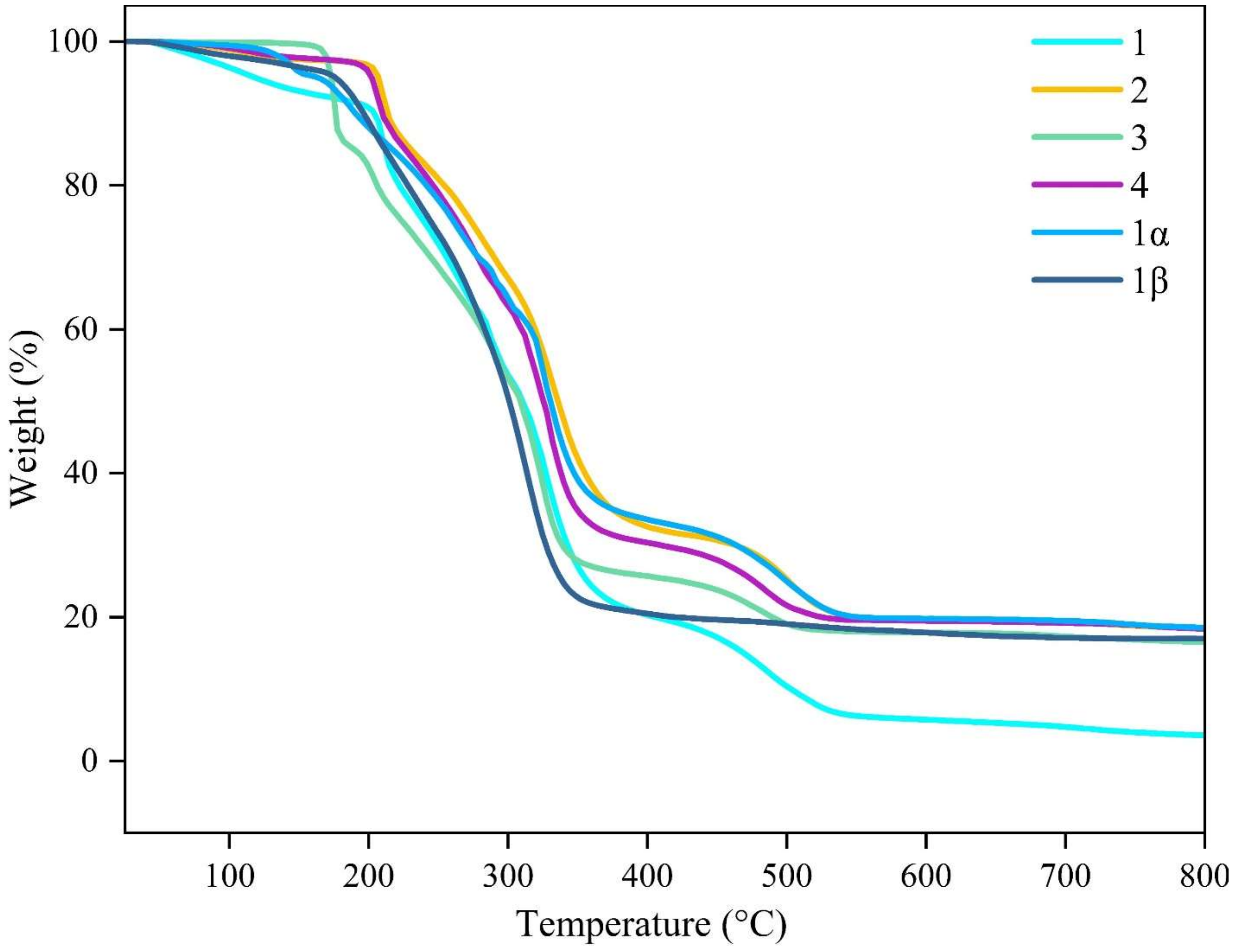

Figure S33. TGA curves of compounds **1**-**4**, **1α** and **1β**.

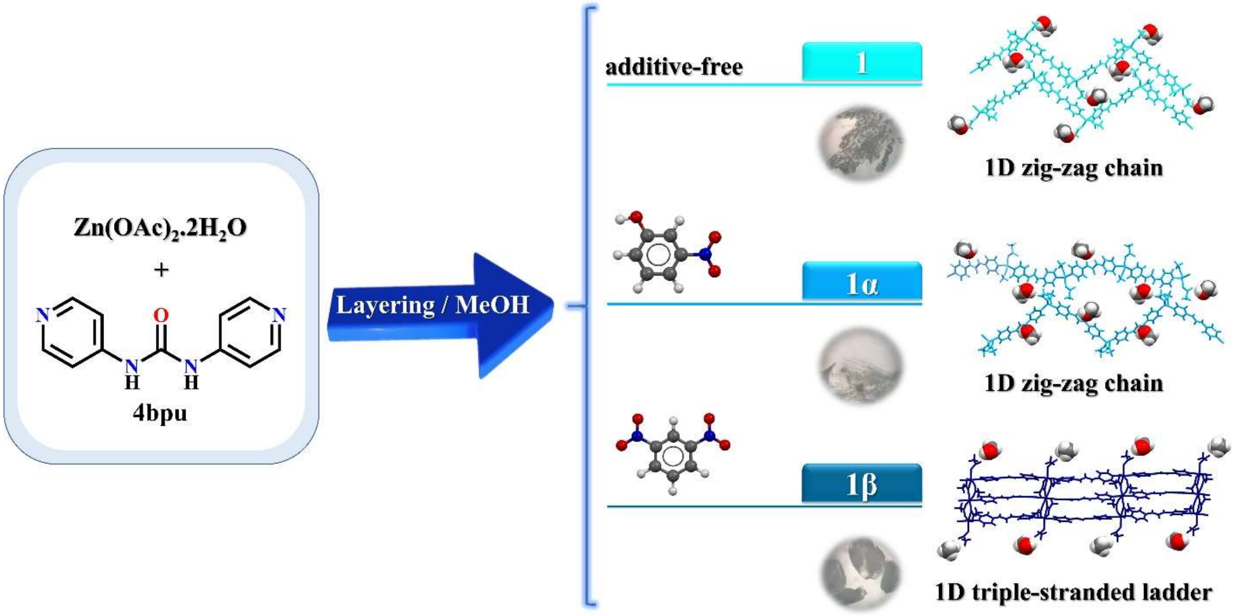

Scheme S1. Synthetic method, microscopic image and structural motifs for **1**, **1α** and **1β**.

Table S1. Selected bond lengths [Å] and angles [°] for **1**.

Zn(1)—O(4)	1.957(6)	O(4)-Zn(1)-N(1)	94.3(2)
Zn(1)—O(2)	1.998(5)	O(2)-Zn(1)-N(1)	100.2(2)
Zn(1)—N(1)	2.073(5)	O(4)-Zn(1)-N(4)$^{\#1}$	109.7(2)
Zn(1)—N(4)$^{\#1}$	2.030(5)	O(2)-Zn(1)-N(4)$^{\#1}$	97.7(2)
O(4)-Zn(1)-O(2)	143.5(2)	N(1) -Zn(1)-N(4)$^{\#1}$	107.2(2)
Symmetry code: #1: 3/2−x,−1/2+y,1/2−z.			

Table S2. Selected bond lengths [Å] and angles [°] for **2**.

Zn(1)—O(4)	1.997(7)	O(4)-Zn(1)-N(1)	97.3(3)
Zn(1)—O(2)	1.946(8)	O(2)-Zn(1)-N(1)	111.4(3)
Zn(1)—N(1)	2.047(7)	O(4)-Zn(1)-N(4)$^{\#1}$	98.5(3)
Zn(1)—N(4)$^{\#1}$	2.081(7)	O(2)-Zn(1)-N(4)$^{\#1}$	93.8(3)
O(4)-Zn(1)-O(2)	143.9(3)	N(1) -Zn(1)-N(4)$^{\#1}$	106.7(3)
Symmetry code: #1: −1/2−x, −1/2+y, 3/2−z.			

Table S3. Selected bond lengths [Å] and angles [°] for **3**.

Zn(1)—O(2)	1.928(3)	O(2)-Zn(1)-N(1)$^{\#1}$	121.44(16)
Zn(1)—O(2)$^{\#1}$	1.928(4)	N(1) -Zn(1)-O(2)$^{\#1}$	121.44(16)
Zn(1)—N(1)	2.026(4)	N(1) -Zn(1)-O(2)	104.0(16)
Zn(1)—N(1)$^{\#1}$	2.026(4)	N(1)$^{\#1}$ -Zn(1)-O(2)$^{\#1}$	104.0(16)
O(2)-Zn(1)-O(2)$^{\#1}$	101.9	N(1) -Zn(1)-N(1)$^{\#1}$	105.4(2)
Symmetry code: #1: **1** −x, y, 1/2−z.			

Table S4. Selected bond lengths [Å] and angles [°] for **4**.

Zn(1)—O(4)	1.931(7)	O(4)-Zn(1)-N(1)	103.2(3)
Zn(1)—O(2)	1.945(8)	O(2)-Zn(1)-N(1)	114.6(4)
Zn(1)—N(1)	2.047(7)	O(4)-Zn(1)-N(4)$^{\#1}$	109.8(3)
Zn(1)—N(4)$^{\#1}$	2.054(7)	O(2)-Zn(1)-N(4)$^{\#1}$	99.8(3)
O(4)-Zn(1)-O(2)	125.8(4)	N(1) -Zn(1)-N(4)$^{\#1}$	101.0(3)
Symmetry code: #1: 1/2+x, 1/2−y, −1/2 + z.			

Table S5. Selected bond lengths [Å] and angles [°] for **1α**.

Zn(1)—O(4)	1.966(9)	O(4)-Zn(1)-N(1)	95.6(4)
Zn(1)—O(2)	1.929(8)	O(2)-Zn(1)-N(1)	111.4(4)
Zn(1)—N(1)	2.054(9)	O(4)-Zn(1)-N(4)$^{\#1}$	108.4(4)
Zn(1)—N(4)$^{\#1}$	2.028(10)	O(2)-Zn(1)-N(4)$^{\#1}$	114.4(5)
O(4)-Zn(1)-O(2)	110.0(4)	N(1) -Zn(1)-N(4)$^{\#1}$	115.3(4)
Symmetry code: #1: 1/2+x, −y, −1/2 + z.			

Table S6. Selected bond lengths [Å] and angles [°] for **1β**.

Zn(1)—O(3)	2.068(6)	O(7)-Zn(1)-O(3)	105.2(2)
Zn(1)—O(5)	2.145(4)	O(7)-Zn(1)-N(1)	89.92(18)
Zn(1)—O(6)	2.312(5)	O(3)-Zn(1)-N(1)	88.6(2)
Zn(1)—O(7)	2.033(4)	O(7)-Zn(1)-O(5)	157.7(2)
Zn(1)—N(1)	2.134(5)	O(3)-Zn(1)-O(5)	97.1(2)
Zn(1)—N(4)$^{\#1}$	2.151(5)	N(1)-Zn(1)-O(5)	90.38(19)
Zn(2)—O(6)	2.129(4)	O(6)$^{\#2}$-Zn(2)-O(6)	180.0(2)
Zn(2)—O(6)$^{\#2}$	2.129(4)	O(6)$^{\#2}$-Zn(2)-N(5)	88.50(17)
Zn(2)—O(8)	2.136(5)	O(6)-Zn(2)-N(5)	91.50(17)
Zn(2)—O(8)$^{\#2}$	2.136(5)	O(6)$^{\#2}$-Zn(2)N(5)$^{\#2}$	91.50(17)
Zn(2)—N(5)	2.132(5)	O(6)-Zn(2)-N(5)$^{\#2}$	88.50(17)
Zn(2)—N(5)$^{\#2}$	2.132(5)	N(5)-Zn(2)-N(5)$^{\#2}$	180.0
O(7)-Zn(1)-N(4)$^{\#1}$	88.13(18)	O(6)$^{\#2}$-Zn(2)-O(8)$^{\#2}$	92.49(17)
O(3)-Zn(1)-N(4)$^{\#1}$	90.9(2)	O(6)-Zn(2)-O(8)$^{\#2}$	87.51(17)
N(1)-Zn(1)-N(4)$^{\#1}$	177.8(2)	N(5)-Zn(2)-O(8)$^{\#2}$	89.54(18)
O(5)-Zn(1)-N(4)$^{\#1}$	91.83(18)	N(5)$^{\#2}$-Zn(2)-O(8)$^{\#2}$	90.46(18)
O(7)-Zn(1)-O(6)	99.94(16)	O(6)$^{\#2}$-Zn(2)-O(8)	87.51(17)
O(3)-Zn(1)-O(6)	154.5(2)	O(6)-Zn(2)-O(8)	92.49(17)
N(1)-Zn(1)-O(6)	86.71(18)	N(5)-Zn(2)-O(8)	90.46(18)
O(5)-Zn(1)-O(6)	57.88(16)	N(5)$^{\#2}$-Zn(2)-O(8)	89.54(18)
N(4)$^{\#1}$-Zn(1)-O(6)	94.64(18)	O(8)$^{\#2}$-Zn(2)-O(8)	180.0(3)
Symmetry codes: #1: −1/2+x, −y + 1/2, −1/2 + z #2: 1/2−x, −y + 1/2, +1/2 −z.			

Table S7. Bond valence sums for zinc ions in compounds **1–4**, **1α** and **1β**.

Compound	Atom(s)	Valance state	Bond valance sum. (BVS)	Deviation %
**1**	Zn1	2	1.775	11
**2**	Zn1	2	1.763	12
**3**	Zn1	2	1.966	2
**4**	Zn1	2	1.880	6
**1α**	Zn1	2	1.877	6
**1β**	Zn1	2	1.921	4
	Zn2	2	1.912	4

Table S8. Hydrogen bonds geometry (D−H···A) for **1**, **1α** and **1β**.

Compound	D−H···A	d(D−H) / Å	d(H···A) / Å	d(D···A) / Å	∠ D−H···A / deg.
**1**	N2−H2A···O3$^{\#1}$	0.78(2)	2.08(10)	2.768(7)	146(17)
	N3−H3A···O3$^{\#1}$	0.79(2)	2.11(6)	2.850(7)	157(15)
	O6−H6···O5$^{\#2}$	0.91(2)	1.81(3)	2.716(11)	174(15)
	C15−H15B$^{\#2}$···O2	0.96	2.925	3.711	139.93
Symmetry codes: #1: –x + 2, –y + 2, –z; #2: –x + 3/2, y + 1/2, –z + 1/2					
**1α**	N2−H2A···O6$^{\#1}$	0.86	2.58	3.327(19)	145.2
	N3−H3A···O6$^{\#1}$	0.86	1.95	2.809(18)	172.5
	O6−H6$^{\#1}$···O5$^{\#2}$	0.87(2)	1.76(8)	2.613(15)	167.2(2)
	C4−H4···O2$^{\#2}$	0.93	2.715	3.279	119.78
	C13−H13C···O4$^{\#3}$	0.96	3.202	3.655	110.80
Symmetry codes: #1: –x + 1, y – 1/2, –z + 3/2; #2: –x, y – 1/2, –z + 1/2; #3: x, y + 1, z					
**1β**	O9−H9A···O4$^{\#1}$	0.85	1.78	2.450(11)	133.8
	N2−H2A···O4$^{\#2}$	0.86	2.35	3.126(8)	151.1
	N3−H3A···O4$^{\#2}$	0.86	1.99	2.826(8)	165.1
	C23−H23B$^{\#2}$···O1	0.96	2.859	3.713	148.66
	C19−H19C$^{\#3}$···O1$^{\#4}$	0.96	2.56	3.417(11)	148.7
Symmetry codes: #1: x, y, z + 1; #2: –x + 1, –y + 1, –z; #3: x + 1/2, –y + 1/2, z + 1/2; #4: –x + 3/2, y + 1/2, –z + 1/2					

Table S9. Hydrogen bonds geometry (D−H···A) for **2−4**.

Compound	D−H···A	d(D−H) / Å	d(H···A) / Å	d(D···A) / Å	∠ D−H···A / deg.
**2**	N2−H2A···O5#1	0.87(2)	2.09(11)	2.869(9)	148(19)
	N3−H3A···O5#1	0.95(19)	1.9(2)	2.750(10)	155(18)
	O6−H6A#1···O3#1	0.82	1.93	2.745(17)	170.3
	C13−H13A···O3#2	0.96	2.683	3.538	148.67
Symmetry codes: #1: x – 1/2, –y + 1/2, z + 1/2 #2: x – 1/2, –y + 1/2, z – 1/2					
**3**	N2−H2A···O4#1	0.85(2)	2.05(2)	2.889(6)	170(4)
	C3−H3···O4#1	0.93	2.54	3.276(7)	136.3
	C9−H9A#1···O1#2	0.97	2.639	3.314	127.02
	C8−H8B···O3#3	0.96	2.59	3.502(8)	159.7
	O4−H4A···O3	0.86(2)	2.01(5)	2.728(6)	141(7)
Symmetry codes: #1: –x + 1, –y, –z #2: x, –y –1, z – 1/2 #3: x, y +1, z					
**4**	N2−H2A···O5#1	0.83(11)	2.03(11)	2.797(10)	154(11)
	N3−H3A···O5#1	0.83(11)	2.13(11)	2.873(10)	148(11)
	O6−H6#2···O3#1	0.87(12)	1.93(12)	2.787(13)	166(14)
	C4−H4···O6#2	0.93	2.42	3.165(12)	137
	C15−H15C···O3#3	0.96	2.673	3.504	145.07
	O6−H6#4···O3#3	0.87(12)	1.93(12)	2.787(13)	166(14)
	O6−H6···O3#4	0.87(12)	1.93(12)	2.787(13)	166(14)
	C2−H2···O4#3	0.93	2.52	3.240(11)	134.9
Symmetry codes: #1: −x + 3/2, y + 1/2, −z + 3/2 #2: x + 1/2, y – 1/2, z #3: –x + 1, y, –z + 3/2 #4: x, y – 1, z					

Table S10. Structural tranformation tests for **1–4**, **1α** and **1β**.

	**Immersed in MeOH**	**Immersed in EtOH**	**Immersed in etylene glycol**	**Immersed in propylene glycol**
**1**	-	DRST - **4**	DRST - **3**	Undissolved and became opaque - **1**
**2**	DRST - **4**	-	DRST - **3**	Undissolved and became opaque - **2**
**3**	Undissolved and remained transparent - **3**	Undissolved and remained transparent - **3**	-	Dissolved and did not form new crystals.
**4**	Undissolved and became opaque - **4**	Undissolved and became opaque - **4**	Undissolved and became opaque - **4**	-
**1α**	DRST - **4**	Undissolved and became opaque - **1α**	DRST - **3**	Undissolved and became opaque - **1α**
**1β**	Undissolved and became opaque - **1β**	Undissolved and became opaque - **1β**	Undissolved and became opaque - **1β**	Undissolved and became opaque - **1β**

Table S11. Geometrical parameters of spodium bonds in **1–4** and **1α**.

**Spodium bond**		**1**		**2**		**3**		**4**		**1α**
**Zn1⋯A**	A	O3	O5	O3	O5	O3	O3	O3	O5	O3
	[Å]	2.633	2.630	2.589	2.664	3.075	3.075	2.589	2.783	2.685
**Y−Zn1⋯A**	Y, A	N4, O3	N1, O5	N4, O3	N1, O5	O2, O3	O2, O3	N4, O3	N1, O5	O4, O3
	[°]	150.5	147.4	146.9	150.0	140.5	140.5	151.8	154.3	160.1

X-ray crystallography

The crystallographic data for **1**-**4**, **1α** and **1β** were collected on a STOE IPDS-II diffractometer with graphite-monochromated Mo-Kα radiation (λ = 0.71073 Å). Single crystals with appropriate dimensions were chosen under a polarizing microscope and were mounted onto a glass fiber for data collection. Cell constants and orientation matrices for data collection were obtained by least-square refinement of the diffraction data. All diffraction data were collected at 298(2) K to a maximum 2θ value of 50.00° in a series of ω scans in 1° oscillations and was integrated using the Stoe X-AREA[2] software package. A numerical absorption correction was applied using X-RED[3] and X-SHAPE[4] software. The reflection data were corrected for Lorentz and polarizing effects. The structures were solved by direct methods[5] and subsequent difference Fourier maps and then refined on F^2 by a full-matrix least-squares procedure using anisotropic displacement parameters.[6] All the hydrogen atoms attach to carbon atoms in **1**-**4, 1α** and **1β** were added in idealized positions. The atomic factors were taken from International Tables for X-ray Crystallography.[7] All refinements were performed using the X-STEP32 crystallographic software package.[8]

References

1. Chandran, S. K., Nath, N. K., Cherukuvada, S. & Nangia, A. N–H…N(pyridyl) and N–H…O(urea) hydrogen bonding and molecular conformation of N-aryl-N′-pyridylureas. *J. Mol. Struct.* **968**, 99-107, (2010).
2. X-AREA:. Program for the Acquisition and Analysis of Data, version 1.30 (Darmstadt, Germany, 2005).
3. X-RED:. Program for Data Reduction and Absorption Correction, version 1.28b (Darmstadt, Germany, 2005).
4. X-SHAPE:. Program for Crystal Optimization for Numerical Absorption Correction, version 2.05 (Darmstadt, Germany, 2004).
5. Sheldrick, G. M. SHELX97; Program for Crystal Structure Solution, (University of Göttingen: Göttingen Germany, 1997).
6. Sheldrick, G. M. SHELX97; Program for Crystal Structure Refinement, (University of Göttingen: Göttingen Germany, 1997).
7. *International Tables for X-ray Crystallography*, (V. C. Dordrecht, The Netherlands, Kluwer Academic Publisher, 1995).
8. X-STEP32:. Crystallographic Package, version 1.07b (Darmstadt, Germany, 2000).
